# Mannose supports lung cancer metabolism during glycolytic limitation

**DOI:** 10.1038/s44319-026-00874-6

**Published:** 2026-08-07

**Authors:** Theresa Haitzmann, Niklas B Thompson, Hardik Shah, Katharina Schindlmaier, Joseph Jelwan, Thomas O Eichmann, Nia G Hammond, Maia G Clare, Robert B Cameron, Brandon Faubert, Katharina Leithner

**Affiliations:** 1 Division of Pharmacology, Otto Loewi Research Center, Medical University of Graz, Graz, Austria; 2 Metabolomics Platform, Comprehensive Cancer Center, The University of Chicago, Chicago, IL USA; 3 Core Facility Mass Spectrometry, Medical University of Graz, Graz, Austria; 4 Department of Medicine, Section of Hematology and Oncology, The University of Chicago, Chicago, IL USA; 5 Lung Research Cluster, Medical University of Graz, Graz, Austria; 6 BioTechMed-Graz, Graz, Austria

**Keywords:** Cancer, Metabolism

## Abstract

Cancer cells frequently show elevated glucose consumption to support proliferation and survival. This led to the assumption that glycolytic inhibitors could be effective in cancer treatment. However, barriers to clinical implementation remain. Adaptive strategies, such as metabolizing alternative nutrients, may play a role. Here, we investigated the use of an understudied sugar, mannose, in lung cancer cells and xenografts. Stable isotope tracing reveals enhanced contribution of mannose to GDP-mannose and GDP-fucose, key glycosylation precursors, upon treatment with the glycolytic inhibitor 2-deoxyglucose (2-DG) or glucose starvation in vitro. Mannose restores the glucose-withdrawal-induced decrease of GDP-mannose and GDP-fucose pools, and partially rescues proliferation upon 2-DG treatment or glucose deprivation. ^13^C_6_-mannose infusion in patient-derived xenograft mice reveals a considerable contribution of mannose to GDP-mannose and GDP-fucose in tumors, which is further enhanced by 2-DG. In normal lungs, the pathway is only partially active. Mannose is also shuttled towards glycolysis in lung tumors in vivo and glucose-deprived cells in vitro. In conclusion, mannose utilization for glycosylation precursor synthesis represents an adaptive strategy in lung cancer cells under metabolic stress.

## Synopsis

Alternative nutrients might limit the efficacy of glycolytic intervention in cancer treatment. This study highlights the importance of mannose utilization for glycosylation precursors upon inhibition of glycolysis or glucose starvation.

- Mannose contributes to the glycosylation precursors GDP-mannose and GDP-fucose in lung cancer xenografts in vivo.
- Treatment with the glycolytic inhibitor 2-DG enhances mannose use for glycosylation precursor synthesis.
- Mannose is shuttled towards glycolysis in lung tumors in vivo and glucose deprived lung cancer cells in vitro.
- Exogenous mannose partially restores decreased proliferation rates and rescues ER stress of cancer cells treated with 2-DG.

## Abstract (web summary)

Alternative nutrients might limit the efficacy of glycolytic intervention in cancer treatment. This study highlights the importance of mannose utilization for glycosylation precursors upon inhibition of glycolysis or glucose starvation.

## Graphical abstract

**Figure:**
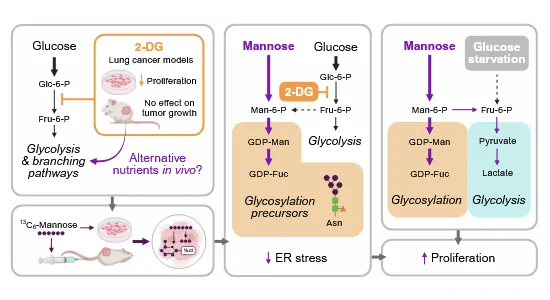

**Figure:**
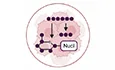

## Introduction

Lung cancer remains a global challenge, as despite therapeutic improvements, it is still the deadliest cancer type worldwide (Bray et al, 2024). An area of opportunity is leveraging the metabolic alterations of cancer cells in order to develop novel treatment options (Pavlova and Thompson, 2016; Faubert et al, 2020; Mendez-Lucas et al, 2020). The enhanced uptake and metabolism of glucose is a widely known metabolic feature of tumors (Warburg et al, 1927; Li et al, 2025). Glucose can be diverted to multiple biosynthetic pathways needed for cancer progression, including nucleotide synthesis and glycosylation reactions (Pavlova and Thompson, 2016). This has led to the assumption that glycolytic inhibitors could be a promising approach in cancer treatment. However, resistance to glycolysis inhibition is frequent, especially in vivo (Abdel-Wahab et al, 2019).

The glucose analogue 2-deoxyglucose (2-DG) is a widely studied glycolytic inhibitor. After uptake through glucose transporters and phosphorylation to 2-DG-6-phosphate, it cannot undergo further metabolism and therefore accumulates intracellularly, leading to glycolysis blockade (Xi et al, 2014). Furthermore, it can also interfere with N-linked glycosylation (Kurtoglu et al, 2007; Berthe et al, 2018; Pajak et al, 2019). In non-small cell lung cancer (NSCLC), 2-DG reduced subcutaneous tumor growth in the highly glycolytic squamous cell carcinoma. However, the growth of lung adenocarcinomas (LUAD) remained unaffected (Goodwin et al, 2017). This heterogenous response suggests that some NSCLC tumors can bypass glycolysis inhibition in vivo, highlighting the need to identify compensatory metabolic mechanisms that might limit efficacy.

Adaptation of cancer cells to their nutrient-variable and metabolically complex microenvironment, resulting from high rates of consumption and insufficient perfusion, may play a role in resistance towards glycolysis inhibition. Other nutrient sources, such as alternative sugars, might compensate for reduced glucose metabolism by supplying biosynthetic or glycolytic intermediates, thereby enabling resistance to glycolysis inhibition in vivo.

Besides glucose, the most abundant monosaccharides in plasma are mannose, fructose and galactose. Interestingly, mannose has been found to be elevated in the serum of lung cancer patients (Deja et al, 2014). Physiologically, mannose is obtained from the diet or released from peripheral tissues and is an important precursor used for glycosylation reactions (Alton et al, 1998; Sharma and Freeze, 2011), which are crucial for proper protein function. Mannose is taken up by cells via *SLC2A* glucose transporters and is phosphorylated by hexokinase to mannose-6-phosphate (Sharma et al, 2014). Upon phosphorylation, mannose can also be shuttled towards glycolysis, as has been shown in fibroblasts (Panneerselvam and Freeze, 1996; Ichikawa et al, 2014), osteosarcoma cells (Gonzalez et al, 2018) and lung cancer cells (Luciano-Mateo et al, 2026). Studies have shown that exogenous mannose can rescue effects caused by 2-DG treatment in cancer cell lines in vitro, including breast cancer (Berthe et al, 2018), rhabdomyosarcoma (Leon-Annicchiarico et al, 2015), and acute lymphoblastic leukemia (Gu et al, 2017). Additionally, mannose rescued protein glycosylation in lung cancer cells which was reduced by 2-DG (Luciano-Mateo et al, 2026). However, to the best of our knowledge, whether mannose is used for glycosylation and glycolysis in tumor cells in vivo remains unknown.

Here, we found that mannose considerably contributes to the generation of the glycosylation precursors GDP-mannose and GDP-fucose in lung cancer cells in vitro and xenograft models in vivo, which was enhanced by 2-DG treatment. Moreover, mannose enters the glycolysis pathway in lung tumors in vivo and glucose-deprived cells in vitro. Our study shows that mannose utilization for the generation of glycosylation precursors represents an adaptive strategy of cancer cells to respond to glycolysis inhibition or glucose restriction.

## Results

### The efficacy of 2-DG treatment in lung cancer cells is limited in the presence of mannose

**Figure 1:**
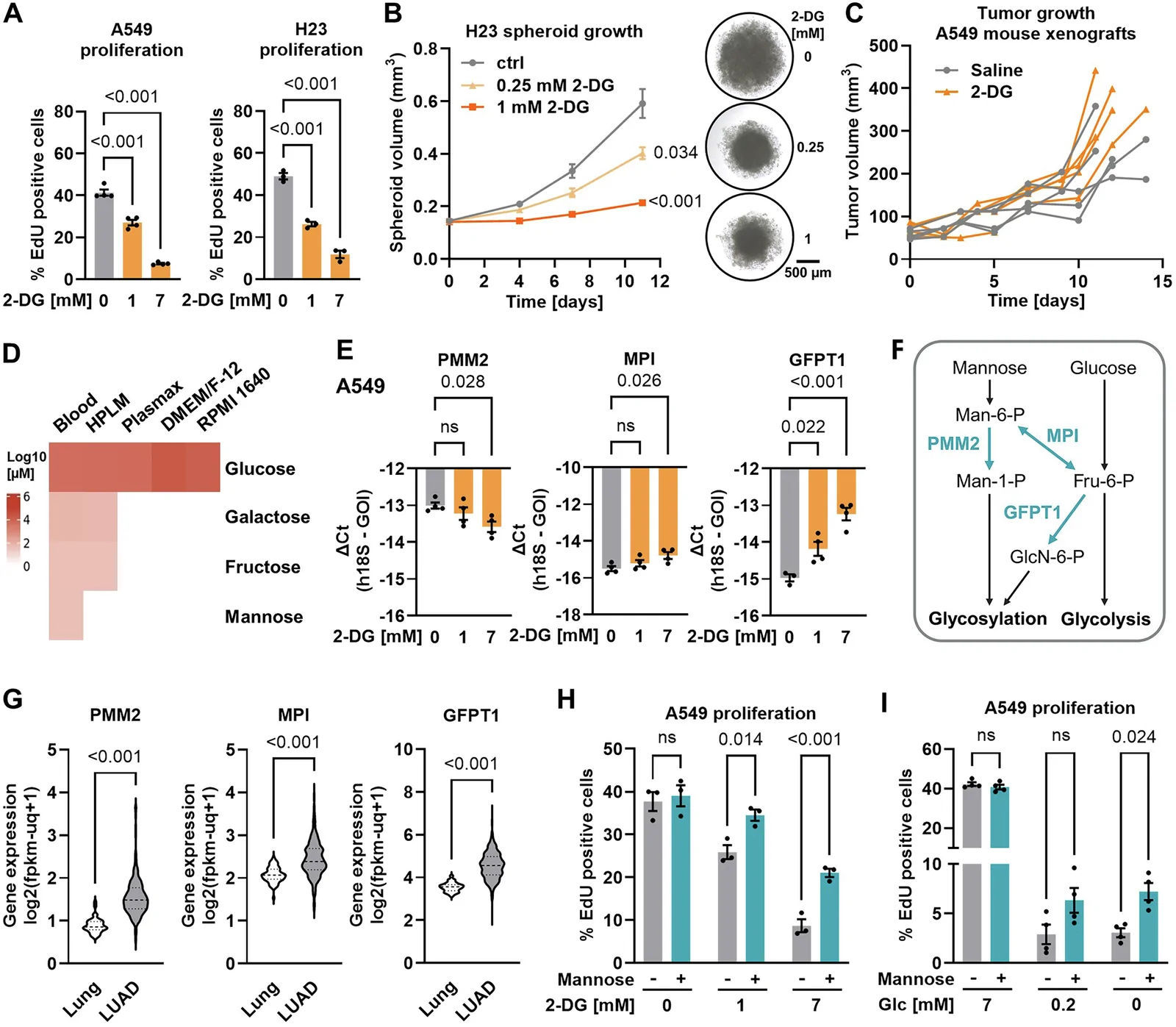
Mannose limits the efficacy of the glycolysis inhibitor 2-DG in lung cancer cells. (**A**) Proliferation rate (% EdU positive cells) of A549 and H23 cells treated with 2-deoxyglucose (2-DG) for 48 h (*n* = 4 for A549, *n* = 3 for H23). (**B**) Effect of 2-DG treatment on growth of H23 spheroids (*n* = 4). Representative images of spheroids on day eleven after treatment are shown. (**C**) Subcutaneous tumor growth of A549 mouse xenograft tumors treated with saline (control) or 2-DG (intraperitoneal injection of 500 mg/kg 3x/week for 2 weeks). Each line represents the growth pattern of an individual tumor. (**D**) Sugar (hexose) levels in blood (Human Metabolome Database, HMDB) and different cell culture media (log10 of µM concentration+1). (**E**) Expression of genes involved in mannose metabolism in A549 cells treated with 2-DG for 24 h. ΔCt-values were calculated as human 18S ribosomal RNA (h18S, reference gene) minus gene of interest (GOI) (*n* = 4). (**F**) Schematic of mannose and glucose fueled metabolic pathways. PMM2 phosphomannomutase 2, MPI mannose-6-phosphate isomerase, GFPT1 glutamine-fructose-6-phosphate transaminase 1, Man-6-P mannose-6-phosphate, Man-1-P mannose-1-phosphate, Fru-6-P fructose-6-phosphate, GlcN-6-P glucosamine-6-phosphate. (**G**) Gene expression in lung adenocarcinoma (LUAD; *n* = 528) vs non-involved lung tissue (*n* = 59) from the publicly available GDC TCGA LUAD dataset (https://www.cancer.gov/tcga). Data were obtained via the UCSC Xena platform (https://xena.ucsc.edu/). (**H**) Proliferation rate (% EdU positive cells) of A549 cells treated with 2-DG in combination +/− 100 µM mannose for 48 h (*n* = 3). (**I**) Proliferation rate (% EdU positive cells) of A549 cells treated with glucose starvation media in combination +/− 100 µM mannose for 48 h (*n* = 4). (**A**, **B**, **E**, **H**, **I**) Experiments were performed in media supplemented with a physiological glucose concentration (7 mM) and 10% dialyzed fetal bovine serum. Results are shown as mean +/− SEM from biological replicates (*n*). Statistical significance was determined by (**A**, **E**) one-way ANOVA with Dunnett’s post-hoc analysis, (**B**) two-way ANOVA of each treatment condition compared to control, (**G**) Mann–Whitney *U* test or (**H**, **I**) two-way ANOVA with Šidák’s multiple comparison test. *P* values are provided in the graphs; ns, not significant. Illustration in (**F**) was created in BioRender. Haitzmann, T. (2026) https://BioRender.com/21m8lxk. Source data are available online for this figure.

To study how cancer cells adapt to glycolytic inhibition, we investigated the effects of 2-DG treatment on proliferation and metabolism in lung cancer cells in vitro, cultured in medium mimicking serum glucose levels (7 mM). 2-DG treatment significantly decreased the proliferation rate of the lung cancer cell lines A549 and H23 (Fig. 1A) and reduced the growth of H23 lung cancer spheroids (Fig. 1B). These inhibitory effects could effectively be reached at 1 mM 2-DG, which is in the range of peak plasma concentrations measured in clinical trials (Raez et al, 2013). 2-DG treatment reduced total levels of glycolytic intermediates (Appendix Fig. S1A) and decreased labeling from ^13^C_6_-glucose (Appendix Fig. S1B; labeling scheme in Appendix Fig. S1C) in H23 cells. We also observed reduced label transfer from ^13^C_6_-glucose to the tricarboxylic acid (TCA) cycle, as indicated by decreased citrate, succinate and malate M + 2 (Appendix Fig. S1B), supporting the suppression of glycolytic metabolism in vitro by 2-DG.

To investigate the effects of glycolysis inhibition in vivo, we subcutaneously injected A549 cells into immunocompromised mice. Once tumors reached ~65 mm^3^, mice were treated with 2-DG (500 mg/kg intraperitoneally, 3×/week), or saline control. This dosage of 2-DG has been frequently used in mice and was effective in different tumor models, either alone or in combination with other agents (Maschek et al, 2004; Boutrid et al, 2008; Zeng et al, 2016; Goodwin et al, 2017). In agreement with previous reports (Zeng et al, 2016; Goodwin et al, 2017), 2-DG treatment had no effect on the growth of A549 tumors in vivo (Fig. 1C), in stark contrast to A549 cells in culture. This led us to hypothesize that there might be additional nutrients available in vivo, which are not present in cell culture and bypass the constraints of 2-DG treatment, such as alternative sugars. While physiological cell culture media such as human plasma-like medium (HPLM) (Cantor et al, 2017) and Plasmax (Vande Voorde et al, 2019) more closely mimic physiological conditions by matching nutrient abundances in plasma, mannose is absent from these formulations despite being present at 40–80 µM in human serum (Panneerselvam et al, 1997) (Fig. 1D).

When we treated A549 cells with 2-DG, we observed transcriptional changes in mannose-related genes (Fig. 1E,F). Phosphomannomutase 2 (*PMM2*), which mediates a key step in mannose activation for glycosylation, was downregulated upon 2-DG treatment. In contrast, mannose-6 phosphate isomerase (*MPI*), which catalyzes the interconversion of fructose-6-phosphate and mannose-6-phosphate, and glutamine-fructose-6-phosphate transaminase 1 (*GFPT1*), which is involved in glycosylation and the hexosamine pathway, were significantly upregulated (Fig. 1E). A similar trend was observed for PMM2 and MPI in H23 cells, and GFPT1 was significantly upregulated (Appendix Fig. S1D). When we assessed the expression of those genes in the publicly available GDC TCGA LUAD dataset (https://www.cancer.gov/tcga), we found that all three genes were significantly overexpressed in lung adenocarcinoma (LUAD) compared to non-involved lung tissue (Fig. 1G). The deregulation of genes involved in the early steps of mannose metabolism suggests that it might represent an adaptive mechanism in 2-DG treated lung cancer cells.

To test if mannose might be a limiting factor for the efficacy of 2-DG, we added a nearly physiological concentration of 100 µM mannose to the cultured cells. This was sufficient to partially restore the proliferation rate of A549 (Fig. 1H) and H23 cells (Appendix Fig. S1E) treated with 2-DG. Notably, mannose also partially rescued A549 cell proliferation under glucose deprivation (0.2 or 0 mM glucose), although the effect was significant only with complete glucose withdrawal (Fig. 1I). These data show that physiological levels of mannose support proliferation of lung cancer cells when glycolysis or glucose availability is limited.

### Lung cancer cells increase mannose utilization for glycosylation precursors upon 2-DG treatment or glucose starvation

**Figure 2:**
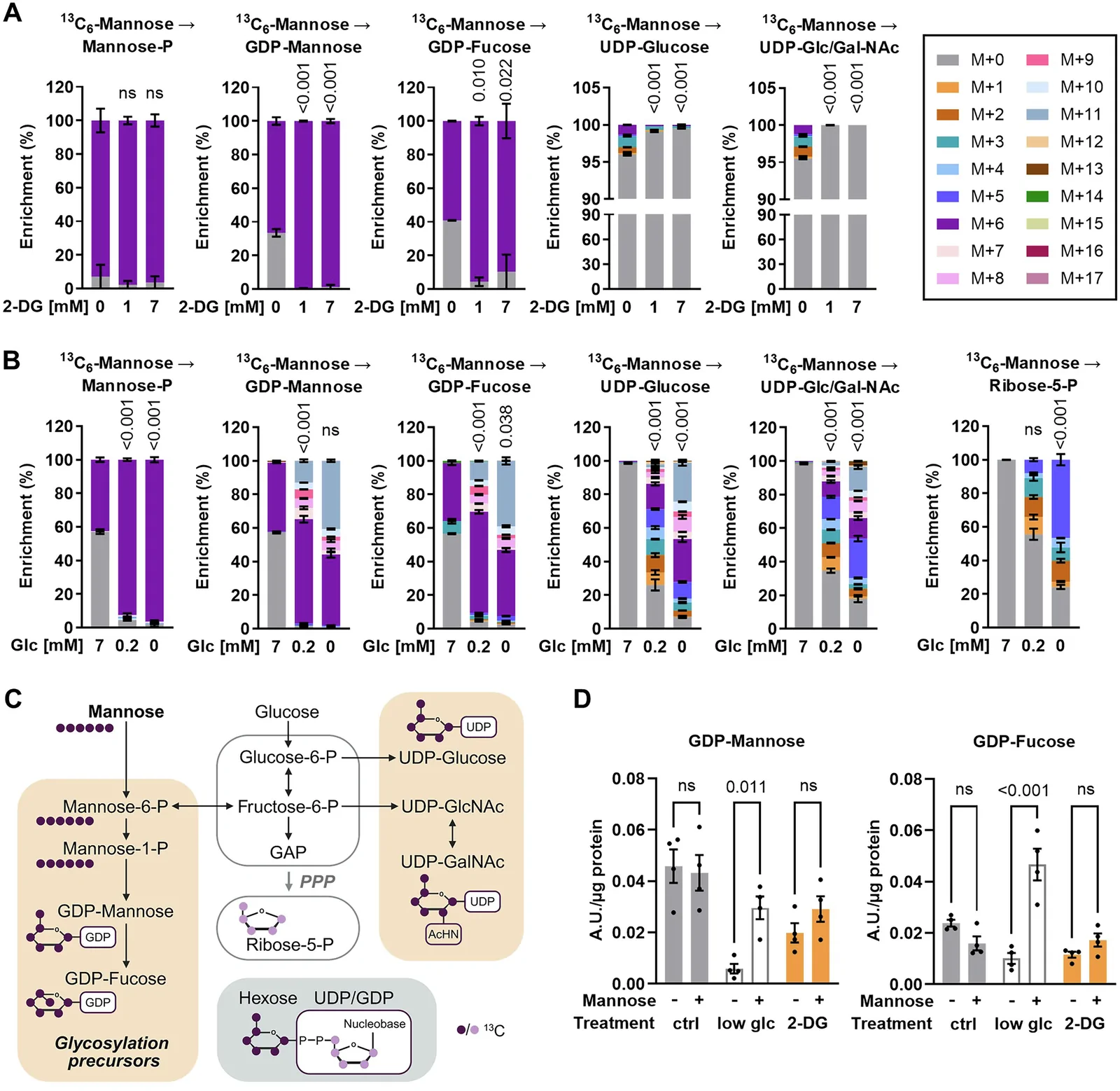
Lung cancer cells increase mannose use for glycosylation upon glycolytic stress. (**A**) Enrichment (%) of all isotopologues in metabolites derived from ^13^C_6_-mannose in A549 cells treated with 2-deoxyglucose (2-DG) or (**B**) glucose (glc) starvation media. Cells were treated with their respective media and 100 µM ^13^C_6_-mannose for 24 h and analyzed by liquid chromatography-mass spectrometry (LC–MS) (*n* = 3). Different LC–MS platforms were used for (**A**, **B**). Mannose-6-phosphate/mannose-1-phosphate is depicted as mannose-P and UDP-GlcNAc/UDP-GalNAc as UDP-Glc/Gal-NAc (not distinguishable with our LC–MS methods). (**C**) Metabolic downstream pathways of ^13^C_6_-mannose. GDP guanosine diphosphate, UDP uridine diphosphate, UDP-GlcNAc UDP-N-acetylglucosamine, UDP-GalNAc UDP-N-acetylgalactosamine, GAP glyceraldehyde 3-phosphate, PPP pentose phosphate pathway. (**D**) Total metabolite abundance in A549 cells treated with 2-DG (1 mM) or low glucose (0.2 mM) media +/− 100 µM mannose for 24 h (*n* = 4). Data are shown as mean +/− SEM from biological replicates (*n*). Statistical significance was determined by (**A**, **B**) one-way ANOVA with Dunnett’s post-hoc analysis versus control condition of the M + 6 (mannose-P and nucleotide-sugars) or M + 5 (ribose-5-P) labeled isotopologue, or (**D**) two-way ANOVA with Šidák’s multiple comparison test. *P* values are provided in the graphs; ns, not significant. Illustration in (**C**) was created in BioRender. Haitzmann, T. (2026) https://BioRender.com/tfokq40. Source data are available online for this figure.

To better understand how mannose restores the proliferation of lung cancer cells under metabolic stress, we assessed the contribution of mannose to different metabolic pathways by stable isotope tracing. We first investigated mannose incorporation into glycosylation precursors. In A549 cells, ^13^C_6_-mannose contributed to ~40–60% of GDP-mannose and GDP-fucose pools in control conditions, and we found a significant increase in enrichment upon treatment with 2-DG (Fig. 2A) or glucose starvation media (Fig. 2B). The metabolic pathway for the generation of GDP-mannose and GDP-fucose, important building blocks needed for glycan biosynthesis, from mannose is depicted in Fig. 2C. In 2-DG treated cells, the predominant isotopologue of GDP-mannose and GDP-fucose was M + 6, suggesting a labeling of the hexose (mannose/fucose) residue. Other activated sugars, such as UDP-glucose, UDP-N-acetylglucosamine (UDP-GlcNAc) and UDP-N-acetylgalactosamine (UDP-GalNAc), potentially can be generated from mannose after conversion to fructose-6-phosphate via MPI (Fig. 2C). However, mannose contribution to UDP-glucose or UDP-GlcNAc/UDP-GalNAc, was generally low and even decreased under 2-DG treatment (Fig. 2A). Metabolite abundances were mostly unchanged under 2-DG treatment in the presence of mannose, except for a significant decrease of UDP-GlcNAc/UDP-GalNAc, and a partial increase of mannose-phosphate under low dose 2-DG treatment (Appendix Fig. S2A).

Under treatment with low (0.2 mM) or glucose-free media, mannose showed enhanced conversion to GDP-mannose and GDP-fucose, as well as UDP-glucose and UDP-GlcNAc/UDP-GalNAc (Fig. 2B). However, increased M + 11 fractions suggested that mannose was also converted to the ribose moiety of the respective nucleotide (UDP/GDP, Fig. 2C). Ribose-5-phosphate (ribose-5-P) can be generated from mannose after conversion to fructose-6-phosphate via MPI (Fig. 2C). In fact, M + 5 labeled ribose-5-P was found under low (0.2 mM) and no glucose treatment (Fig. 2B), showing the conversion of mannose to upper glycolysis and the pentose phosphate pathway (PPP). Glucose starvation reduced the overall total abundance of most nucleotide sugars and ribose-5-P in A549 cells, while GDP-mannose was unchanged and GDP-fucose levels were significantly enhanced (Appendix Fig. S2B).

To investigate the impact of mannose on metabolite pools, we treated the cells with or without exogenous mannose. We found a decrease of GDP-mannose and GDP-fucose levels in glucose starved cells, which was reversed by exogenous mannose (Fig. 2D). A similar trend was observed in 2-DG treated cells (Fig. 2D). This is in line with the significant contribution of mannose to activated mannose and fucose. Total abundances of UDP-GlcNAc/UDP-GalNAc or UDP-glucose were decreased in 2-DG treated cells in the presence of mannose (Appendix Fig. S2C). Ribose-5-P and lower glycolytic intermediate pools were unchanged by exogenous mannose under 2-DG treatment or glucose deprivation (Appendix Fig. S2C). Together, these data show that mannose contributes to a large proportion of GDP-mannose and GDP-fucose in lung cancer cells in vitro, which is further enhanced by 2-DG treatment or glucose starvation.

### Mannose contributes to the glycolysis pathway when glucose is limited

**Figure 3:**
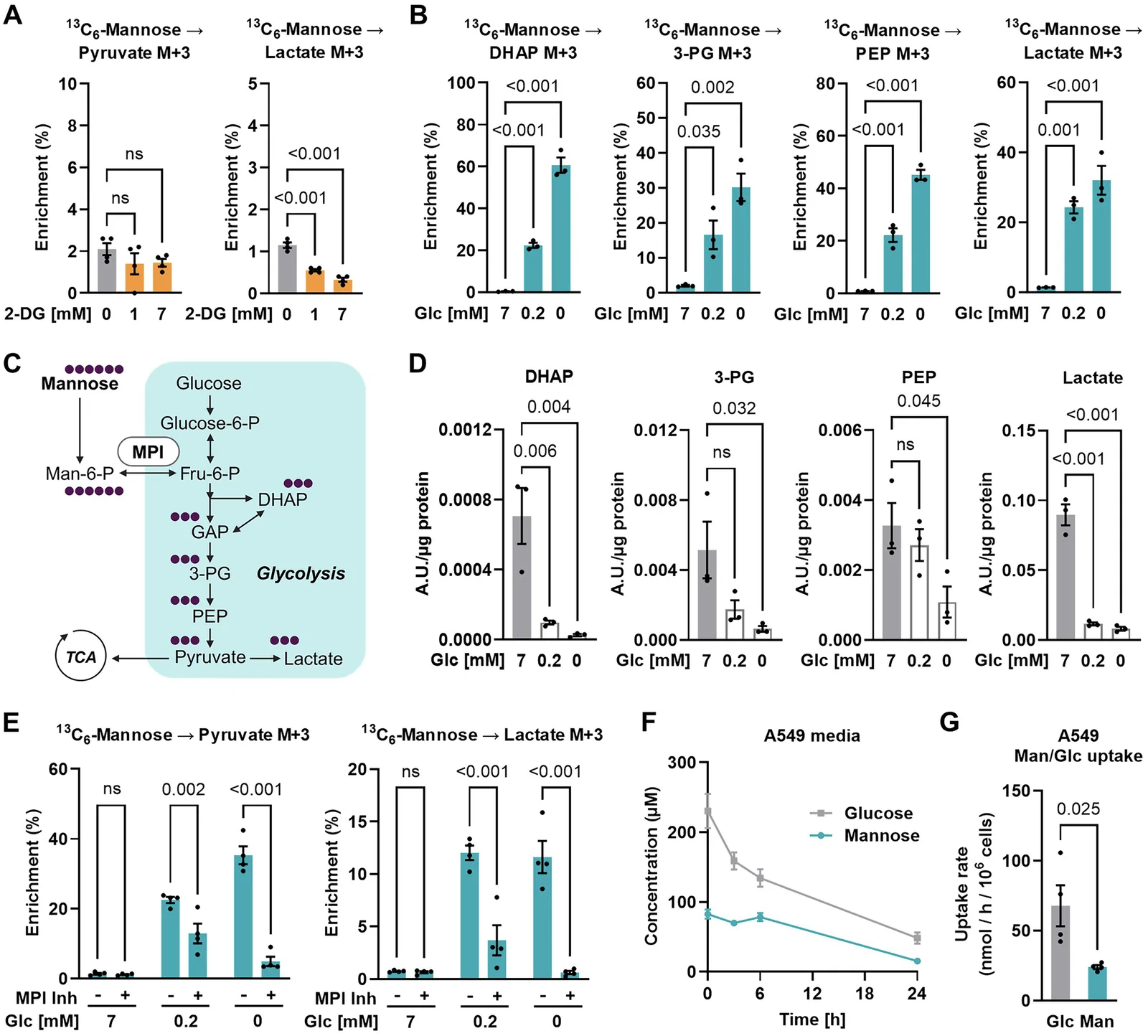
Glucose starved lung cancer cells use mannose to fuel the glycolysis pathway. (**A**) Enrichment (%) of fully ^13^C-labeled pyruvate and lactate (M + 3) in A549 cells treated with 2-deoxyglucose (2-DG) and 100 µM ^13^C_6_-mannose for 24 h (*n* = *4*), measured by gas chromatography-mass spectrometry (GC-MS). (**B**) Enrichment (%) of fully ^13^C-labeled glycolytic metabolites (M + 3) in A549 cells treated with glucose (glc) starvation media and 100 µM ^13^C_6_-mannose for 24 h, analyzed by liquid chromatography-mass spectrometry (LC–MS) (*n* = 3). (**C**) Metabolic downstream pathway of ^13^C_6_-mannose towards glycolysis. Man-6-P mannose-6-phosphate, Fru-6-P fructose-6-phosphate, MPI mannose-6-phosphate isomerase, DHAP dihydroxyacetone phosphate, GAP glyceraldehyde 3-phosphate, 3-PG 3-phosphoglycerate, PEP phosphoenolpyruvate, TCA tricarboxylic acid cycle. (**D**) Total abundance of glycolytic metabolites in A549 cells upon glucose starvation and treatment with 100 µM ^13^C_6_-mannose for 24 h, analyzed by LC–MS (*n* = 3). (**E**) A549 cells were treated with glucose starvation media +/− 50 µM of the MPI inhibitor MLS0315771 or DMSO (control) and 100 µM ^13^C_6_-mannose for 24 h (*n* = 4). Label enrichment was measured by GC-MS analysis. (**F**) Time course of glucose and mannose concentration in A549 media, analyzed by GC-MS (*n* = 4). Quantitative analysis was done by one-point-calibration with ^13^C_6_-standards spiked into the sample matrix. (**G**) Uptake rates of glucose and mannose in A549 cells calculated as nmol/h/10^6^ cells (*n* = 4). Data are shown as mean +/− SEM from biological replicates (*n*). Statistical significance was determined by (**A**, **B**, **D**) one-way ANOVA with Dunnett’s post-hoc analysis, (**E**) two-way ANOVA with Šidák’s multiple comparison test or (**G**) unpaired Student’s *t* test. *P* values are provided in the graphs; ns, not significant. Illustration in (**C**) was created in BioRender. Haitzmann, T. (2026) https://BioRender.com/e4o5xhm. Source data are available online for this figure.

Next, we investigated mannose shuttling towards glycolysis using ^13^C_6_-mannose tracing. The enrichment of fully ^13^C-labeled pyruvate and lactate (M + 3) was relatively low at baseline conditions or under 2-DG treatment (Fig. 3A; Appendix Fig. S3A,B). However, mannose contribution to the glycolytic intermediates dihydroxyacetone phosphate (DHAP), 3-phosphoglycerate (3-PG), phosphoenolpyruvate (PEP) and lactate was significantly increased under glucose deprivation (Fig. 3B,C; Appendix Fig. S3C), as shown by enhanced labeled fractions. Of note, total pool sizes of glycolytic intermediates were lower in glucose-starved cells, especially at 0 mM glucose (Fig. 3D). Thus, enhanced labeling may potentially result from smaller pool sizes due to a reduced contribution from glucose. Formal flux analysis would be needed to verify an enhanced glycolysis fueling by mannose under glucose starvation. Since MPI connects mannose to glycolysis by converting mannose-6-phosphate to fructose-6-phosphate (Fig. 3C), we confirmed its involvement using the MPI inhibitor MLS0315771. Indeed, inhibition of MPI significantly reduced the contribution of mannose to central carbon metabolism in glucose-starved A549 (Fig. 3E) and H23 cells (Appendix Fig. S3D).

When we assessed glucose and mannose uptake in cells treated simultaneously with mannose (100 µM) and glucose (at low glucose conditions, 0.2 mM) we found that mannose uptake-rates are roughly 1/3 of glucose uptake-rates (Fig. 3F,G). This suggests that glucose is still the preferred nutrient but mannose is a contributing source as well. Collectively, these data show that at nearly physiological levels, mannose is widely used by lung cancer cells to generate precursors for glycosylation, and can contribute to glycolytic intermediates when glucose is limiting.

### Stable isotope tracing shows mannose utilization by lung tumors in vivo

**Figure 4:**
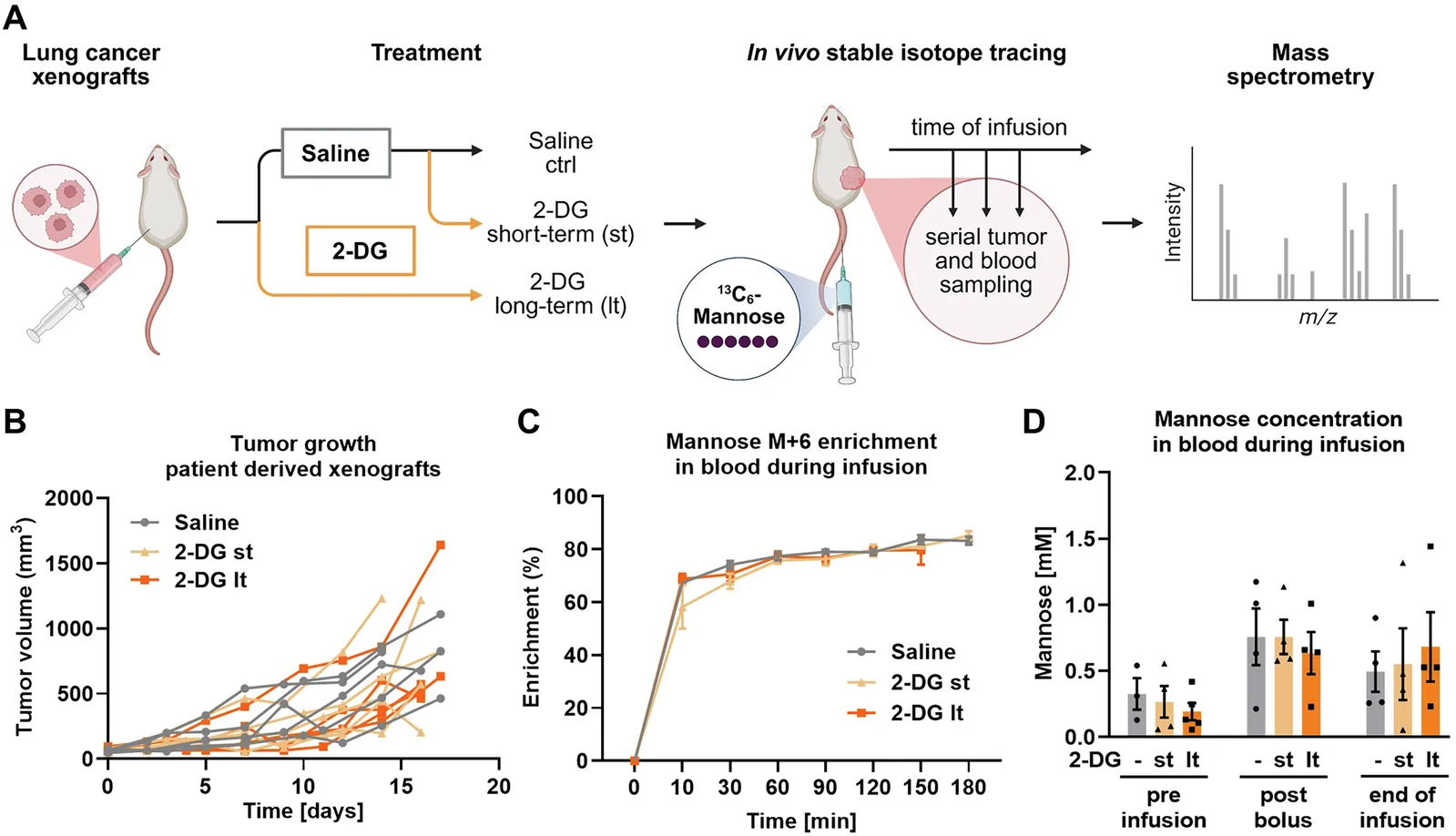
^13^C-mannose tracing in vivo in lung cancer xenografts. (**A**) Schematic of the workflow for 2-deoxyglucose (2-DG) treatment and in vivo stable isotope tracing with ^13^C_6_-mannose in lung cancer mouse xenografts. (**B**) Subcutaneous tumor growth of patient-derived lung cancer xenografts (mx148) treated by intraperitoneal injection with saline (control), long-term (lt) 2-DG (500 mg/kg 2-DG 3x/week for 2 weeks) or short-term (st) 2-DG (single dose of 500 mg/kg 2-DG right before infusion). Tumor volume was calculated as W^2^ × L × 0.52. Each line represents the growth pattern of an individual tumor. (**C**) Enrichment of fully labeled ^13^C-mannose (denoted as M + 6) in the blood of 2-DG treated mouse xenografts over the time-course of infusion, applied as a bolus followed by continuous perfusion (*n* = 2–5; single data points provided in source data; lower sample size in later timepoints due to early death during infusion), measured by gas chromatography-mass spectrometry (GC-MS). (**D**) Total mannose concentration (mM) in the blood of mouse xenografts before ^13^C_6_-mannose infusion, 10 min after the bolus injection and at the end of infusion. The concentration of mannose was quantified by measuring a standard curve of ^13^C_6_-mannose spiked into the sample matrix. (*n* = 3–5*;* single data points provided in source data; lower sample size resulted from limited material availability). (**C**, **D**) Data is shown as mean +/− SEM of biological replicates (*n*). Illustration in (**A**) was created in BioRender. Haitzmann, T. (2026) https://BioRender.com/2wvj30w. Source data are available online for this figure.

To investigate mannose utilization by lung cancer in vivo, we performed stable isotope tracing with uniformly labeled ^13^C-mannose in mouse xenografts of A549 cells and mx148, a NSCLC patient-derived xenograft (PDX) (Cai et al, 2025). To assess acute vs. adaptive changes in response to glycolytic inhibition, mice bearing mx148 tumors were divided into three treatment groups: saline control, long-term (lt) 2-DG (500 mg/kg 2-DG, 3×/week for 2 weeks) and short-term (st) 2-DG (single dose of 500 mg/kg 2-DG, ~1 h before infusion) (Fig. 4A). Similar to cell line xenografts (Fig. 1C), treatment with 2-DG did not affect subcutaneous growth of PDX tumors (Fig. 4B), and did not alter the body weight of mice (Appendix Fig. S4A). Subsequently, ^13^C_6_-mannose was administered as a primed-continuous infusion (0.083 mg/g bodyweight bolus, followed by 0.0017 mg/g bodyweight per minute infusion), which achieved a constant label enrichment of ~70–80% in the blood (Fig. 4C). Using this protocol, the mean elevation of total mannose levels was 3.4-fold (range 2.2–4.9) after the bolus and 1.5-fold (range 0.8–2.0) at the end of infusion compared to baseline in saline control mice (Fig. 4D). Labeled mannose was found in the PDX tumors, liver, and lung irrespective of 2-DG treatment (Appendix Fig. S4B). We confirmed that mannose was not systemically converted to glucose at considerable rates, as blood levels of glucose M + 6 were negligible (Appendix Fig. S4C).

**Figure 5:**
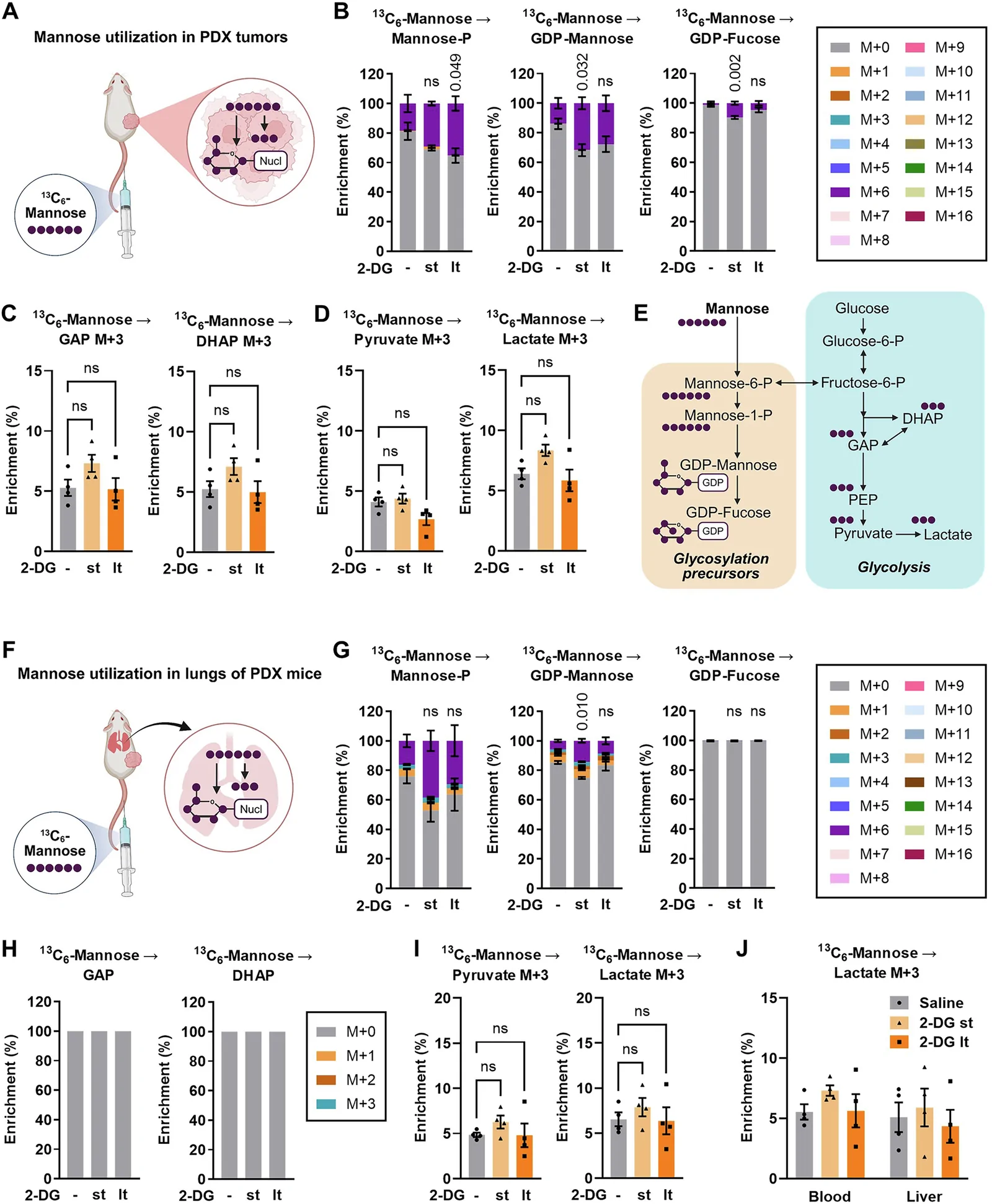
Mannose utilization in vivo in tumors and lungs of PDX mice. (**A**) In vivo stable isotope tracing with ^13^C_6_-mannose in patient-derived mouse xenografts (PDX) and enrichment of labeled metabolites in the tumor. Mice carrying patient-derived xenografts were either treated with 2-deoxyglucose (2-DG; long term, lt; short term, st) or saline and infused with ^13^C_6_-mannose. (**B**) Enrichment (%) of all metabolite isotopologues analyzed by liquid chromatography-mass spectrometry (LC–MS) in PDX tumors at the end of infusion (*n* = 4). Mannose-6-phosphate/mannose-1-phosphate is depicted as mannose-P (not distinguishable with our LC–MS method). (**C**, **D**) Enrichment (%) of fully ^13^C-labeled glycolytic intermediates (denoted as M + 3) in ^13^C_6_-mannose infused PDX tumors at the end of infusion (*n* = 4) measured by (**C**) LC–MS or (**D**) gas chromatography-mass spectrometry (GC-MS). (**E**) Metabolic downstream pathways of ^13^C_6_-mannose. DHAP dihydroxyacetone phosphate, GAP glyceraldehyde 3-phosphate, PEP phosphoenolpyruvate. GDP guanosine diphosphate. (**F**) In vivo stable isotope tracing with ^13^C_6_-mannose in PDX mice and enrichment of labeled metabolites in the lung. (**G**) Enrichment (%) of all metabolite isotopologues in lungs of ^13^C_6_-mannose infused PDX mice, analyzed by liquid chromatography-mass spectrometry (LC–MS) (*n* = 4). (**H**) Enrichment (%) of all isotopologues or (**I**) fully ^13^C-labeled glycolytic intermediates (denoted as M + 3) in lungs of ^13^C_6_-mannose infused PDX mice measured by LC–MS (*n* = 4). (**J**) Enrichment (%) of fully ^13^C-labeled lactate (denoted as M + 3) in blood and liver of ^13^C_6_-mannose infused PDX mice (*n* = 4) measured by gas chromatography-mass spectrometry (GC-MS) (*n* = 4). Data is shown as mean +/− SEM of biological replicates (*n*). (**B**–**D**, **G**, **I**) Statistical significance was determined by one-way ANOVA with Dunnett’s post-hoc analysis versus control condition of the M + 6 (mannose-P and nucleotide-sugars) or M + 3 (glycolytic intermediates) labeled isotopologue. *P* values are provided in the graphs; ns, not significant. Illustration in (**A**, **E**, **F**) was created in BioRender. Haitzmann, T. (2026) https://BioRender.com/eshf2vh. Source data are available online for this figure.

To investigate the dynamic enrichment of ^13^C_6_-mannose to different downstream pathways, we collected serial tumor samples throughout the time of infusion (Figs. 4A and 5A). In untreated animals, ^13^C_6_-mannose contributed to a considerable proportion (~ 15–20%) of mannose-phosphate (mannose-6-phosphate/mannose-1-phosphate) and GDP-mannose in the tumors (Fig. 5B). In PDX tumors of mice treated with 2-DG, the conversion of mannose to GDP-mannose and GDP-fucose was significantly enhanced, if applied short-term (st) before tracing (Fig. 5B). Total abundances of mannose-phosphate and the nucleotide sugars remained unchanged across treatment groups (Appendix Fig. S5A). We also observed a contribution of mannose to UDP-glucose and UDP-GlcNAc/UDP-GalNAc, yet at lower levels, and the fractions were not elevated under 2-DG treatment (Appendix Fig. S5B). These data show that systemic mannose is taken up by lung cancer xenograft tumors and contributes to glycosylation pathways, especially to activated mannose and fucose.

When we investigated mannose contribution to glycolytic intermediates, we observed enrichment of glyceraldehyde-3-phosphate (GAP), DHAP and lactate ranging from 5 to 10% (Fig. 5C,D; Appendix Fig. S5C–F, labeling scheme in Fig. 5E). However, 2-DG treatment did not significantly influence mannose contribution to the glycolysis pathway in the tumors. The increase of lactate M + 3 in the tumors over the time of infusion can be seen in Appendix Fig. S5G (total levels Appendix Fig. S5H, A549 xenograft tumors Appendix Fig. S5I,J). Collectively, our in vivo labeling approach shows that mannose is an important precursor for glycosylation in lung cancer xenograft tumors, especially when treated with 2-DG, and is also incorporated into the glycolysis pathway.

We also examined labeling from ^13^C_6_-mannose in downstream metabolites in the lungs of PDX-bearing mice (Fig. 5F). While mannose contribution to mannose-phosphate and GDP-mannose was present in both tumors and lungs, mannose conversion to GDP-fucose was completely absent in the lungs (Fig. 5G). Interestingly, labeled lactate and pyruvate were detected in the lungs, but no labeling in the upstream glycolytic intermediates GAP and DHAP was observed (Fig. 5H,I). The total abundance of those metabolites was unchanged across different treatment groups (Appendix Fig. S6A,B). Of note, similar enrichment of labeled lactate was also found in the blood and liver of mice infused with ^13^C_6_-mannose (Fig. 5J; Appendix Fig. S6C). Potentially, systemic labeled lactate from peripheral tissues such as the liver, which increased in the blood during infusion (Appendix Fig. S6D) may give rise to labeled lactate and pyruvate (after conversion by LDH) in the lungs (isotopologue distribution in Appendix Fig. S6E,F). Together, these data indicate greater mannose routing into glycolytic and glycosylation intermediates by the tumors compared to normal lungs.

### Mannose supports lung cancer cells through context-specific contributions to glycolysis and glycosylation

**Figure 6:**
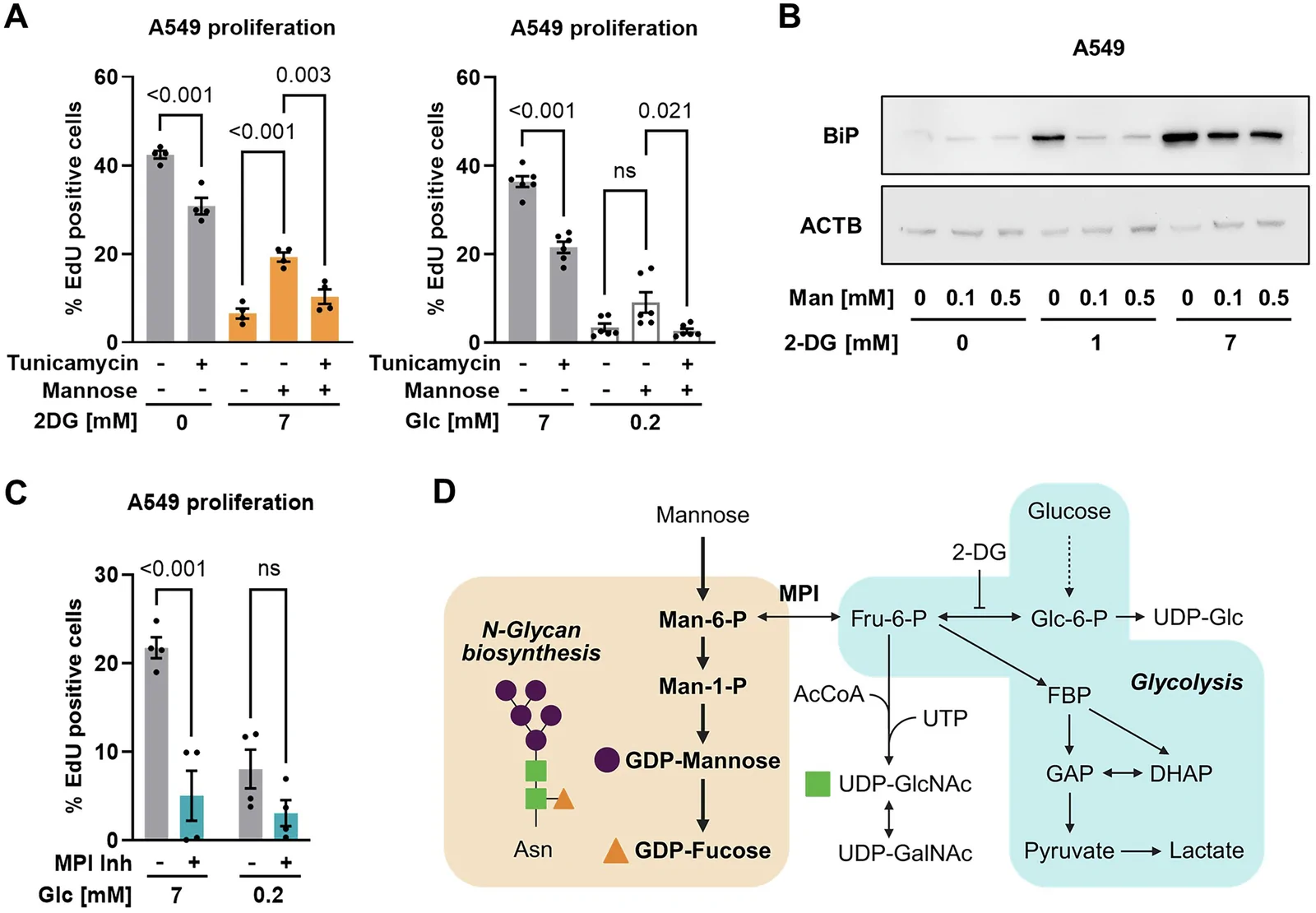
Importance of mannose use for glycosylation pathways or glycolysis. (**A**) A549 proliferation (% EdU positive cells) after treatment with 2-deoxyglucose (2-DG) or low glucose media in combination with 0.2 µg/mL tunicamycin or DMSO (control) +/− 100 µM mannose for 48 h (*n* = 4 for 2-DG treated, *n* = 6 for glucose starved A549 cells). (**B**) Protein expression of binding immunoglobulin protein (BiP, marker for endoplasmic reticulum (ER) stress) and beta-actin (ACTB, loading control) in A549 cells treated with 2-DG and mannose for 48 h. Representative Western blots are shown. A quantification is provided in Appendix Fig. S7B. (**C**) Proliferation rate (% EdU positive cells) of A549 cells treated with glucose (glc) starvation media in combination with 50 µM of the MPI inhibitor MLS0315771 or DMSO (control) and 100 µM mannose for 24 h (*n* = 4). (**D**) Model of mannose-fueled metabolic pathways. Asn asparagine, Man-6-P mannose-6-phosphate, Man-1-P mannose-1-phosphate, MPI mannose-6-phosphate isomerase, Fru-6-P fructose-6-phosphate, GDP guanosine diphosphate, UDP uridine diphosphate, UDP-GlcNAc UDP-N-acetylglucosamine, UDP-GalNAc UDP-N-acetylgalactosamine, Glc-6-P glucose-6-phosphate, UDP-Glc UDP-glucose, FBP fructose-1,6-bisphosphate, GAP glyceraldehyde-3-phosphate, DHAP dihydroxyacetone phosphate. Data is shown as mean +/− SEM of biological replicates (*n*). Statistical significance was determined by (**A**) one-way ANOVA with Tukey’s post-hoc analysis or (**C**) two-way ANOVA with Šidák’s multiple comparison test; *P* values are provided in the graphs; ns, not significant. Illustration in (**D**) was created in BioRender. Haitzmann, T. (2026) https://BioRender.com/ykw5728. Source data are available online for this figure.

To decipher whether mannose supports survival through glycosylation pathways or its shuttling towards glycolysis, we combined glucose starvation or 2-DG treatment with inhibitors for the respective pathways. In order to assess whether the rescue effect of mannose on proliferation of lung cancer cells can be attributed to mannose-derived glycosylation, we treated cells with tunicamycin, an inhibitor of N-linked glycosylation. Tunicamycin treatment alone reduced proliferation, and moreover, reversed the rescue effect of mannose in 2-DG treated lung cancer cells (Fig. 6A, left graph). Similarly, exogenous mannose rescued proliferation of cells treated with low glucose (0.2 mM) media (*P* = 0.05), which was likewise abolished by tunicamycin (Fig. 6A, right graph). Additionally, we analyzed the expression of binding immunoglobulin protein (BiP), a marker for endoplasmic reticulum (ER) stress which can emerge from glycosylation interference, in lung cancer cells treated with 2-DG and mannose. 2-DG treatment induced BiP accumulation, which was clearly diminished by mannose addition (Fig. 6B; Appendix Fig. S7A,B).

As previously observed, mannose utilization for glycolysis under glucose starvation was blocked by MPI inhibition with MLS0315771 in lung cancer cells (Fig. 3E; Appendix Fig. S3D). Inhibition of MPI significantly decreased A549 proliferation in high (7 mM) glucose conditions (Fig. 6C), possibly by inhibiting MPI activity in the direction of mannose-phosphate-formation. In low glucose conditions, a trend for decreased proliferation by MPI inhibition was observed (Fig. 6C). This highlights the importance of the MPI-mediated exchange between mannose utilization pathways and suggests that mannose fueling of glycolysis may play a role under glucose starvation (Fig. 6D). Taken together, our data suggest that the contribution of mannose to glycosylation is an important downstream fate of mannose. However, during glucose limitation, mannose can enter glycolysis and support branching metabolism through MPI-dependent pathways.

## Discussion

This study highlights mannose as an important substrate for lung cancer cells, especially under metabolic stress. We show for the first time that ^13^C mannose is incorporated into precursors for N-glycan biosynthesis in tumors in vivo, which is increased under treatment with 2-DG. In addition, ^13^C mannose contributed to glycolysis in lung tumors in vivo and glucose-deprived cells in vitro. Accordingly, we found that physiological levels of mannose partially rescue proliferation in lung cancer cells treated with the glycolysis inhibitor 2-DG or glucose starvation.

Glycosylation is one of the most common post-translational modifications of proteins and is crucial for their proper function (He et al, 2024). In the context of cancer, changes in glycosylation have been shown to influence several key cancer hallmarks, including tumor progression, metastasis and immune evasion (Reily et al, 2019). Cancer cells frequently show different alterations of glycosylation, including increased N-glycan branching and altered core fucosylation (Reily et al, 2019). Interestingly, enhanced levels and alterations in fucosylation have been described in lung cancer (Ruhaak et al, 2015) and are investigated as potential biomarkers or therapeutic targets (Jia et al, 2018). Interference with glycosylation, such as inhibition of MPI, the initial enzyme mediating mannose-6-phosphate synthesis from glucose, has recently been shown to increase therapy sensitivity in leukemia models (Woodley et al, 2023) and glioma cells (Cazet et al, 2014).

Mannose has been shown to be efficiently used for N-glycan synthesis in fibroblasts (Ichikawa et al, 2014). Our data show that in A549 cells, GDP-linked (activated) mannose and fucose are mainly derived from ^13^C-mannose even in the presence of glucose, and that the enrichment of ^13^C-mannose in these glycosylation precursors reached almost 100% in glucose starved or 2-DG treated cells. We also found increased labeling of GDP-mannose and GDP-fucose in vivo in 2-DG treated mouse xenograft tumors infused with ^13^C_6_-mannose. The increased use of mannose for glycosylation pathways in lung cancer cells in vitro and tumors in vivo upon treatment with 2-DG suggests that mannose might contribute to the limited efficacy of this intervention. Other activated sugars, such as UDP-GlcNAc/UDP-GalNAc or UDP-glucose, were only marginally labeled in vivo and control conditions in vitro. These data suggest that mannose preferentially supports mannosylation and fucosylation, rather than globally elevating all glycosylation precursors. In line with this, exogenous mannose restored the levels of GDP-mannose and GDP-fucose in glucose-restricted conditions in vitro. Unfortunately, directly blocking mannose uptake to determine its requirement for tumor metabolism in vivo was not feasible, as mannose is transported through hexose transporters that also mediate glucose uptake. Normal lungs of ^13^C_6_-mannose-infused mice showed an uptake and conversion of mannose to mannose-phosphate (mannose-1-P/mannose-6-P) and GDP-mannose, whereas GDP-fucose was not labeled. This suggests that the use of mannose for fucosylation may be a unique feature of the NSCLC cells in comparison to the normal lung. As a limitation, we did not investigate glycan structures, mannose incorporation into glycoproteins, or the effects of these processes on ER stress in vivo.

We show that mannose is not only used for glycosylation, but also contributes to the glycolysis pathway in tumors in vivo. Cancers often experience low glucose availability in their microenvironment (Gullino et al, 1964; Sullivan et al, 2019), which may lead to enhanced dependency on alternative nutrients. Interestingly, we found a considerable fraction of mannose-derived pyruvate and lactate in tumors in vivo and in glucose-deprived lung cancer cells in vitro, dependent on MPI acting in the direction of fructose-6-phosphate formation. Mannose has been previously shown to contribute to glycolytic intermediates (Ichikawa et al, 2014; Gonzalez et al, 2018; Luciano-Mateo et al, 2026), especially in the absence of glucose or in the presence of high concentrations of mannose. While it may be expected that cells utilize this pathway when glucose is missing, we show that the mannose-to-glycolysis shuttling also occurs in the presence of low glucose (0.2 mM) levels. We additionally found a shuttling of mannose carbons towards ribose-5-phosphate via the non-oxidative PPP in low glucose conditions, in line with a recent publication (Luciano-Mateo et al, 2026). Of note, a glucose concentration of 0.2 mM was chosen for in vitro experiments, consistent with prior lung cancer studies modeling reduced glucose availability (Haitzmann et al, 2024). However, similar labeling patterns of glycolytic intermediates in vivo suggest that mannose may be used for glycolysis in vivo when glucose availability is limited.

Our in vitro data showed that the contribution of mannose to glycolysis was clearly increased under low glucose conditions. This was partly associated with lower abundance of the respective glycolytic intermediates. Whether low glucose conditions in fact lead to an enhanced flux from mannose-6-P to glycolysis or whether the increased contribution is caused by smaller pool sizes cannot be fully answered based on our data. Formal flux analysis and dynamic labeling strategies would be required to quantify the different pathway activities. However, our study provides initial data that can be incorporated in flux models in future studies. In our in vivo labeling approach, we likewise found labeled glycolytic intermediates DHAP and GAP, pyruvate and lactate in the tumors, while labeling in mouse lungs only occurred in the glycolytic end products, pyruvate and lactate. Labeled lactate from ^13^C_6_-mannose was also found in normal liver and blood. Potentially, labeled blood lactate may have contributed to the labeled lactate in the lung, as lactate is avidly taken up by different tissues and converted to pyruvate via lactate dehydrogenase (Hui et al, 2017). As a limitation of the study, subcutaneous tumor models (PDX and A549) were used in immunocompromised mice, which do not fully recapitulate the metabolic microenvironment of lung cancers. Thus, the analysis of mannose utilization in human lung cancers or genetically engineered tumor models would be warranted.

High doses of mannose have been reported to have anti-cancer properties, particularly in cancer cells with low expression of MPI (Gonzalez et al, 2018; Harada et al, 2023). Relatedly, in a study with leukemia cells, the authors found that mannose loading beyond the processing capacity of MPI suppresses leukemia cell proliferation (Saito et al, 2021). Administration of very high levels of mannose has led to decreased tumor growth of cancer cells in vitro and in mouse models in vivo in multiple cancer types (Gonzalez et al, 2018; Jin et al, 2025). Potentially, this may be attributed to suppression of glycolytic enzyme activity by accumulation of mannose-6-phosphate (DeRossi et al, 2006). Interestingly, mannose supplementation enhanced the efficacy of immunotherapy in an ovarian cancer model by altering the gut microbiome (Zhang et al, 2025). While mannose supplementation at high doses may reduce tumor growth, our study positions mannose as a nutrient fueling important biosynthetic pathways.

In summary, our study shows that mannose is an important precursor for glycosylation pathways in lung cancer cells in vivo, and that it may contribute to the resistance of lung cancer to glycolysis inhibition via 2-DG. Limiting mannose uptake and metabolism alongside inhibition of glycolysis might be a promising therapeutic strategy in the future.

## Methods

**Table:** Reagents and tools table

Reagent/resource	Reference or source	Identifier or catalog number
**Experimental models**		
NCI-H23 cells (*H. sapiens*)	American Type Culture Collection	CRL-5800
A549 cells (*H. sapiens*)	Cell Lines Service GmbH	N/A
mx148 cells (*H. sapiens*)	Cai et al (2025)	N/A
NSG mice (*M. musculus*)	Jackson Laboratories	JAX #005557
**Recombinant DNA**		
N/A		
**Antibodies**		
Rabbit anti-BiP (C50B12)	Cell Signaling Technology	#3177
Mouse anti-beta actin (C4)	Santa Cruz Biotechnology	sc-47778
Goat anti-rabbit HRP	Cell Signaling Technology	#7074
Horse anti-mouse HRP	Cell Signaling Technology	#7076
**Oligonucleotides and other sequence-based reagents**		
PCR primers	This study	Method section
**Chemicals, enzymes and other reagents**		
DMEM/F-12, no glutamine	Gibco^TM^, Thermo Fisher Scientific	21331020
RPMI 1640 Medium, no glutamine	Gibco^TM^, Thermo Fisher Scientific	31870074
DMEM, no glucose, no glutamine, no phenol red	Gibco^TM^, Thermo Fisher Scientific	A1443001
SILAC RPMI 1640 Flex Media, no glucose, no phenol red	Gibco^TM^, Thermo Fisher Scientific	A2494201
Fetal Bovine Serum, South American origin	Biowest	S1810
Fetal Bovine Serum, dialyzed, US origin	Gibco^TM^, Thermo Fisher Scientific	26400044
2-Deoxy-D-glucose	Sigma-Aldrich	D8375
2-Deoxy-D-glucose	Thermo Fisher Scientific	111980050
Tunicamycin	Sigma-Aldrich	T7765
MLS0315771	MedChemExpress	HY-112945
Click-iT™ EdU Alexa Fluor™ 488 Flow Cytometry Assay Kit	Invitrogen^TM^, Thermo Fisher Scientific	C10425
peqGOLD Total RNA Kit	VWR	13-6834-02
qScript^TM^ cDNA Synthesis Kit	Quantabio	95047-500
Blue S’Green qPCR Kit Separate ROX	Biozym	331416
BCA Protein Assay Kit	Millipore	71285-3
SuperSignal^TM^ West Pico PLUS Chemiluminescent Substrate	Thermo Fisher Scientific	34580
D-Glucose (U-¹³C₆, 99%)	Cambridge Isotope Laboratories	CLM-1396
D-Mannose (U-¹³C₆, 99%)	Cambridge Isotope Laboratories	CLM-6567
Methoxamine (MOX)	Thermo Fisher Scientific	TS-45950
MTBSTFA + 1% TBDMS	Thermo Fisher Scientific	TS-48927
Matrigel Matrix	Corning	354248
Cultrex Basement Membrane Extract	Bio-Techne	3432-010-01
**Software**		
FlowJo^TM^ (10.10.0)	https://www.flowjo.com/	
CellSens Standard (1.16)	Olympus	
CFX Manager Software (3.1)	Bio-Rad	
Image Lab (6.1)	Bio-Rad	
El-Maven (0.12.0)	Agrawal et al (2019)	
IsoCor (2.2.1)	Millard et al (2019)	
Xcalibur	Thermo Fisher Scientific	
Compound Discoverer 3.3	Thermo Fisher Scientific	
Skyline (25.1.0.237)	Adams et al (2020)	
Freestyle (1.8 SP2)	Thermo Fisher Scientific	
GraphPad Prism (10.4.2)	https://www.graphpad.com/	
**Other**		
CytoFLEX S Flow Cytometer	Beckman Coulter	
Olympus BX-51 microscope	Olympus	
CFX384 Touch Real-Time PCR Detection System	Bio-Rad	
ChemiDoc Touch	Bio-Rad	
Agilent 7890B GC system, Agilent 5977 A Inert MS system	Agilent	
Agilent 7890 GC System, Agilent 5977B Mass Selective Detector	Agilent	
Vanquish Horizon UHPLC system, Orbitrap IQ-X Tribrid mass spectrometer	Thermo Fisher Scientific	
Vanquish system, Orbitrap Eclipse Tribrid mass spectrometer	Thermo Fisher Scientific	

### Cell culture

The following human NSCLC cell lines were used: NCI-H23 (American Type Culture Collection, Manassas, VA, USA), further referred to as H23, and A549 (Cell Lines Service GmbH, Eppelheim, Germany). Cells were grown under standard conditions at 37 °C with 5% CO_2_. A549 cells were cultured in DMEM/F-12, no glutamine (Gibco, Thermo Fisher Scientific, Waltham, MA, USA). H23 cells were grown in RPMI 1640, no glutamine (Gibco). Both culture media were supplemented with 2 mM glutamine (Gibco), 10% fetal bovine serum (FBS, Biowest, Nuaillé, France) and penicillin–streptomycin (Gibco). Authentication of cell lines was done by Short Tandem Repeat (STR) analysis using the PowerPlex 16HS System (Promega, Madison, WI, USA). For in vitro experiments, lung cancer cells were plated in their respective growth media. On the next day, cells were washed two times with PBS and treated with the subsequent treatment media. DMEM, no glucose, no glutamine, no phenol red (Gibco) supplemented with 10% dialyzed fetal bovine serum (Gibco), 7 mM glucose (Sigma-Aldrich, St. Louis, MO, USA), 2 mM glutamine (Sigma-Aldrich) and antibiotics was used for A549 cells; SILAC RPMI 1640 Flex Media, no glucose, no phenol red (Gibco), supplemented with 10% dialyzed fetal bovine serum, 7 mM glucose, 2 mM glutamine, antibiotics, as well as 1.15 mM arginine and 0.27 mM lysine (Sigma-Aldrich) was used for H23 cells. For glucose starvation conditions, concentrations of 0 or 0.2 mM glucose were used. 2-deoxyglucose (2-DG, Sigma-Aldrich or Thermo Fisher Scientific) was supplied as indicated. D-mannose (Sigma-Aldrich) was added in a concentration of 100 or 500 µM as depicted. Tunicamycin (Sigma-Aldrich) and the MPI inhibitor MLS0315771 (MedChemExpress, Monmouth Junction, NJ, USA) were dissolved in DMSO. In experiments with those inhibitors, DMSO was added in the same dilution as a vehicle control. Treatment duration varied across experiments as mentioned in the related paragraphs. Source information on cell lines, culture media and reagents is provided in the Reagents and Tools table.

### Animal studies

All procedures were approved by the University of Chicago’s Institutional Animal Care and Use protocols (ACUP-72678). Healthy, female NOD.Cg-Prkdc^scid^, Il2rg^tm1Wjl^/SzJ (NSG) mice were obtained from Jackson Laboratories, Bar Harbor, ME, USA (JAX #005557). Mice were xenografted at eight to twelve weeks of age. All mice were housed in pathogen-free conditions. Mice were monitored regularly and determined to be healthy by the veterinary staff and the study team. No sample-size calculation was performed. Sample sizes were determined based on standard experimental group sizes to achieve acceptable power. For the generation of mouse xenografts, either patient-derived tumor cells (mx148), a KRAS-mutant pleomorphic NSCLC (Cai et al, 2025) or A549 cells were used. Cells were subcutaneously injected into the right flank of mice, in a 100 µL 1:1 mixture of Hank’s Balanced Salt Solution (HBSS) and Matrigel (354248, Corning, New York, NY, USA) or Cultrex Basement Membrane Extract (3432-010-01, Bio-Techne, Minneapolis, MN, USA). For injection, 2–8 × 10^5^ of the patient-derived mx148 cells and 1 × 10^6^ A549 cells were used. Once the tumors reached a size of ~65 mm^3^, mice were treated with saline or 2-DG (Thermo Fisher Scientific), administered by intraperitoneal injection. Patient-derived xenograft mice were randomized into three treatment groups: saline control (saline 3×/week for 2 weeks), long-term (lt) 2-DG (500 mg/kg 2-DG, 3×/week for 2 weeks) and short-term (st) 2-DG (saline 3×/week for 2 weeks and a single dose of 500 mg/kg 2-DG, ~1 h before infusion). A549 xenografts were divided in two groups: saline and long-term 2-DG treatment. Examiners were not blinded to the treatment, since stable isotope enrichment as the main readout is not observer-dependent. Tumor size and body weight were measured three times a week to track tumor growth and monitor weight changes. Subcutaneous tumors were measured with calipers and the tumor volume (mm^3^) was calculated as W^2^ × L × 0.52.

### Proliferation assay

The proliferation rate of cultured cells was measured by using the Click-iT^TM^ EdU Alexa Fluor^TM^ 488 Flow Cytometry Assay Kit (Invitrogen, Thermo Fisher Scientific) according to the instructions of the manufacturer. Briefly, cells were plated in six-well plates at a density of 2–3 × 10^5^ cells/well. Treatment with the respective media was done for 48 h, except for proliferation assessment upon treatment with the MPI inhibitor MLS0315771, which was done for 24 h. Thereafter, cells were incubated for 1.5 h at 37 °C with 10 µM EdU solution. Subsequently, cells were analyzed by fluorescence-activated cell sorting (FACS) analysis for their fluorescent signal (EdU positive cells) on the CytoFLEX S Flow Cytometer (Beckman Coulter, Brea, CA, US). Gating was performed using the FlowJo Software 10.10.0 (BD Biosciences, San Jose, CA, USA). A representative gating strategy is provided in Appendix Fig. S8.

### Lung cancer spheroids

For generation of spheroids, H23 cells were plated in ultra-low-attachment round bottom 96-well plates (Corning or Thermo Fisher Scientific) at 7500 cells/well, followed by centrifugation for 10 min at 1500 rpm. The media composition for spheroid plating was RPMI 1640 SILAC (Gibco), supplemented with 7 mM glucose, 10% dialyzed fetal bovine serum, 2 mM glutamine, antibiotics, as well as 1.15 mM arginine and 0.27 mM lysine. Treatment of spheroids started three days after plating. The cross-sectional radius *r* was measured on day 0, 4, 7 and 11 after treatment using Olympus BX-51 microscope and cellSens Standard software 1.16 (Olympus, Shinjuku, Tokyo, Japan). The volume *V* of the spheroids was calculated from the cross-sectional radius *r* by the equation *V* = *4/3 x π x r*^*3*^.

### Quantitative real-time PCR (qPCR)

For RNA analysis, 2 × 10^5^ cells/well were plated in six-well plates and treated with the respective media for 24 h. Extraction of RNA was done using the peqGOLD Total RNA Kit (VWR, Radnor, PA, USA) according to the manufacturer’s instructions. An amount of 1000 ng of total RNA was reverse transcribed with the qScript^TM^ cDNA synthesis kit (Quantabio, Beverly, MA, USA). Quantitative real-time PCR (qPCR) was performed on the CFX384 Touch Real-Time PCR Detection System (Bio-Rad, Hercules, CA, USA) using a Biozym Blue S’Green qPCR Kit (Biozym Scientific GmbH, Hessisch Oldendorf, Germany) in a 384-well plate. For the statistical analysis of qPCR results, ΔCt-values were calculated by subtracting Ct of the gene of interest from Ct of the reference gene, human 18S ribosomal RNA (h18S rRNA). The following primers (Eurofins Genomics GmbH, Ebersberg, Germany) were used: PMM2, 5’-CCCCGCGGCAGAAAATTAC-3’ (forward), 5’-CCAAGCCATTTTCTGGAAACACA-3’ (reverse); MPI, 5’-ATGGGTTCCAACAGCGAAGT-3’ (forward), 5’-GAGTCCCCATCCACAACTCTG-3’ (reverse); GFPT1, 5’-ACCGAAGCTCGTGTGTGC-3’ (forward), 5’-CTCGTCTCGTTCGAGGAACAT-3’ (reverse); h18S rRNA, 5’-CTACCACATCCAAGGAAGCA-3’ (forward), 5’-TTTTTCGTCACTACCTCCCCG-3’ (reverse).

### Western blot analysis

Cells were plated in six-well plates at a density of 2 × 10^5^ cells/well and treated for 48 h. Thereafter, protein was collected by lysing the cells on ice in RIPA buffer (Sigma-Aldrich) that was supplemented with protease and phosphatase inhibitors (Thermo Fisher Scientific). Protein content was assessed using the Millipore BCA Protein Assay Kit (Sigma-Aldrich). Separation of samples (10 µg/lane) was performed on 10% sodium dodecyl sulfate-polyacrylamide (SDS-PAGE) gels, followed by transfer to a polyvinylidene difluoride (PVDF) membrane (Bio-Rad). Membranes were then blocked in 5% milk-TBST for 1 h at room temperature and incubated with the following primary antibodies overnight at 4 °C: BiP (C50B12, Rabbit Monoclonal Antibody #3177, Cell Signaling Technology, Danvers, MA, USA) 1:1000 in 5% milk-TBST, beta actin antibody (ACTB, sc-47778, clone C4, Santa Cruz Biotechnology, Dallas, TX, USA) 1:3000 in 5% milk-TBST. On the next day, the membranes were washed and incubated with the respective secondary antibody (1:3000 in TBST) for 1 h at room temperature. Catalogue numbers of antibodies used in this study are provided in the Reagents and Tools table. Detection of chemiluminescence was done on ChemiDoc Touch (Bio-Rad) using SuperSignal^TM^ West Pico PLUS Chemiluminescent Substrate (Thermo Fisher Scientific). The Image Lab Software 6.1 (Bio-Rad) was used to quantify the signal intensity of detected bands. Adjusted values were generated through global background subtraction method and relative expression values were calculated by normalization to the loading control, ACTB.

### Stable isotope tracing in vitro

For glucose tracing experiments, 2 × 10^5^ cells/well were plated in 6-well plates. After a pre-treatment of 24 h, cells were treated again for further 24 h with the respective treatment media including 7 mM ^13^C_6_-glucose (Cambridge Isotope Laboratories, Tewksbury, MA, USA). For ^13^C-mannose tracing experiments, 3 × 10^5^ cells were plated and treated with the respective media including 100 µM ^13^C_6_-mannose (Cambridge Isotope Laboratories) for 24 h. Catalogue numbers of respective ^13^C-tracers are provided in the Reagents and Tools table. Metabolite extraction was performed as follows: cells were quickly rinsed with saline, flash-frozen on liquid nitrogen and scraped on ice with 62.5% methanol containing norvaline (Sigma-Aldrich) as an internal standard. For phase separation, cooled chloroform was added to the samples, followed by centrifugation and transfer of the polar phase. Alternatively, cells were washed with saline and metabolites were extracted by scraping wells with 80% methanol on dry ice. After centrifugation, the supernatant was transferred and myristic-d_27_ acid (Sigma-Aldrich) was added as an internal standard. Subsequent to metabolite extraction, the polar phase/solvent containing the polar metabolites was evaporated by vacuum centrifugation using a SpeedVac (Thermo Fisher Scientific).

### Stable isotope tracing in vivo

In vivo stable isotope tracing was performed as previously described (Faubert et al, 2021). Mice that have been treated with 2-DG or saline were anesthetized with ketamine/xylazine. Subsequently, they were infused with 0.5 g/kg ^13^C_6_-mannose dissolved in 750 µL saline through a 25-gauge catheter that was placed in the lateral tail vein. The administration of the mannose solution was started with a bolus 125 µL/min for 1 min, followed by a continuous flow rate of 2.5 µL/min for up to 3 h. During infusion, blood, and tumor biopsies were collected every 30 min to obtain a time course of the tracer enrichment. Blood samples were collected by retro-orbital blood draw with heparinized capillary tubes (Thermo Fisher Scientific) and chilled on ice. At the end of infusion, mice were euthanized and the rest of the tumor, as well as organs, were collected and frozen in liquid nitrogen. Data showing tumors at end of infusion are from mice infused for 1.5–3 h. Mice with infusion times <1.5 h resulting from premature death were excluded from endpoint analysis. Metabolites from whole blood (~ 10–20 µL) as well as frozen tissue samples (~ 20–50 mg) were extracted in 80% methanol. All samples were subjected to at least three freeze–thaw cycles, followed by centrifugation at 16,000 ×  *g* for 15 min to precipitate macromolecules. Subsequently, the supernatant (and myristic-d_27_ as an internal standard) of those samples was evaporated by vacuum centrifugation.

### Gas chromatography-mass spectrometry (GC-MS)

Derivatization of extracted metabolites was performed with methoxyamine (MOX) and N-(*tert*-butyldimethylsilyl)-N-methyl-trifluoroacetamide + 1% *tert*-butyldimethylsilyl (MTBSTFA + 1% TBDMS, Thermo Fisher Scientific). Dried polar metabolites of extracted samples were dissolved in 20 µL of MOX by vortexing thoroughly, followed by centrifugation and incubation at 37 °C for 60 min. Thereafter, 30 µL TBDMS was added, and samples were again incubated for 30 min at 60 °C. Alternatively, samples were derivatized by incubation with 40 µL anhydrous pyridine containing MOX (10 mg/ml) for 15 min, and 80 µL TBDMS for 60 min. Both incubation steps were performed at 70 °C. Measurements of derivatized samples was performed at the Core Facility for Mass Spectrometry at the Medical University of Graz or at the Knapp Center for Biomedical Research at the University of Chicago. For separation and detection of metabolites, the Agilent 7890B GC system coupled to an Agilent 5977 A Inert MS system (Agilent, Santa Clara, CA, USA) was used. GC was performed on a DB-35ms column flushed with helium as a carrier gas at a flow rate of 1 mL/min. A volume of 1 µL sample was injected in splitless mode with an inlet temperature of 270 °C. The oven was kept at 100 °C for 3 min and ramped to 300 °C with a gradient of 3.5 °C/min. Mass spectrometry was performed in Electron ionization (EI) mode at 70 eV by scanning the mass range *m/z* 100–605. Alternatively, the Agilent 7890 GC System coupled to an Agilent 5977B Mass Selective Detector was used. The column for separation was a HP-5ms, flushed with helium at a flow rate of 1.4754 mL/min. Samples were injected in a volume of 1 or 3 µL in splitless mode at an inlet temperature of 280 °C. Initial oven temperature was 60 °C for 1 min, following a ramp of 10 °C increase per minute up to 320 °C. El-Maven software (Agrawal et al, 2019) was used for peak quantification and correction for natural abundance was performed with IsoCor (Millard et al, 2019). Total abundances of metabolites were calculated by normalization to the internal standard (norvaline or myristic-d_27_ acid) and protein content measured with the Millipore BCA Protein Assay Kit (Sigma-Aldrich). For absolute quantification of glucose and mannose in media supernatants, each unlabeled sample was spiked with respective ^13^C-standards. Linearity was confirmed by standard dilutions. The concentration was calculated as follows: concentration^sample^ =  area^sample^/(area^standard^/concentration^standard^). Uptake rates were calculated as (amount^baseline^-amount^endpoint^)/time/cell count^endpoint^. Endpoint levels were assessed in media after 24 h of incubation on A549 cells. Cell count was measured on EVE^TM^ Automated Cell Counter (NanoEntek, Seoul, South Korea).

### Liquid chromatography-mass spectrometry (LC–MS)

For extraction of metabolites from in vitro cultured cells, wells were washed with saline and scraped in 80% MeOH on dry ice. Cell extracts in 80% MeOH were sonicated for 3 min at 0 °C followed by 5 min of mixing using a Thermomixer (Eppendorf) held at 4 °C. After incubation at 4 °C for 20 min and centrifugation at 18,000 rcf and 4 °C for 20 min, the supernatant was collected and evaporated to dryness using a centrifugal evaporator (Genevac EZ-2 4.0). Solid residues were stored at -80 °C until analysis. On the day of analysis, samples were resuspended in 80 μL of 3:2 MeCN:H_2_O, as described above. After centrifugation, 40 μL of supernatant was collected for LC–MS analysis. A pooled quality control sample was prepared with the remaining volume of supernatant from each sample.

For tissue sample preparation, frozen tissue pieces were pulverized to a powder in a liquid nitrogen chilled mortar and weighed. Extraction solvent (2:2:1 MeCN:MeOH:H_2_O) was added to the powdered tissue at a fixed ratio of 30 μL/mg at −80 °C, vortexed briefly, and then brought to 0 °C. Samples were homogenized using a bead mill homogenizer (Omni International, Kennesaw, GA, USA), sonicated at 0 °C, and subjected to a freeze–thaw cycle; these steps were repeated once. After incubation at 0 °C for 20 min and centrifugation at 18,000 rcf and 4 °C for 20 min, 100 μL aliquots of the supernatant were collected and evaporated to dryness using a centrifugal evaporator (Genevac EZ-2 4.0). Solid residues were stored at −80 °C until analysis. On the day of analysis, 60 μL of 3:2 MeCN:H_2_O were added to the solid residues at 0 °C. Samples were resuspended by sonication for 3 min at 0 °C followed by 5 min incubation of mixing using a Thermomixer (Eppendorf) held at 4 °C. After incubation at 4 °C for 20 min and centrifugation at 18,000 rcf and 4 °C for 20 min, 40 μL of supernatant was collected for LC–MS analysis. A pooled quality control sample was prepared with the remaining volume of supernatant from each sample.

Analyte separation and detection were performed using a Thermo Fisher Scientific Vanquish Horizon UHPLC system equipped with an iHILIC-(P) Classic column (2.1 × 150 mm, 5 µm; part # 160.152.0520; HILICON AB) and an Orbitrap IQ-X Tribrid mass spectrometer (Thermo Fisher Scientific). Mobile phase A (MPA) was 20 mM [NH_4_][HCO_3_] adjusted to pH = 9.6 using [NH_4_][OH]; mobile phase B (MPB) was MeCN. The column temperature, injection volume, and the flow rate were 40 °C, 2 µL, and 0.2 mL/min, respectively. The chromatographic gradient was: 0 min, 85% B; 0.5 min, 85% B: 18 min, 20% B; 20 min, 20% B; 20.5 min, 85% B; and 28 min, 85% B. MS parameters were as follows: spray voltage, 3600 V for positive ionization and 2800 for negative ionization modes; sheath gas, 35, auxiliary gas, 5; sweep gas, 1; ion transfer tube temperature, 250 °C; vaporizer temperature, 350 °C; AGC target, 100%; and a maximum injection time of 118 ms. Data acquisition was performed using the Xcalibur software package (Thermo Fisher Scientific) in full-scan mode with a range of 70–1000 *m/z* at 60,000 resolution. Metabolite identification was performed by matching the retention time and, when possible, the MS/MS fragmentation pattern to reference standards in an in-house library. Data analysis, including correction for natural isotopic abundance and tracer purity, was performed using either Compound Discoverer 3.3 (Thermo Fisher Scientific), or Skyline (Adams et al, 2020) in combination with IsoCor (Millard et al, 2019). Total abundances of metabolites from in vitro cultured cells were calculated by normalization to the cell count measured on the TC20 Automated Cell Counter (Bio-Rad) in parallel wells.

Alternatively, methanolic cell suspensions were evaporated using a SpeedVac (Thermo Fisher Scientific). After addition of 400 µl cold MeOH (−20 °C) and 100 µl cold ddH_2_O samples were sonicated using a Bioruptor Pico (30 min, 4 °C, 30 s ON/30 s OFF, frequency high; Diagenode). After further addition of 300 µl ddH_2_O and 900 µl methyl tert-butyl ether (MTBE) samples were incubated under constant shaking (4 °C, 15 min, 800 rpm) and centrifuged (13,000 rpm, 10 min, 4 °C). In total, 800 µl of the upper phase were removed and replaced by 800 µl of an artificial upper phase (MTBE/MeOH/ddH2O, 9/4/4, v/v/v). After anew incubation and centrifugation, as described above, the complete upper phase was removed and 700 µl of the lower phase were collected and dried. Metabolites were resolved in 100 µl 70% acetonitrile (1 mM medronic acid) and used for LC–MS analysis. Protein precipitates were dried, solubilized in 200 µl NaOH (0.3 N, 55 °C, >4 h) and the protein content was determined using Pierce™ BCA reagent (Thermo Fisher Scientific) according to the manufacturer’s guidelines. Aliquots of all samples were combined into a pooled quality control sample (QC) which was analyzed at the start and end of the sequence as well as after each fifth sample injection. Two empty extractions (sample-free for background control) were injected before and after sample injections. An in-house mixture of reference metabolites (for level 1 identification) was injected at the start of the sequence. Each sample matrix-free injection was followed by two QC injections for column re-equilibration.

Chromatographic separation was performed on a Vanquish system (Thermo Fisher Scientific) equipped with an ACQUITY UPLC BEH Amide column (2.1 × 150 mm, 1.7 µm; Waters), using an 18 min gradient (400 µl/min) from 97% solvent A (ACN/ddH_2_O, 95/5, v/v; 10 mM NH_4_FA, 10 mM NH_3_) to 65% solvent B (ddH_2_O/ACN, 95/5, v/v; 20 mM NH_4_FA, 20 mM NH_3_). The column compartment was kept at 40 °C. For metabolite analysis, an Orbitrap Eclipse Tribrid mass spectrometer (Thermo Fisher Scientific) equipped with a heated electrospray ionization source (OptaMax NG) was used for detection of the metabolites in negative acquisition mode (MS1/MS2: *m/z* 80–900, resolution 120,000, AGC target 3e5, IT auto).

Metabolites were identified either level 1 via accurate *m/z* of the [M–H]− ion ( < 5 ppm) and comparison of the retention time (rt) and MS2 spectra to synthetical reference compounds or level 2 without reference rt. All peaks (QC) were manually inspected in Freestyle (1.8 SP2, Thermo Fisher Scientific) and peak extraction of the monoisotopic peak and regarding isotopes of interest was performed in Skyline (12 C/13 C, 26.1.0.057) (Adams et al, 2020). Correction for natural abundance was performed with IsoCor (Millard et al, 2019), and total abundances were calculated by normalization to the internal standard (glutarate) and protein content.

### Statistical analysis

Data were compiled and analysed with GraphPad Prism 10 (GraphPad, Boston, MA, USA). Group differences were calculated using two-sided unpaired Student’s *t* test, Mann–Whitney *U* test, one-way ANOVA with Dunnett’s or Tukey’s post-hoc analysis, or two-way ANOVA with Dunnett’s or Šidák’s multiple comparison test, as appropriate. Data are represented as mean +/− standard error of the mean (SEM). *P* values smaller than 0.05 were considered significant.

## Supplementary information

Peer Review File

Appendix

Source data Fig. 1

Source data Fig. 2

Source data Fig. 3

Source data Fig. 4

Source data Fig. 5

Source data Fig. 6

## Data Availability

Source data for main figures including uncropped images of western blots, and the gating strategy for FACS analysis (Appendix Fig. S8) are provided with this paper. Raw mass spectrometry data are available via Zenodo (https://doi.org/10.5281/zenodo.20325511): https://zenodo.org/records/20325511. The source data of this paper are collected in the following database record: biostudies:S-SCDT-10_1038-S44319-026-00874-6.
