# Supplementary material for: Mannose supports lung cancer metabolism during glycolytic limitation
Source: EMBO Rep. 2026 Aug 7;27(18):5365–81. doi: 10.1038/s44319-026-00874-6 (PMC13601547; doi:10.1038/s44319-026-00874-6)
Supplement: Supplementary file 1 [file 44319_2026_874_MOESM1_ESM.pdf]

EMBO reports

Mannose supports lung cancer metabolism during glycolytic limitation

Theresa Haitzmann, Niklas Thompson, Hardik Shah, Katharina Schindlmaier, Joseph Jelwan, Thomas Eichmann, Nia Hammond, Maia Clare, Robert Cameron, Brandon Faubert, and Katharina Leithner

Corresponding author(s): Katharina Leithner (katharina.leithner@medunigraz.at) , Brandon Faubert (bfaubert@bsd.uchicago.edu)

Review Timeline:

Transfer Date:	17th Feb 26
Editorial Decision:	18th Feb 26
Revision Received:	22nd May 26
Editorial Decision:	19th Jun 26
Revision Received:	30th Jun 26
Accepted:	7th Jul 26

Editor: Achim Breiling

Transaction Report: The first round of review of this manuscript was performed at another journal.

(Note: With the exception of the correction of typographical or spelling errors that could be a source of ambiguity, letters and reports are not edited. Depending on transfer agreements, referee reports obtained elsewhere may or may not be included in this compilation. Referee reports are anonymous unless the Referee chooses to sign their reports.)

Dear Dr. Leithner,

Thank you for transferring your manuscript that has been reviewed at a journal outside EMBO press to EMBO reports. I now went again through the manuscript and the referee reports (attached also below) and your revision plan. The referees mention several concerns and suggestions to improve the manuscript, indicating that a major revision of the manuscript is necessary to allow publication of the study at EMBO reports. Looking through your revision plan and the experiments you suggest to do, it seems that the referee points will be adequately addressed.

I thus invite you to revise your manuscript with the understanding that the concerns of the referees must be addressed in the revised manuscript and in a detailed point-by-point response, as indicated in your revision plan.

Acceptance of your manuscript will depend on a positive outcome of a second round of review using the same referees. It is EMBO reports policy to allow a single round of revision only and acceptance of the manuscript will therefore depend on the completeness of your responses included in the next, final version of the manuscript.

Revised manuscripts should be submitted within three months of a request for revision. Please contact me to discuss the revision (also by video chat) if you have questions or comments regarding the revision, or should you need additional time.

When submitting your revised manuscript, please also carefully review the instructions that follow below.

PLEASE NOTE THAT upon resubmission revised manuscripts are subjected to an initial quality control prior to exposition to re-review. Upon failure in the initial quality control, the manuscripts are sent back to the authors, which may lead to delays. Frequent reasons for such a failure are the lack of the data availability section (please see below) and the presence of statistics based on n=2 (the authors are then asked to present scatter plots or provide more data points).

When submitting your revised manuscript, we will require:

1) a .docx formatted version of the final manuscript text (including legends for main figures, EV figures and tables), but without the figures included. Figure legends should be compiled at the end of the manuscript text.

2) individual production quality figure files as .eps, .tif, .jpg (one file per figure), of main figures and EV figures. Please upload these as separate, individual files upon re-submission.

Two main article types are available:

- Research articles, with up to 10 main figures, with additional material provided as Expanded View items (see below) and unlimited references.

- Rports, that are limited to 25,000 characters (excluding spaces, methods, figure legends and references), with up to 5 figures or tables and unlimited references. Expanded View data are permitted but should be restricted to 5 EV figures.

Please decide after your revision which format fits best to the volume of data you want to present.

The Expanded View format, which will be displayed in the main HTML of the paper in a collapsible format, has replaced the Supplementary information. For an Article you can submit up to 6 images as Expanded View. Please follow the nomenclature Figure EV1, Figure EV2 etc. The figure legend for these should be included in the main manuscript document file in a section called Expanded View Figure Legends after the main Figure Legends section. Additional Supplementary material should be supplied as a single pdf file labeled Appendix. The Appendix should have page numbers and needs to include a table of content on the first page (with page numbers) and legends for all content. Please follow the nomenclature Appendix Figure Sx, Appendix Table Sx etc. throughout the text, and also label the figures and tables according to this nomenclature.

For more details, please refer to our guide to authors:
https://link.springer.com/journal/44319/submission-guidelines#cms-Revised-submissions

Please consult our guide for figure preparation:
https://media.springernature.com/original/springer-cms/rest/v1/content/27825798/data/v1

3) a .docx formatted letter INCLUDING the reviewers' reports and your detailed point-by-point responses to their comments. As part of the EMBO Press transparent editorial process, the point-by-point response is part of the Review Process File (RPF), which will be published alongside your paper.

4) a complete author checklist, which you can download from our author guidelines

(https://link.springer.com/journal/44319/submission-guidelines#cms-Revised-submissions). Please insert page numbers in the checklist to indicate where the requested information can be found in the manuscript. The completed author checklist will also be part of the RPF.

Please also follow our guidelines for the use of living organisms, and the respective reporting guidelines: https://link.springer.com/partners/embo-press/editorial-policies#Animal%20and%20human%20studies

5) that primary datasets produced in this study (e.g. RNA-seq, ChIP-seq, structural and array data) are deposited in an appropriate public database. If no primary datasets have been deposited, please also state this in a dedicated section (e.g. 'No primary datasets have been generated and deposited'), see below.

See also: https://link.springer.com/partners/embo-press/editorial-policies#Data%20deposition

Please remember to provide a reviewer password if the datasets are not yet public.

The accession numbers and database should be listed in a formal "Data Availability" section that follows the model below. This is now mandatory (like the COI statement). Please note that the Data Availability Section is restricted to new primary data that are part of this study. This section is mandatory. As indicated above, if no primary datasets have been deposited, please state this in this section

Data availability

The datasets produced in this study are available in the following databases:

- RNA-Seq data: Gene Expression Omnibus GSE46843 (https://www.ncbi.nlm.nih.gov/geo/query/acc.cgi?acc=GSE46843)
- [data type]: [name of the resource] [accession number/identifier/doi] ([URL or identifiers.org/DATABASE:ACCESSION])

*** Note - All links should resolve to a page where the data can be accessed. ***

See also: https://link.springer.com/partners/embo-press/editorial-policies#Data%20availability%20statement

Moreover, I have these editorial requests:

6) We now request the publication of original source data with the aim of making primary data more accessible and transparent to the reader. You will receive a separate email with instructions for providing source data with your revised manuscript, including information how to upload and organize the files.

7) Our journal encourages inclusion of *data citations in the reference list* to directly cite datasets that were re-used and obtained from public databases. Data citations in the article text are distinct from normal bibliographical citations and should directly link to the database records from which the data can be accessed. In the main text, data citations are formatted as follows: "Data ref: Smith et al, 2001" or "Data ref: NCBI Sequence Read Archive PRJNA342805, 2017". In the Reference list, data citations must be labeled with "[DATASET]". A data reference must provide the database name, accession number/identifiers and a resolvable link to the landing page from which the data can be accessed at the end of the reference. Further instructions are available at: https://link.springer.com/journal/44319/submission-guidelines#cms-Reference-guidelines

8) Regarding data quantification and statistics, please make sure that the number "n" for how many independent experiments were performed, their nature (biological versus technical replicates), the bars and error bars (e.g. SEM, SD) and the test used to calculate p-values is indicated in the respective figure legends (also for EV and Appendix figures). Please also check that all the p-values are explained in the legend, and that these fit to those shown in the figure. Please provide statistical testing where applicable. Please avoid the phrase 'independent experiment' but clearly state if these were biological or technical replicates. Please also indicate (e.g. with n.s.) if testing was performed, but the differences are not significant. In case n=2, please show the data as separate datapoints without error bars and statistics. See also: https://link.springer.com/journal/44319/submission-guidelines#cms-Figure-and-data-presentation

If n<5, please show single datapoints for diagrams.

9) Please add scale bars of similar style and thickness to all microscopic images, using clearly visible black or white bars (depending on the background). Please place these in the lower right corner of the images themselves. Please do not write on or near the bars in the image but define the size in the respective figure legend.

10) Please also note our reference format: https://link.springer.com/journal/44319/submission-guidelines#cms-Reference-guidelines

11) We updated our journal's competing interests policy in January 2022 and request authors to consider both actual and

perceived competing interests. Please name this section 'Disclosure and Competing Interests Statement' and put it after the Acknowledgements section.
See: https://link.springer.com/partners/embo-press/editorial-policies#Competing%20interest%20disclosures

12) We now use CRediT to specify the contributions of each author in the journal submission system. CRediT replaces the author contribution section. Please use the free text box to provide more detailed descriptions and do NOT provide your final manuscript text file with an author contributions section. See also our guide to authors (section 'Author contributions'):
https://link.springer.com/journal/44319/submission-guidelines#cms-Revised-submissions

13) All Materials and Methods need to be described in the main text using our 'Structured Methods' format, which is required for all research articles. According to this format, the Methods section should include a Reagents and Tools Table (listing key reagents, experimental models, software, and relevant equipment and including their sources and relevant identifiers), uploaded as separate file, and a Methods section in which we encourage the authors to describe their methods using a step-by-step protocol format with bullet points, to facilitate the adoption of the methodologies across labs. More information on how to adhere to this format as well as downloadable templates (.doc) for the Reagents and Tools Table can be found in our author guidelines (section 'Structured Methods'):

https://link.springer.com/journal/44319/submission-guidelines#cms-Manuscript-organisation-and-formatting

Please add all primer or antibody information directly to the Reagents and Tools table.

14) Please order the manuscript sections like this, using only these names:
Title page - Abstract (max. 175 words) - Keywords (max. 5) - Introduction - Results - Discussion - Methods - Data availability section - Acknowledgements (please include here all the funding information) - Disclosure and Competing Interests Statement - References - Figure legends - Expanded View Figure legends

15) Please make sure that all the funding information is also entered into the online submission system and that it is complete and similar to the one in the acknowledgement section of the manuscript text file.

We would also welcome the submission of cover suggestions, or motifs to be used by our Graphics Illustrator in designing a cover.

As part of the EMBO publication's Transparent Editorial Process, EMBO reports publishes online a Review Process File (RPF) to accompany accepted manuscripts. This File will be published in conjunction with your paper and will include the referee reports, your point-by-point response and all pertinent correspondence relating to the manuscript.

You are able to opt out of this by letting the editorial office know (emboreports@embo.org). If you do opt out, the Review Process File link will point to the following statement: "No Review Process File is available with this article, as the authors have chosen not to make the review process public in this case."

I look forward to seeing a revised version of your manuscript when it is ready. Please let me know if you have questions or comments regarding the revision.

Kind regards,

Achim

Achim Breiling
Senior Editor
EMBO Reports

Referee #1

In this manuscript, the authors report that mannose can be metabolized in glycolysis and glycosylation pathway in lung adenocarcinoma cells. Under the conditions where glucose is limited or 2-DG is present, more mannose contributes to glycolysis than the control culture conditions. Mannose partially improved antiproliferative effects of glucose deprivation or 2-DG. A PDX model was used to support the effects of 2-DG on mannose utilization in tumors. From these observations, the authors concluded that mannose is an alternative nutrient in lung cancer.

It has long been known that mannose is an important carbon source of GDP-mannose for glycosylation, while mannose-derived

mannose 6-phosphate can be shuttled to the glycolysis by MPI. Because glucose is a primary carbon source contributing to glycolytic metabolite pools, it is not surprising that glucose deprivation increases the apparent contribution of mannose to glycolysis. 2-DG complicates cellular metabolism not only in glycolysis but also in glycosylation, the effects of which were not equivalent to glucose deprivation. Tracer metabolomics was performed in a phase of isotopic steady-state in cell culture, which hampered data interpretation. The issue of how glycolytic and less glycolytic LUAD cells differ in 2-DG resistance is not addressed. Overall, the findings of this study strongly support the existing concept that mannose is metabolized in glycolysis and glycosylation pathways.

Referee #2

In this manuscript, Haitzmann et al. combine in vitro experiments with in vivo tracer studies in lung cancer models to examine how mannose contributes to nucleotide-sugar precursors for glycosylation as well as glycolytic intermediates. The authors emphasize that these contributions become more apparent when glycolysis is perturbed by 2-deoxyglucose (2-DG) or glucose limitation. The in vivo tracing and isotope-labeling measurements are technically demanding and well executed. Several points, however, would benefit from additional clarification and/or targeted experiments.

Major:
1. The title frames mannose as an "alternative nutrient in lung cancer," yet much of the evidence comes from lung cancer-derived cell lines and subcutaneous xenografts (the PDX is a strength). These models do not fully capture the lung microenvironment that may shape nutrient availability and utilization. The central conclusion would be substantially strengthened by a more physiologically relevant in vivo lung cancer model. On the other hand, specificity controls (e.g., mannose versus galactose, or tumor versus matched normal lung tissue) could also strengthen the claim.

2. The data support mannose entry into both (i) nucleotide-sugar pools relevant to glycosylation and (ii) glycolytic intermediates. It would be important to clarify which route (or whether both) is functionally required for the observed growth/survival phenotypes under glycolytic stress. Experiments that more cleanly separate "mannose-to-glycosylation" from "mannose-to-glycolysis" would help identify the dominant mechanism and sharpen downstream therapeutic implications.

3. The rescue observed with 100 µM mannose is interesting, especially under marked glucose restriction. To better contextualize the magnitude of this effect, it would be helpful to report nutrient uptake/consumption rates of glucose, mannose and other major nutrients such as glutamine at representative time points. This would help interpret whether mannose is acting primarily as a bulk carbon source or as a high-leverage precursor for other biosynthetic demands.

4. If mannose uptake is substantially slower than glucose under these conditions, this could suggest that mannose's key contribution may not be bulk fueling, but rather supporting a bottlenecked biosynthetic function (potentially glycosylation). If the authors have relevant uptake data, explicitly discussing this possibility would improve the mechanistic narrative.

5. The enrichment levels under seemingly related conditions in Fig. 2d and Fig. 2e appear different (e.g., 2-DG 0 mM with 0/0.2 mM glucose and no MPI inhibition with 0/0.2 mM glucose). Because these panels are central to the claim that mannose can contribute to glycolytic intermediates when glycolysis is impaired, it would be helpful to more explicitly document the experimental and analytical comparability between these datasets. Please clarify the analytical method and fragments used, show the labeling profiles (M+0-M+n) before and after natural isotope correction, and include an unlabeled control processed identically (to exclude any artifactual contributions). These would further strengthen confidence in the labeling interpretation.

Minor:
6. Fig. 1c reports no effect of 2-DG on A549 tumor growth in vivo. One alternative explanation is insufficient exposure compared with cell culture conditions. Measuring 2-DG and/or 2-DG-6P levels in plasma and tumor would help exclude this possibility and strengthen the interpretation of the in vivo results.

7. L91-92: the statement that 2-DG "interferes with this pathway" reads as if 2-DG prevents mannose from contributing to glycosylation. Please clarify the intended meaning and revise for precision.

8. Under 2-DG treatment, GDP-mannose/fucose increases, whereas UDP-GlcNAc/GalNAc decreases in cultured cells and appears unchanged in xenografts. This pattern may suggest that mannose preferentially supports specific nucleotide-sugar branches rather than globally elevating all glycosylation precursors. Discussion of potential mechanisms and implications (including whether this selectivity could be therapeutically relevant) would be valuable.

9. Several discussion statements appear stronger than what is directly supported by the data. For example, "rescue proliferation" seems to be partial in some settings, and "significantly increased" may apply only to specific conditions.

Referee #3

In this manuscript, Haitzmann et al. investigate the role of mannose in lung cancer using in vitro models and subcutaneous xenografts. They report that mannose can serve as an alternative carbon source when glucose is limiting or in the context of glucose metabolism inhibition using 2-DG. Metabolic plasticity is an important aspect of tumour cell biology, but the data presented here are preliminary and do not fully support mannose metabolism as a potential vulnerability that can be exploited for therapy.

Main concerns include:

1. In line with previously published data, mannose partially rescues the antiproliferative effects of 2-DG (Fig1h) and of full glucose withdrawal (Fig1k) in vitro. No significant effects of mannose are obtained under severe glucose restriction (0.2 mM) (Fig 1k). Of note, glucose restriction in the tumour microenvironment is caused by several factors, including aberrant vascularisation which could equally affect mannose. Hence, there is no evidence that mannose availability is not restricted when glucose is, and the relatively low concentration of circulating mannose (40-80 µM) does not support a straightforward concept where mannose can make meaningful contributions in vivo when glucose levels are partially restricted. Collectively, intervention experiments are required to support the statement that mannose is an important substrate for lung cancer cells in vivo. This would involve in vivo inhibition of mannose uptake/metabolism (with or without 2-DG treatment) to study effects on tumour growth, disease progression, metastasis, immune invasion etc.

2. Mannose incorporation into GDP-Mannose and GDP-Fucose increases upon treatment with 2-DG in vitro (Fig2A) and in vivo (Fig4A). Alterations in these metabolites may affect glycosylation. However, total levels of GDP/Mannose and GDF-Fucose are not affected by 2DG in vitro and in vivo. To dissect how important the contribution of mannose is under these circumstances, intervention studies need to be performed as above to study glycosylation patterns in response to altered mannose availability. Some in vitro evidence is provided that mannose prevents 2-DG induced ER stress (which can be affected by glycosylation), but this is not supported with in vivo data.

3. Mannose incorporation into glycolytic intermediates is low and not affected by 2-DG in vivo (Fig 4c-d). Similar incorporation of mannose derived carbons into glycolysis (lactate) is observed in healthy organs compared to tumour tissue (Fig 4d-f). Collectively, this does not support tumour cell-specific avidity for mannose in vivo that can be exploited with a realistic therapeutic window.

4. It is worth noting that the authors have only used subcutaneous xenograft models of lung cancer. Given the specific metabolic and immune microenvironment of the lung, it would be important to validate any finding in an orthotopic or genetic model of lung cancer.

5. While the infusion studies are informative to study carbon incorporation, these enriched the levels of mannose in circulation to roughly 700 µM (Figure 3d), which equates to ~10 fold the concentration observed in human blood. This may amplify any contribution of mannose to central carbon metabolism.

Minor comments

The authors incorrectly state that the conversion of mannose to mannose-phosphate, GDP-mannose and GDP-fucose was significantly enhanced upon short-term treatment of 2DG but this does not apply to mannose-phosphate (Figure 4a,b).

POINT-BY-POINT RESPONSE – Haitzmann T. et al.

Reviewer 1

It has long been known that mannose is an important carbon source of GDP-mannose for glycosylation, while mannose-derived mannose 6-phosphate can be shuttled to the glycolysis by MPI. Because glucose is a primary carbon source contributing to glycolytic metabolite pools, it is not surprising that glucose deprivation increases the apparent contribution of mannose to glycolysis.

Reply: We are grateful for this comment. Indeed, it has been shown that mannose is taken up by cells and directly contributes to mannosylation on the one hand, and to glycolysis on the other hand. In our revised version of the manuscript, we expanded the Introduction and referred to the existing literature in more detail. However, the question whether mannose is used in tumors for the different downstream pathways *in vivo* has not been addressed previously, to the best of our knowledge. Tumor cells might potentially differ in mannose use from normal cells since they heavily rely on glucose uptake. Using $^{13}C_6$-mannose infusions we show that mannose is indeed utilized *in vivo* to generate GDP-mannose and GDP-fucose for glycosylations in tumors *in vivo*, but is also shuttled towards glycolysis.

We agree that the question whether an increase of mannose contribution to glycolysis under low glucose conditions is due to enhanced flux or is rather an "apparent increase" due to a lack of glucose and consequently diminished pool sizes cannot be fully answered based on our data. This would require formal flux analysis, which is elaborative and requires a pre-existing model. In fact, our proof-of-concept study provides initial data which can be incorporated into flux models in future studies. We stressed the lack of formal flux analysis in our study in the discussion.

Manuscript changes:

Introduction, lines 61-75: "Studies have shown that exogenous mannose can rescue effects caused by 2-DG treatment in cancer cell lines *in vitro*, including breast cancer (Berthe et al, 2018), rhabdomyosarcoma (Leon-Annicchiarico et al, 2015), and acute lymphoblastic leukemia (Gu et al, 2017). Mannose rescued protein glycosylation in lung cancer cells which was reduced by 2-DG (Luciano-Mateo et al, 2026). Of note, mannose has been found to be elevated in the serum of lung cancer patients (Deja et al, 2014). Physiologically, mannose is obtained from the diet or released from peripheral tissues and is an important precursor used for glycosylation reactions (Alton et al, 1998; Sharma & Freeze, 2011), which are crucial for proper protein function. Mannose is taken up by cells via SLC2A glucose transporters and is phosphorylated by hexokinase to mannose-6-phosphate (Sharma et al, 2014). Upon phosphorylation, mannose can also be shuttled towards glycolysis, as has been shown in fibroblasts (Panneerselvam & Freeze, 1996; Ichikawa et al, 2014), osteosarcoma cells (Gonzalez et al, 2018) and lung cancer cells (Luciano-Mateo et al, 2026). The contribution of mannose to tumor metabolism and resistance to glycolysis inhibition *in vivo*, however, remains to be characterized."

*Discussion, lines 323-330: A paragraph on flux analysis was included: "Our *in vitro* data showed that the contribution of mannose to glycolysis was clearly increased under low glucose conditions. This was partly associated with lower abundance of the respective glycolytic intermediates. Whether low glucose conditions in fact lead to an enhanced flux from mannose-6-P to glycolysis or whether the increased contribution is caused by smaller pool sizes cannot be fully answered based on our data. Formal flux analysis and dynamic labeling strategies would be required to quantify the different pathway activities. However, our study provides initial data that can be incorporated in flux models in future studies."

2-DG complicates cellular metabolism not only in glycolysis but also in glycosylation, the effects of which were not equivalent to glucose deprivation.

Reply: Thank you for pointing this out. We included both, 2-DG and no/low glucose treatment in the study, since both are relevant in the context of tumor growth and/or treatment. In the first version of our manuscript, we analyzed the fate of ^{13}C-mannose in 2-DG treated cells and found a contribution to both, glycosylation precursors and glycolytic intermediates. In the revised version, we included novel data showing the conversion of mannose to glycosylation precursors and glycolytic intermediates also in no/low glucose conditions. We not only confirmed the conversion of ^{13}C-mannose to GDP-mannose and GDP-fucose. Under 0.2 mM or 0 mM glucose, also labeling of glycolysis intermediates and ribose-5-phosphate (via the pentose phosphate pathway) was found, reflected also by higher labeling in UDP and GDP-linked sugar-nucleotides (new Fig. 2B and 3B). This shows that mannose in fact fuels glycolysis and the pentose phosphate pathway (PPP), branching from glycolysis, under glucose deprivation.

Manuscript changes:

*Results, lines 136-138: "In A549 cells, $^{13}C_6$-mannose contributed to ~40-60% of GDP-mannose and GDP-fucose pools in control conditions, and we found a significant increase in enrichment upon treatment with 2-DG (Fig. 2A) or glucose starvation media (Fig. 2B)."

*Results, lines 150-159: "Under treatment with low (0.2 mM) or glucose-free media, mannose showed enhanced conversion to GDP-mannose and GDP-fucose, as well as UDP-glucose and UDP-GlcNAc/UDP-GalNAc (Fig. 2B). However, increased M+11 fractions suggested that mannose was also converted to the ribose moiety of the respective nucleotide (UDP/GDP, Fig. 2C). Ribose-5-phosphate (ribose-5-P) can be generated from mannose after conversion to fructose-6-phosphate via MPI (Fig. 2C). In fact, M+5 labeled ribose-5-P was found under low (0.2 mM) and no glucose treatment (Fig. 2B), showing the conversion of mannose to upper glycolysis and the pentose phosphate pathway (PPP). Glucose starvation reduced the overall total abundance of most nucleotide sugars and ribose-5-P in A549 cells, while GDP-mannose was unchanged and GDP-fucose levels were significantly enhanced (Appendix Fig. S2B)."

* Figure 2B was added showing ^{13}C-mannose conversion to glycosylation precursors under high, low and no glucose treatment.

* Figure 3B was added showing ^{13}C-mannose conversion to glycolytic intermediates in low/no glucose conditions.

* Appendix Fig. S2B and Fig. 3D were added showing total metabolite levels.

Tracer metabolomics was performed in a phase of isotopic steady-state in cell culture, which hampered data interpretation. The issue of how glycolytic and less glycolytic LUAD cells differ in 2-DG resistance is not addressed.

Reply: Thank you for this important comment. Indeed, we performed the tracing experiments in stable isotopic steady state, which is suitable to determine the carbon source of the respective metabolite, since this was the prime question in our study. Dynamic labeling would be ideal to assess differences in pathway activities between treatments. Unfortunately, dynamic labeling was not performed in our study, but it could provide additional valuable information in the future e.g. to address the activity of the mannose to glycolysis pathway under a variety of conditions (e.g. in highly glycolytic and poorly glycolytic cancer cell lines, etc).

The suggestion to assess the association between 2-DG resistance and glycolysis in different cell lines is interesting. Indeed, it has been shown that in pancreatic cancer cells, sensitivity towards 2-DG, both in terms of ATP depletion and colony-forming capability, was tightly correlated to their glycolytic state: markedly glycolytic cells were highly sensitive, the least glycolytic pancreatic cancer cell line was resistant towards 2-DG as a single treatment (Cheng et al, 2014; DOI:10.1038/bjc.2014.272). While it would be informative to assess the association between basal glycolysis and 2-DG resistance in different lung cancer cell lines, we did not perform such experiments due to the study's focus on mannose metabolism.

Overall, the findings of this study strongly support the existing concept that mannose is metabolized in glycolysis and glycosylation pathways.

Reply: We fully agree that mannose uptake and utilization for glycosylation and glycolysis is an established concept, yet, the context-dependent utilization of mannose in tumors *in vivo* is still poorly understood. Thus, our findings are novel and the study adds an important piece to the puzzle of metabolic adaptation in tumors.

Reviewer 2

Major:

1. The title frames mannose as an "alternative nutrient in lung cancer," yet much of the evidence comes from lung cancer–derived cell lines and subcutaneous xenografts (the PDX is a strength). These models do not fully capture the lung microenvironment that may shape nutrient availability and utilization. The central conclusion would be substantially strengthened by a more physiologically relevant *in vivo* lung cancer model.

Reply: We are grateful for this comment. Indeed, it would be advantageous to study tumor metabolism e.g. in genetic lung cancer models reflecting the tumor microenvironment. However, in these models (e.g. Kras/p53), multiple lesions occur in the lung, and it is technically demanding to separate healthy tissue from tumor for metabolomics analyses. To address mannose uptake in a variety of cancer models, however, would be warranted in the future. We included the limitation of the models used in the study in the discussion.

Manuscript changes:

*Discussion lines 330-333: We included the statement: "As another limitation of the study, subcutaneous tumor models, PDX and A549, were used in immunocompromised mice, which do not fully recapitulate the metabolic microenvironment of lung cancers. Thus, the analysis of mannose utilization in human lung cancers or genetically engineered tumor models would be warranted."

On the other hand, specificity controls (e.g., mannose versus galactose, or tumor versus matched normal lung tissue) could also strengthen the claim.

Reply: We highly appreciate this suggestion. We performed an analysis of lung tissue from $^{13}C_6$-mannose-infused PDX mice and, indeed, qualitative differences in mannose utilization between tumors and lungs were revealed (novel Fig. 6): While mannose contribution to mannose-6-phosphate or GDP-mannose was present in both, tumors and lungs, mannose conversion to GDP-fucose or lower glycolytic intermediates (GAP, DHAP) was absent, indicating a more avid mannose metabolism by the tumors. Interestingly, labeled lactate and pyruvate were found in the lungs, despite the absence of glycolytic intermediate labeling (DHAP and GAP), suggesting that labeled lactate from the blood may give rise to labeled lactate and pyruvate (after conversion by LDH) in the lungs. However, further studies would be needed to assess whole-body mannose metabolism and its contribution to cellular metabolism in different tissues, either directly or upon conversion e.g. to lactate.

We did not address the question, how galactose is metabolized by tumor cells. Galactose is likewise present in the circulation and may be used to generate activated sugars and even glycolytic intermediates. However, the attachment of UDP is required for the conversion of galactose to glycolytic intermediates.

Manuscript changes:

*Results, lines 229-241: "We also examined labeling from $^{13}C_6$-mannose in downstream metabolites in the lungs of PDX-bearing mice (Fig. 6A). While mannose contribution to mannose-phosphate or GDP-mannose was present in both, tumors and lungs, mannose conversion to GDP-fucose or triose-phosphates (GAP, DHAP) was absent in the lungs (Fig. 6B,C). Interestingly, labeled lactate and pyruvate were found in the lungs, despite the lack of GAP and DHAP labeling (Fig. 6D). The total abundance of those metabolites was unchanged across different treatment groups (Appendix Fig. S6A,B). Of note, similar enrichment of labeled

lactate was also found in the blood and liver of mice infused with $^{13}C_6$-mannose (Fig. 6E, total levels Appendix Fig. S6C). Potentially, systemic labeled lactate from peripheral tissues such as the liver, which increased in the blood during infusion (Appendix Fig. S6D) may give rise to labeled lactate and pyruvate (after conversion by LDH) in the lungs. Together, these data indicate greater mannose routing into glycolytic and glycosylation intermediates by the tumors compared to normal lungs."

*Fig. 6 was added showing ^{13}C-mannose conversion to glycosylation precursors and glycolytic intermediates in lungs from PDX-bearing mice.

*Appendix Fig. S6A-C was added showing total abundances.

2. The data support mannose entry into both (i) nucleotide-sugar pools relevant to glycosylation and (ii) glycolytic intermediates. It would be important to clarify which route (or whether both) is functionally required for the observed growth/survival phenotypes under glycolytic stress. Experiments that more cleanly separate "mannose-to-glycosylation" from "mannose-to-glycolysis" would help identify the dominant mechanism and sharpen downstream therapeutic implications.

Reply: We are grateful for the suggestions. In order to further clarify the role of the mannose-to-glycolysis or mannose-to-glycosylation downstream pathways, we performed additional experiments using inhibitors of either route (see below). Moreover, we assessed metabolite abundances in cells with or without mannose supplementation. Exogenous mannose increased total cellular pools of GDP-mannose and GPD-fucose, which were reduced by low glucose treatment (Fig 2D), suggesting that mannose is highly effective in stabilizing pools of activated mannose and fucose under glucose starvation. In line with this model, we showed that the partial rescue of proliferation under 2-DG treatment or glucose deprivation (the latter condition was assessed in a novel set of experiments) by mannose is lost in the presence of an inhibitor of N-linked glycosylation (Fig. 7A).

The mannose-to-glycolysis pathway, mediated by MPI, potentially fuels a variety of downstream pathways branching from glycolysis, depending on the availability of glucose and the cellular requirements. As we showed in the previous version of the manuscript, this pathway is active under low/no glucose conditions, but not under 2-DG treatment. Our experiments with the MPI inhibitor suggest that the pathway in the direction of glycolysis indeed contributes to enhanced proliferation when glucose is low (former Figure 2f, now Fig. 7A). Collectively, our novel and the previous data suggest that the contribution of mannose to GDP-mannose and GDP-fucose is an important downstream fate of mannose, especially in glucose deprived conditions or under glycolysis inhibition. Additionally, the mannose-to-glycolysis route, although absent in 2-DG treatment, may support glycolysis and branching pathways under a lack of glucose.

Manuscript changes:

*Results, lines 248-252: "Tunicamycin treatment alone reduced proliferation, and moreover, reversed the rescue effect of mannose in 2-DG treated lung cancer cells (Fig. 7A, left graph). Similarly, exogenous mannose rescued proliferation of cells treated with low glucose (0.2 mM) media (P=0.05), which was likewise abolished by tunicamycin (Fig. 7A, right graph)."

*Fig. 7A (right graph) was included showing proliferation experiments in low glucose with a glycosylation inhibitor (tunicamycin). Figure 7A left panel is the previous Figure 1j showing the effects of tunicamycin under 2DG treatment.

3. The rescue observed with 100 µM mannose is interesting, especially under marked glucose restriction. To better contextualize the magnitude of this effect, it would be helpful to report nutrient uptake/consumption rates of glucose, mannose and other major nutrients such as glutamine at representative time points. This would help interpret whether mannose is acting primarily as a bulk carbon source or as a high-leverage precursor for other biosynthetic demands.

And 4. If mannose uptake is substantially slower than glucose under these conditions, this could suggest that mannose's key contribution may not be bulk fueling, but rather supporting a bottlenecked biosynthetic function (potentially glycosylation). If the authors have relevant uptake data, explicitly discussing this possibility would improve the mechanistic narrative.

Reply: We highly appreciate this comment and suggestion. We performed uptake measurements of glucose and mannose and found that the uptake rates of mannose are roughly 1/3 of glucose uptake rates, which suggests that glucose is still the preferred nutrient but mannose is a substantial source as well. The findings are in line with the published literature showing that mannose is readily taken up in various cell lines by a high-affinity transporter at Km(uptake) values in the range of mannose blood concentrations (Panneerselvam K and Freeze HH, 1996; DOI: 10.1074/jbc.271.16.9417).

Manuscript changes:

*Results, lines 184-187. "When we assessed glucose and mannose uptake in cells treated simultaneously with mannose (100 µM) and glucose (at low glucose conditions, 0.2 mM) we found that mannose uptake-rates are roughly 1/3 of glucose uptake-rates (Fig. 3F,G). This suggests that glucose is still the preferred nutrient but mannose is a contributing source as well."

*Figure 3FG: Data showing mannose and glucose uptake rates and a time-course of the concentrations of both sugars in the medium supernatant were included.

5. The enrichment levels under seemingly related conditions in Fig. 2d and Fig. 2e appear different (e.g., 2-DG 0 mM with 0/0.2 mM glucose and no MPI inhibition with 0/0.2 mM glucose). Because these panels are central to the claim that mannose can contribute to glycolytic intermediates when glycolysis is impaired, it would be helpful to more explicitly document the experimental and analytical comparability between these datasets. Please clarify the analytical method and fragments used, show the labeling profiles (M+0-M+n) before and after natural isotope correction, and include an unlabeled control processed identically (to exclude any artifactual contributions). These would further strengthen confidence in the labeling interpretation.

Reply: This point is well taken. Indeed, pyruvate and lactate labeling from $^{13}C_6$ mannose in no glucose conditions reached app. 12 and 4% respectively, in the experimental series from former Fig. 2d (now Appendix Figure S3B, showing all isotopologues), but slightly more than twice the value in series from Fig. 2e (now Fig. 3E). These differences were likely caused by two different batches of A549 cells and dialyzed FCS in the two collaborative labs. The GC-MS analytical method slightly differed in the two labs (see Methods). The derivatization method (MOX and TBDMS) and fragments used were the same (Pyruvate m/z 174, Lactate m/z 261).

As mentioned above, we repeated the experiments using LC-MS as a readout, in order to assess the labeling in upper glycolytic intermediates and glycosylation precursors. The LC-MS data (novel Fig. 3B and 3D) confirm a similar label enrichment from $^{13}C_6$-mannose in all

lower glycolytic intermediates (around 30%) in low/no glucose, yet the pools sizes decreased under low/no glucose treatment (discussed in the Discussion). Given the reproducible findings of increased labeling of lactate/pyruvate from mannose under glucose deprivation in the two labs using GC-MS, confirmed by the LC-MS data, we are confident that our enrichment results reflect the actual contribution of ^{13}C from the tracer and do not represent artefacts. We included isotopologue enrichment values from the glycolysis experiments before and after natural isotope correction in labeled and unlabeled samples in the Fig. 3E source file.

Manuscript changes:

* We included isotopologue enrichment values from the glycolysis experiments before and after natural isotope correction in labeled and unlabeled samples in the Fig. 3E source file.

Minor:

6. Fig. 1c reports no effect of 2-DG on A549 tumor growth *in vivo*. One alternative explanation is insufficient exposure compared with cell culture conditions. Measuring 2-DG and/or 2-DG-6P levels in plasma and tumor would help exclude this possibility and strengthen the interpretation of the *in vivo* results.

Reply: To consider the pharmacokinetics of 2-DG would be indeed helpful for the interpretation of the results. We utilized a standard dosage of 2-DG (500 mg/kg i.p., three times/week) which was effective in numerous studies (cited in the paper). 2-DG is rapidly taken up by cells and the effective drug, 2-DG-6P, accumulates. Indeed, the post-peak half-life of 2-DG has been shown to be app. 1 hour after oral gavage in mice (Sun et al, 2024; DOI: 10.1002/prp2.1173) and 5-6 hours in humans (Stein et al, 2010; doi:10.1002/pros.21172). The efficacy of 2-DG to induce glycolysis inhibition is also modulated by the ratio of 2-DG and glucose levels, since 2-DG-6P acts as a competitive inhibitor on the glycolytic enzyme phosphoglucose isomerase (Laussel and Léon, 2020; doi: 10.1016/j.bcp.2020.114213). Therefore, comparisons between *in vitro* and *in vivo* 2-DG efficacy are difficult. It cannot be ruled out that glycolysis inhibition achievable by 2-DG is lower *in vivo* compared to *in vitro* experiments. Therefore, we utilized different concentrations of 2-DG *in vitro*, an equimolar level of 7 mM 2-DG and glucose and 1mM 2-DG, which is slightly above the peak blood concentration achieved in a clinical trial with 2-DG in humans (Raez et al, 2013; cited in the manuscript).

Manuscript changes:

*Results, lines 98-102: We mentioned the fact that a standard dose of 2-DG was injected "Once tumors reached ~65 mm3, mice were treated with 2-DG (500 mg/kg intraperitoneally, 3x/week), or saline control. This dosage of 2-DG has been frequently used in mice and was effective in different tumor models, either alone or in combination with other agents (Maschek et al, 2004; Boutrid et al, 2008; Zeng et al, 2016; Goodwin et al., 2017)."

7. L91–92: the statement that 2-DG "interferes with this pathway" reads as if 2-DG prevents mannose from contributing to glycosylation. Please clarify the intended meaning and revise for precision.

Manuscript changes: The sentence was omitted since the downstream effects of 2-DG are now explained in the Introduction.

8. Under 2-DG treatment, GDP-mannose/fucose increases, whereas UDP-GlcNAc/GalNAc decreases in cultured cells and appears unchanged in xenografts. This pattern may suggest that mannose preferentially supports specific nucleotide-sugar branches rather than globally elevating all glycosylation precursors. Discussion of potential mechanisms and implications (including whether this selectivity could be therapeutically relevant) would be valuable.

Reply: We are grateful for these considerations. As mentioned above, we additionally performed intervention experiments by treating cells with or without mannose under glucose restriction or 2-DG treatment, which confirmed a rescue of GDP-mannose and GDP-fucose levels, but not of other activated sugars (new Fig. 2D and Appendix Figure S2C). Moreover, we found in a new set of experiments, that not only in 2-DG treated cells, but also in low/no glucose treated cells, mannose contributes to a large proportion of GDP-mannose and GDP-fucose, while UDP-glucose and UDP Gal/GlcNAc show a prominent labeling from ribose-5 (in UDP) (new Fig. 2B and Appendix Figure S2B). In line with this concept, mannose tracing *in vivo* showed a preferential fueling of GDP-mannose and GDP-fucose synthesis (now Fig. 5). Collectively, these data show that mannose preferentially supports the generation of activated mannose and fucose, rather than globally elevating all glycosylation precursors. However, under glucose deprivation it may contribute to the nucleotide part of activated sugars. We discussed these data in the discussion.

Manuscript changes:

*Results, lines 160-169: "To investigate the impact of mannose on metabolite pools, we treated the cells with or without exogenous mannose. We found a decrease of GDP-mannose and GDP-fucose levels in glucose starved cells, which was reversed by exogenous mannose. A similar trend was observed in 2-DG treated cells (Fig. 2D). This is in line with the significant contribution of mannose to activated mannose and fucose. Total abundances of UDP-GlcNAc/UDP-GalNAc or UDP-glucose were decreased in 2-DG treated cells in the presence of mannose (Appendix Fig. S2C). Ribose-5-P and lower glycolytic intermediate pools were unchanged by exogenous mannose under 2-DG treatment or glucose deprivation (Appendix Fig. S2C). Together, these data show that mannose contributes to a large proportion of GDP-mannose and GDP-fucose in lung cancer cells *in vitro*, which is further enhanced by 2-DG treatment or glucose starvation."

* Fig 2B and Appendix Fig. S2B were included, showing the fueling of glycosylation precursors by mannose under low and no glucose.

*Fig. 2D and Appendix Fig. S2C were added, showing UDP-mannose and UDP-fucose and other metabolite levels with or without exogenous mannose *in vitro*.

*Discussion, lines 286-297. "Our data show that in A549 cells, GDP-linked (activated) mannose and fucose are mainly derived from ^{13}C-mannose even in the presence of glucose, and that the enrichment of ^{13}C-mannose in these glycosylation precursors reached almost 100% in glucose starved or 2-DG treated cells. We also found increased labeling of GDP-mannose and GDP-fucose *in vivo* in 2-DG treated mouse xenograft tumors infused with $^{13}C_6$-mannose. The increased use of mannose for glycosylation pathways in lung cancer cells *in vitro* and tumors *in vivo* upon treatment with 2-DG suggests a contribution of mannose to the limited efficacy of this intervention. Other activated sugars, such as UDP-GlcNAc/UDP-GalNAc or UDP-glucose, were only marginally labeled *in vivo* and control conditions *in vitro*. These data suggest that mannose preferentially supports mannosylation and fucosylation, rather than globally elevating all glycosylation precursors. In line with this, exogenous mannose restored the levels of GDP-mannose and GDP-fucose in glucose-restricted conditions *in vitro*."

9. Several discussion statements appear stronger than what is directly supported by the data. For example, “rescue proliferation” seems to be partial in some settings, and “significantly increased” may apply only to specific conditions.

Reply and manuscript changes. Thank you for pointing this out.

*We phrased the proliferation results as “partially rescued”.

*We carefully checked the statements on significance. We corrected the previous sentence (Lines 212-214): “In 2-DG-treated mice bearing PDX tumors, the conversion of mannose to mannose-phosphate, GDP-mannose and GDP-fucose was significantly enhanced, especially if applied short-term (st) before tracing (Fig. 4a,b).” was changed to “In PDX tumors of mice treated with 2-DG, the conversion of mannose to GDP-mannose and GDP-fucose was significantly enhanced, if applied short-term (st) before tracing (Fig. 5C).”

Reviewer 3

1. In line with previously published data, mannose partially rescues the antiproliferative effects of 2-DG (Fig1h) and of full glucose withdrawal (Fig1k) *in vitro*. No significant effects of mannose are obtained under severe glucose restriction (0.2 mM) (Fig 1k). Of note, glucose restriction in the tumour microenvironment is caused by several factors, including aberrant vascularisation which could equally affect mannose. Hence, there is no evidence that mannose availability is not restricted when glucose is, and the relatively low concentration of circulating mannose (40-80 μM) does not support a straightforward concept where mannose can make meaningful contributions *in vivo* when glucose levels are partially restricted.

<u>Reply</u>: We thank the reviewer for this comment. Mannose rescued the antiproliferative effects of 2-DG and of complete glucose withdrawal and by trend also in glucose limitation (0.2 mM). To assess whether the rescue effect was present and reproducible also under 0.2 mM glucose, we repeated the experiments in the presence or absence of tunicamycin, an inhibitor of N-linked glycosylation. We obtained similar rescue effects of mannose under low glucose conditions (p=0.05), which were reversed by tunicamycin (new Fig. 7A). These data indicate that mannose indeed rescues proliferation suppressed by 0.2 mM glucose.

The comment that mannose could be equally depleted from the TME as glucose is well taken, however, when we performed uptake rate measurements of glucose and mannose, we found that uptake rates of mannose are roughly 1/3 of glucose uptake rates (novel Fig. 3F,G). If glucose is consumed at higher rates in the tumors, glucose gradients may be steeper than mannose gradients and mannose potentially might still be available in glucose-depleted tumor areas. Our *in vivo* labeling data indeed show a considerable contribution of mannose to glycolytic precursors in the tumors, suggesting avid uptake of mannose despite the relatively low abundance in blood. However, analyses using tumor interstitial fluid, although technically demanding, would be needed to clarify the actual extracellular concentration of mannose in tumors.

<u>Manuscript changes</u>:

* Fig 7A (right graph) was included showing the effect of mannose and tunicamycin in 0.2 mM glucose conditions.

Results lines 250-252: “Similarly, exogenous mannose rescued proliferation of cells treated with low glucose (0.2 mM) media (P=0.05), which was likewise abolished by tunicamycin (Fig. 7A, right graph)."

Results lines 127-129: The results from Fig. 1k (now Fig. 1l), are now described as: “Notably, mannose also partially rescued A549 cell proliferation under glucose deprivation (0.2 or 0 mM glucose), although the effect was significant only with complete glucose withdrawal (Fig. 1l).”

Collectively, intervention experiments are required to support the statement that mannose is an important substrate for lung cancer cells *in vivo*. This would involve *in vivo* inhibition of mannose uptake/metabolism (with or without 2-DG treatment) to study effects on tumour growth, disease progression, metastasis, immune invasion etc.

Reply: We are grateful for this suggestion. Unfortunately, mannose uptake and initial downstream steps cannot be specifically blocked *in vivo*, since mannose and glucose share common transporters (mainly GLUT1) and both are phosphorylated by hexokinase. However, we extended our intervention experiments *in vitro*, by assessing metabolite abundances in cells treated with the respective low glucose media or 2-DG in the absence or presence of exogenous mannose (in physiological concentrations). In line with our results, exogenous mannose increased total cellular pools of GDP-mannose and GPD-fucose, which were reduced by low glucose treatment (Fig 2D), suggesting that mannose is highly effective in stabilizing pools of activated mannose and fucose under glucose starvation. A similar trend was observed in 2-DG treatment (Fig 2D).

*Results, lines 160-169. “To investigate the impact of mannose on metabolite pools, we treated the cells with or without exogenous mannose. We found a decrease of GDP-mannose and GDP-fucose levels in glucose starved cells, which was reversed by exogenous mannose. A similar trend was observed in 2-DG treated cells (Fig. 2D). This is in line with the significant contribution of mannose to activated mannose and fucose. Total abundances of UDP-GlcNAc/UDP-GalNAc or UDP-glucose were decreased in 2-DG treated cells in the presence of mannose (Appendix Fig. S2C). Ribose-5-P and lower glycolytic intermediate pools were unchanged by exogenous mannose under 2-DG treatment or glucose deprivation (Appendix Fig. S2C). Together, these data show that mannose contributes to a large proportion of GDP-mannose and GDP-fucose in lung cancer cells *in vitro*, which is further enhanced by 2-DG treatment or glucose starvation.“

*Fig. 2D was added, showing UDP-mannose and UDP-fucose levels with or without exogenous mannose *in vitro*.

2. Mannose incorporation into GDP-Mannose and GDP-Fucose increases upon treatment with 2-DG *in vitro* (Fig2A) and *in vivo* (Fig4A). Alterations in these metabolites may affect glycosylation. However, total levels of GDP/Mannose and GDF-Fucose are not affected by 2DG *in vitro* and *in vivo*. To dissect how important the contribution of mannose is under these circumstances, intervention studies need to be performed as above to study glycosylation patterns in response to altered mannose availability. Some *in vitro* evidence is provided that mannose prevents 2-DG induced ER stress (which can be affected by glycosylation) but this is not supported with *in vivo* data.

mReply: We fully agree that an alteration of GDP-mannose and GDP-fucose levels upon blocking of mannose uptake would strongly support the concept that mannose is a important precursor of te pathway. As outlined above, specific interference with the mannose pathway *in vivo* is not possible, unless a mannose depletion strategy e.g. mannose-catabolizing enzymes acting on circulating mannose would be available. However, as mentioned above, we performed intervention experiments *in vitro*, by assessing metabolite abundances in cells treated with the respective low glucose media or 2-DG in the absence or presence of exogenous mannose (in physiological concentrations). In line with our results, exogenous mannose increased total cellular pools of GDP-mannose and GPD-fucose, which were reduced by low glucose treatment (novel Fig 2D) and a similar trend was observed upon 2-DG treatment (novel Fig 2D). We did not address glycosylation patterns in this study.

Manuscript changes:

*Results, lines 160-169. “To investigate the impact of mannose on metabolite pools, we treated the cells with or without exogenous mannose. We found a decrease of GDP-mannose and GDP-fucose levels in glucose starved cells, which was reversed by exogenous mannose. A similar trend was observed in 2-DG treated cells (Fig. 2D). This is in line with the significant contribution of mannose to activated mannose and fucose. Total abundances of UDP-GlcNAc/UDP-GalNAc or UDP-glucose were decreased in 2-DG treated cells in the presence of mannose (Appendix Fig. S2C). Ribose-5-P and lower glycolytic intermediate pools were unchanged by exogenous mannose under 2-DG treatment or glucose deprivation (Appendix Fig. S2C). Together, these data show that mannose contributes to a large proportion of GDP-mannose and GDP-fucose in lung cancer cells *in vitro*, which is further enhanced by 2-DG treatment or glucose starvation.“

* Fig. 2D and Appendix Fig. S2C were added, showing UDP-mannose and UDP-fucose and other metabolite levels with or without exogenous mannose *in vitro*.”

3. Mannose incorporation into glycolytic intermediates is low and not affected by 2-DG *in vivo* (Fig 4c-d). Similar incorporation of mannose derived carbons into glycolysis (lactate) is observed in healthy organs compared to tumour tissue (Fig 4d-f). Collectively, this does not support tumour cell-specific avidity for mannose *in vivo* that can be exploited with a realistic therapeutic window.

Reply: We highly appreciate this comment. Indeed, the contribution of labeled mannose to activated mannose and fucose was high, while the contribution to glycolysis was lower. In order to address differences between the tumors and normal tissue, we performed an analysis of lung tissue in addition to the PDX tumors and, indeed, qualitative differences in mannose utilization were revealed (novel Fig. 6): While mannose contribution to mannose-6-phosphate or GDP-mannose was present in both, tumors and lungs, mannose conversion to GDP-fucose or triose-phosphates (GAP, DHAP) was almost absent in the lungs, indicating more avid use of mannose in downstream metabolism by the tumors. Interestingly, labeled lactate and pyruvate were found in the lungs, despite the absence of glycolytic intermediate labeling (DHAP and GAP), suggesting that labeled lactate from the blood may give rise to labeled lactate and pyruvate (after conversion by LDH) in the lungs.

A lack of specificity is a well acknowledged issue in targeting tumor metabolism. Whether inhibiting mannose metabolism would offer a realistic treatment option with an acceptable therapeutic window remains to be studied.

Manuscript changes:

*Results, lines 229-241: “We also examined labeling from $^{13}C_6$-mannose in downstream metabolites in the lungs of PDX-bearing mice (Fig. 6A). While mannose contribution to mannose-phosphate or GDP-mannose was present in both, tumors and lungs, mannose conversion to GDP-fucose or triose-phosphates (GAP, DHAP) was absent in the lungs (Fig. 6B,C). Interestingly, labeled lactate and pyruvate were found in the lungs, despite the lack of GAP and DHAP labeling (Fig. 6D). The total abundance of those metabolites was unchanged across different treatment groups (Appendix Fig. S6A,B). Of note, similar enrichment of labeled lactate was also found in the blood and liver of mice infused with $^{13}C_6$-mannose (Fig. 6E, total levels Appendix Fig. S6C). Potentially, systemic labeled lactate from peripheral tissues such as the liver, which increased in the blood during infusion (Appendix Fig. S6D) may give rise to labeled lactate and pyruvate (after conversion by LDH) in the lungs.

Together, these data indicate greater mannose routing into glycolytic and glycosylation intermediates by the tumors compared to normal lungs."

*Fig. 6 was added showing ^{13}C-mannose conversion to glycosylation precursors and glycolytic intermediates in lungs from PDX-bearing mice.

*Appendix Fig. S6A-C was added showing total abundances.

*Discussion lines 348-349: The sentence "Therefore, manipulating mannose metabolism, such as targeting of MPI or downstream glycosylation pathways might be a promising therapeutic strategy in the future." was changed to "Limiting mannose uptake and metabolism alongside inhibition of glycolysis might be a promising therapeutic strategy in the future.

4. It is worth noting that the authors have only used subcutaneous xenograft models of lung cancer. Given the specific metabolic and immune microenvironment of the lung, it would be important to validate any finding in an orthotopic or genetic model of lung cancer.

Reply: We highly appreciate the suggestion to utilize orthotopic or genetic lung cancer models. Indeed, genetic mouse models better reflect the tumor microenvironment in the target organ. However, in these models (e.g. Kras/p53), multiple lesions occur in the lung, and it is technically demanding to separate healthy tissue from tumor for metabolomics analyses. To address mannose uptake in a variety of cancer models, however, would be warranted in the future. We included the limitation of the models used in the discussion.

Manuscript changes:

*Discussion lines 330-333: We included the statement: "As another limitation of the study, subcutaneous tumor models, PDX and A549, were used in immunocompromised mice, which do not fully recapitulate the metabolic microenvironment of lung cancers. Thus, the analysis of mannose utilization in human lung cancers or genetically engineered tumor models would be warranted."

5. While the infusion studies are informative to study carbon incorporation, these enriched the levels of mannose in circulation to roughly 700 µM (Figure 3d), which equates to ~10 fold the concentration observed in human blood. This may amplify any contribution of mannose to central carbon metabolism.

Reply: Interestingly, mannose levels appear to be higher in mice compared to humans (100 µM; Davis and Freeze, 2001; doi: 10.1016/s0304-4165(01)00183-0). We calculated fold-changes of total mannose before and after infusion, in addition to the previous plot of absolute blood concentrations. The mean elevation of total mannose levels was 3.4-fold (range 2.2-4.9) after the bolus and 1.5-fold (range 0.8 to 2.0) at the end of infusion, compared to baseline. This was considered a reasonable compromise between achieving a sufficient label enrichment in the precursor pool while omitting an excessive increase of precursor levels.

Manuscript changes:

*Results, lines 200-204: "$^{13}C_6$-mannose was administered as a primed-continuous infusion […]. Using this protocol, the mean elevation of total mannose levels was 3.4-fold (range 2.2-4.9) after the bolus and 1.5-fold (range 0.8 to 2.0) at the end of infusion compared to baseline in saline control mice (Fig. 4D)."

Minor comments

The authors incorrectly state that the conversion of mannose to mannose-phosphate, GDP-mannose and GDP-fucose was significantly enhanced upon short-term treatment of 2DG but this does not apply to mannose-phosphate (Figure 4a,b).

Manuscript changes: Thank you for pointing this out. We carefully checked the statements on significance and corrected the previous sentence (Lines 212-214): "In 2-DG-treated mice bearing PDX tumors, the conversion of mannose to mannose-phosphate, GDP-mannose and GDP-fucose was significantly enhanced, especially if applied short-term (st) before tracing (Fig. 4a,b)." was changed to "In PDX tumors of mice treated with 2-DG, the conversion of mannose to GDP-mannose and GDP-fucose was significantly enhanced, if applied short-term (st) before tracing (Fig. 5C)."

Other changes:

1) Thomas Eichmann, Core Facility Mass Spectrometry, Medical University of Graz, who contributed with LC-MS on cells treated with or without exogenous mannose, was added as an author.

2) The Introductory paragraph was divided into “Abstract” and “Introduction” and parts of the “Results” section were moved to “Introduction”.

3) In addition to the described changes, we moved the following Figure panels to enhance clarity:

*All proliferation experiments involving tunicamycin or MPI inhibitor were moved to Fig. 7A,C

*BIP Western blots were moved to Fig. 7B and Appendix Fig. S7A

*All Western blot quantifications were moved to Appendix Fig. S7B

Dear Dr. Leithner,

Thank you for the submission of your revised manuscript to our editorial offices. I have now received the reports from the three referees that were asked to re-evaluate the study, you will find below. As you will see, referees #1 and #3 think that the manuscript has improved but have remaining concerns that need to be addressed in a further revised manuscript. Mostly, this requires reframing and rewriting and moderating statements in the manuscript.

Referee #2 does not support publication of the manuscript, mainly indicating novelty concern. I do not share this concern and think that the manuscript could be published, if revised as indicated by referees #1 and #3, in particular highlighting better its novelty. I thus invite you to revise you manuscript further, as outlined by the referee reports, and to provide a final detailed point-by-point response. Acceptance of your further revised manuscript will depend on a positive outcome of a final round of review, using referees #1 and #3.

Moreover, I have the editorial requests below. Please also provide a p-b-p-response to and these editorial requests with your revised manuscript.

Editorial requests:

- Please provide the abstract written in present tense.

- Please provide e-mail addresses for the corresponding authors on the title page.

- One of the corresponding authors has as one of the affiliations 'BioTechMed-Graz'. Is this a company? If yes, please explain this in the 'Disclosure and competing interest statement' and disclose any related competing interests.

- Please check again that the number "n" for how many independent experiments were performed, their nature (biological versus technical replicates), the bars and error bars (e.g. SEM, SD) and the test used to calculate p-values is indicated in the respective figure legends (main and EV figures). Please also check that all the p-values are explained in the legend, and that these fit to those shown in the figure. Please provide statistical testing where applicable. Please avoid the phrase 'independent experiment' but clearly state if these were biological or technical replicates. Please also indicate (e.g. with n.s.) if testing was performed, but the differences are not significant. In case n=2, please show the data as separate datapoints without error bars and statistics. See also:

https://link.springer.com/journal/44319/submission-guidelines#cms-Figure-and-data-presentation

If n<5, please show single datapoints for diagrams. Moreover:
- Please note that the legends for figure 3 are not provided in the sequential manner (legend for figure 3D is provided before legend of figure 3C;). This needs to be rectified.
- Please note that the exact p values are not provided in the legends of 1A,B,E,G,H,I; 2A,B,D; 3A,B,D,E,G; 5C; 6B; 7A,C.
- Please note that the error bars are not defined in the legends of figure 7A,C.

- Please add callouts to the Reagents & Tools Table to the methods section where appropriate.

- Please remove the author names and affiliations from the Appendix title page. It is sufficient to state 'Appendix for ... (title)' followed by the table of contents.

- Please make sure each figure panel is called out sequentially and separately. Presently, callouts Appendix Fig. S8 seem missing. Moreover, the callout for Appendix Fig. 7B is missing an "S". Please check.

- Please remove now the referee access from the Data Availability section (DAS). Please make sure that the dataset is public latest upon online publication of the study. Please also add a final direct link to the dataset to the DAS.

- Please make sure that all the funding information is also entered into the online submission system and that it is complete and similar to the one in the acknowledgement section of the manuscript text file. Presently, all the funders listed in the Comments box also need to be entered as separate funders each; the comments box should not be used as our publisher cannot retrieve the funding from there. Please fix this.

In addition, I would need from you uploaded separately:
- a short, two-sentence summary of the manuscript (not more than 35 words).
- two to four short (!) bullet points highlighting the key findings of your study (two lines each).
- a schematic summary figure as separate file that provides a sketch of the major findings (not a data image) in jpeg or tiff format

(with the exact width of 550 pixels and the exact height of 300 pixels) that can be used as a visual synopsis on our website. Please upload this separately and remove the graphical abstract from the manuscript text file.

I look forward to seeing the further revised version of your manuscript when it is ready. Please let me know if you have questions regarding the revision.

We would also welcome the submission of cover suggestions, or motifs to be used by our Graphics Illustrator in designing a cover.

As part of the EMBO publication's Transparent Editorial Process, EMBO reports publishes online a Review Process File (RPF) to accompany accepted manuscripts. This File will be published in conjunction with your paper and will include the referee reports, your point-by-point response and all pertinent correspondence relating to the manuscript.

You are able to opt out of this by letting the editorial office know (emboreports@embo.org). If you do opt out, the Review Process File link will point to the following statement: "No Review Process File is available with this article, as the authors have chosen not to make the review process public in this case."

Yours sincerely,

Achim Breiling
Snior Editor
EMBO Reports

Referee #1:

This manuscript investigates mannose metabolism in lung cancer cells and xenografts under glycolytic stress. The major strength of the study is the use of stable isotope tracing, particularly in vivo 13C6-mannose infusion in patient-derived xenograft models, to show that circulating mannose contributes to GDP-mannose and GDP-fucose metabolism in tumors. The study is interesting and has improved from last version. However, the manuscript would benefit from a sharper positioning of its novelty and a clearer organization of the in vivo data. I do not think major new experiments are necessarily required, but the framing and presentation should be revised.

Major comments

1. The novelty relative to recent related work should be clarified. Several aspects of mannose metabolism under glucose deprivation or 2-DG treatment have been reported previously, including mannose effects on glycosylation stress and its entry into glycolytic metabolism. The manuscript should more clearly distinguish which findings are confirmatory and which are new, and this point should be made more explicit in the Introduction and Discussion.
2. The "alternative nutrient" framing may be too broad. The title and some statements imply that mannose broadly functions as an alternative nutrient in lung cancer. However, the strongest data support a more specific conclusion: mannose contributes substantially to GDP-sugar metabolism, especially GDP-mannose and GDP-fucose, under glycolytic stress. The evidence for mannose as a major alternative glycolytic or bioenergetic nutrient is more limited. The title, abstract, and concluding statements should be adjusted to better match the best supported data.
3. The functional link to glycosylation should be interpreted cautiously. The tunicamycin and BiP data support a connection between mannose metabolism and glycosylation-related stress responses. However, the manuscript does not directly analyze glycan structures, specific glycoprotein targets, or mannose incorporation into glycoproteins.
4. The glycolysis labeling data require clearer interpretation. The manuscript shows increased fractional labeling of glycolytic intermediates from 13C6-mannose under glucose deprivation. Although the authors acknowledge this limitation in the Discussion, the distinction between labeling enrichment and actual flux should be made clearer throughout the Results. Statements implying increased mannose flux through glycolysis should be moderated unless directly supported.
5. The in vivo figures should be reorganized to emphasize the main advance. The in vivo tracing data are currently split across Figures 4-6, which makes the message less direct. The authors should consider reorganizing these figures so that the key finding is presented more prominently.

Referee #2:

As an alternative nutrient in tumors, mannose is known to contribute to glycolysis (Nature, 2018; Cancer Sci, 2021). Although tracer metabolomics in this study confirmed contribution of mannose to GDP-mannose/fucose pathway, this metabolic routing of mannose-derived carbon has been well established. Although the current data suggested that 2-DG/glucose deprivation

enhanced the contribution, GDP-mannose/fucose pools can be formed from multiple carbon sources (e.g., glucose, mannose, fucose). 2-DG or glucose deprivation reduces glucose contribution, thereby simply increasing apparent contribution of mannose to these metabolite pools. To this reviewer, the strength of this work is to reveal distinct metabolic consequences between glucose deprivation and 2-DG treatment by using mannose as a tracer. Overall, the work cannot be recommended for publication in its current form.

Referee #3:

In the revised version of this manuscript, Haitzmann et al addressed some of the comments raised during the initial evaluation, while others have not been addressed. In its entirety, the study provides novel information on the metabolism of mannose in lung cancer and may be acceptable for publication in EMBO Reports providing that key conclusions are sufficiently nuanced and limitations are openly discussed. For example, instead of performing additional experiments, the authors have mentioned the lack of disease-relevant models in this study which is appreciated.

Point that have not been addressed appropriately:

• The reduction in mannose availability which will likely co-occur with limited glucose availability. In the rebuttal (but not manuscript), the authors argue that the relatively slower uptake of mannose may sustain availability when glucose becomes limiting. However, this does not take into account the ~70 fold higher concentration of glucose vs mannose in physiology, and their data shows that mannose is only able to rescue cell proliferation when glucose is depleted to 0.2mM, an observation that is also borderline significant. Hence, it is not straightforward to assume a physiological situation where mannose availability will circumvent glucose depletion.

• It is clear that interfering with mannose uptake/metabolism in vivo is challenging. However, at present the study does not unambiguously show that mannose is required to maintain homeostasis of downstream metabolites and processes outside an in vitro context. A more open discussion that the present study cannot rule out that mannose may be dispensable for tumour biology in vivo given tumour cell plasticity and alternative nutrient availability would improve the study. Similarly, the lack of work done to resolve effects of mannose on glycosylation patterns and their relevance for ER stress should be mentioned.

• As raised previously, the incorporation of mannose-derived carbons into glycolytic intermediates is similar between tumour and normal tissues (see Figures 5E and 6D-E). The authors should rephrase the statement below (line 239) accordingly:

'Together, these data indicate greater mannose routing into glycolytic and glycosylation intermediates by the tumors compared to normal lungs.

POINT-BY-POINT REPLY 2 – Haitzmann T. et al.

Referee #1

This manuscript investigates mannose metabolism in lung cancer cells and xenografts under glycolytic stress. The major strength of the study is the use of stable isotope tracing, particularly *in vivo* 13C6-mannose infusion in patient-derived xenograft models, to show that circulating mannose contributes to GDP-mannose and GDP-fucose metabolism in tumors. The study is interesting and has improved from last version. However, the manuscript would benefit from a sharper positioning of its novelty and a clearer organization of the *in vivo* data. I do not think major new experiments are necessarily required, but the framing and presentation should be revised.

Major comments

1. The novelty relative to recent related work should be clarified. Several aspects of mannose metabolism under glucose deprivation or 2-DG treatment have been reported previously, including mannose effects on glycosylation stress and its entry into glycolytic metabolism. The manuscript should more clearly distinguish which findings are confirmatory and which are new, and this point should be made more explicit in the Introduction and Discussion.

Reply: Thank you very much for this excellent suggestion. We highlighted the novelty and major strength of the study, stable isotopic tracing using $^{13}C_6$-mannose *in vivo*, as suggested.

Manuscript changes:

*Introduction, lines 75-77: “The contribution of mannose to tumor metabolism and resistance to glycolysis inhibition *in vivo*, however, remains to be characterized.” was changed to “However, to the best of our knowledge, whether mannose is used for glycosylation and glycolysis in tumor cells *in vivo* remains unknown.”

*Discussion, lines 273-274: A sentence on the novelty of the findings was included: “We show for the first time that mannose contributes to glycosylation and glycolysis in tumors *in vivo*.”

*Discussion, line 291-292: We included a statement to more clearly distinguish already published work from new data provided by the study: “Mannose has been shown to be efficiently used for N-glycan synthesis in fibroblasts (Ichikawa et al, 2014).”

*Discussion, lines 312-313: “As an additional fate of mannose, we investigated its incorporation into the glycolysis pathway.” was changed to “We show that mannose is not only used for glycosylation, but also contributes to the glycolysis pathway in tumors *in vivo*.”

2. The "alternative nutrient" framing may be too broad. The title and some statements imply that mannose broadly functions as an alternative nutrient in lung cancer. However, the strongest data support a more specific conclusion: mannose contributes substantially to GDP-sugar metabolism, especially GDP-mannose and GDP-fucose, under glycolytic stress. The evidence for mannose as a major alternative glycolytic or bioenergetic nutrient is more limited. The title, abstract, and concluding statements should be adjusted to better match the best supported data.

Reply: We agree that introducing mannose as an "alternative nutrient" is rather broad. We changed the title accordingly and provided more specific conclusions in the abstract and “Conclusion” section as suggested.

Manuscript changes:

*Title: Mannose supports lung cancer metabolism upon glycolysis limitation

*Abstract, lines 30-32: “In conclusion, mannose uptake and metabolism represent an adaptive strategy in lung cancer cells under metabolic stress.” was changed to “In conclusion, mannose utilization for glycosylation precursor synthesis represents an adaptive strategy in lung cancer cells under metabolic stress.”

*Introduction, lines 81-83: “Our study shows that mannose metabolism represents an adaptive strategy of cancer cells to respond to glycolysis inhibition or glucose restriction.” was changed to “Our study shows that mannose utilization for the generation of glycosylation precursors represents an adaptive strategy of cancer cells to respond to glycolysis inhibition or glucose restriction.”

*Discussion, lines 358-360: “In summary, our study shows that mannose is an important precursor in lung cancer cells, and that it may contribute to the resistance of lung cancer towards glycolysis inhibition via 2-DG *in vivo*.” was changed to “In summary, our study shows that mannose is an important precursor for glycosylation pathways in lung cancer cells *in vivo*, and that it may contribute to the resistance of lung cancer to glycolysis inhibition via 2-DG.”

3. The functional link to glycosylation should be interpreted cautiously. The tunicamycin and BiP data support a connection between mannose metabolism and glycosylation-related stress responses. However, the manuscript does not directly analyze glycan structures, specific glycoprotein targets, or mannose incorporation into glycoproteins.

Reply: Thank you for pointing this out. We previously mentioned this limitation in the Discussion, however, we made the lack of glycan structure analysis clearer in the revised version.

Manuscript changes:

*Discussion, lines 309-311: “While we did not investigate target glycoproteins or glycolipids showing incorporation of exogenous mannose in lung cancer, this could be the focus of future investigations.” was changed to “As a limitation, we did not investigate glycan structures, mannose incorporation into glycoproteins, or the effects of these processes on ER stress *in vivo*.”

4. The glycolysis labeling data require clearer interpretation. The manuscript shows increased fractional labeling of glycolytic intermediates from 13C6-mannose under glucose deprivation. Although the authors acknowledge this limitation in the Discussion, the distinction between labeling enrichment and actual flux should be made clearer throughout the Results. Statements implying increased mannose flux through glycolysis should be moderated unless directly supported.

Reply: We fully agree that formal flux analysis would be required to draw conclusions about any changes in the mannose-to-glycolysis pathway. In order to more clearly state that we measure contributions from mannose, not fluxes, we included the following sentences:

Manuscript changes:

*Results, lines 175-179: “However, mannose utilization for the glycolytic intermediates dihydroxyacetone phosphate (DHAP), 3-phosphoglycerate (3-PG), phosphoenolpyruvate (PEP) and lactate was significantly increased under glucose deprivation (Fig. 3B, Appendix Fig. S3C).” was changed to “However, mannose contribution to the glycolytic intermediates dihydroxyacetone phosphate (DHAP), 3-phosphoglycerate (3-PG), phosphoenolpyruvate (PEP) and lactate was significantly increased under glucose deprivation (Fig. 3B,C, Appendix Fig. S3C), as shown by enhanced labeled fractions” and lines 180-182: “Thus, enhanced labeling may potentially result from smaller pool sizes.” to “Thus, enhanced labeling may potentially result from smaller pool sizes due to a reduced contribution from glucose. Formal flux analysis would be needed to verify an enhanced glycolysis fueling by mannose under glucose starvation.”

5. The *in vivo* figures should be reorganized to emphasize the main advance. The *in vivo* tracing data are currently split across Figures 4-6, which makes the message less direct. The authors should consider reorganizing these figures so that the key finding is presented more prominently.

Reply: We highly appreciate this suggestion. We reorganized the *in vivo* tracing data figures to achieve a more direct comparison of the results from tumor and lung tissues. Figure 4 remains the same and gives information about the establishment of mannose-infusions, whereas Figures 5 and 6 were merged to one.

Manuscript changes:

*Figures 5 and 6 were merged to Figure 5, UDP-Glucose and UPD-Glc/Gal-NAc graphs of former Figure 5C were moved to Appendix Figure S5B and former Figure 5F to Appendix Figure S5H. Figure Legends and Source data were adjusted accordingly.

Referee #2

As an alternative nutrient in tumors, mannose is known to contribute to glycolysis (Nature, 2018; Cancer Sci, 2021). Although tracer metabolomics in this study confirmed contribution of mannose to GDP-mannose/fucose pathway, this metabolic routing of mannose-derived carbon has been well established. Although the current data suggested that 2-DG/glucose deprivation enhanced the contribution, GDP-mannose/fucose pools can be formed from multiple carbon sources (e.g., glucose, mannose, fucose). 2-DG or glucose deprivation reduces glucose contribution, thereby simply increasing apparent contribution of mannose to these metabolite pools. To this reviewer, the strength of this work is to reveal distinct metabolic consequences between glucose deprivation and 2-DG treatment by using mannose as a tracer. Overall, the work cannot be recommended for publication in its current form.

Reply: As outlined by reviewer #1 and in our manuscript, the novelty and strength of this study, as we believe, is the *in vivo* labeling with ^{13}C-mannose in a tumor model, which has not been reported to the best of our knowledge, combined with a detailed analysis of tracer incorporation into glycosylation precursors and glycolysis.

Referee #3

In the revised version of this manuscript, Haitzmann et al addressed some of the comments raised during the initial evaluation, while others have not been addressed. In its entirety, the study provides novel information on the metabolism of mannose in lung cancer and may be acceptable for publication in EMBO Reports providing that key conclusions are sufficiently nuanced and limitations are openly discussed. For example, instead of performing additional experiments, the authors have mentioned the lack of disease-relevant models in this study which is appreciated.

Point that have not been addressed appropriately:

• The reduction in mannose availability which will likely co-occur with limited glucose availability. In the rebuttal (but not manuscript), the authors argue that the relatively slower uptake of mannose may sustain availability when glucose becomes limiting. However, this does not take into account the ~70 fold higher concentration of glucose vs mannose in physiology, and their data shows that mannose is only able to rescue cell proliferation when glucose is depleted to 0.2mM, an observation that is also borderline significant. Hence, it is not straightforward to assume a physiological situation where mannose availability will circumvent glucose depletion.

Reply: Thank you for your valuable comment, we are fully aware that the combination of plasma mannose levels with decreased glucose levels may not fully recapitulate a physiological tumor interstitial fluid. Our study was intended as a pilot study to generate data on metabolic pathways utilized by mannose in cancer cells, especially under glycolysis limitation. Intriguingly, however, the *in vivo* tracing data recapitulate the mannose labeling patterns observed under glucose limitation (0.2 mM) *in vitro*, suggesting that in the context of a physiological microenvironment, mannose may indeed contribute to glycolysis, although to a limited degree, as we point out.

Manuscript changes:

Discussion, lines 324-328: "Of note, a glucose concentration of 0.2 mM was chosen for *in vitro* experiments, consistent with prior lung cancer studies modeling reduced glucose availability (Haitzmann et al, 2024). However, similar labeling patterns of glycolytic intermediates *in vivo* suggest that mannose may be used for glycolysis *in vivo* when glucose availability is limited."

• It is clear that interfering with mannose uptake/metabolism *in vivo* is challenging. However, at present the study does not unambiguously show that mannose is required to maintain

homeostasis of downstream metabolites and processes outside an *in vitro* context. A more open discussion that the present study cannot rule out that mannose may be dispensable for tumour biology *in vivo* given tumour cell plasticity and alternative nutrient availability would improve the study. Similarly, the lack of work done to resolve effects of mannose on glycosylation patterns and their relevance for ER stress should be mentioned.

Reply: We are grateful for this comment and discussed more openly, that we cannot verify a crucial role of mannose to lung cancer metabolism due to the limited options of interfering with the pathway.

Manuscript changes:

*Discussion, lines 303-306: "Unfortunately, directly blocking mannose uptake to determine its requirement for tumor metabolism *in vivo* was not feasible, as mannose is transported through hexose transporters that also mediate glucose uptake."

*Discussion, lines 309-311: "While we did not investigate target glycoproteins or glycolipids showing incorporation of exogenous mannose in lung cancer, this could be the focus of future investigations." was changed to "As a limitation, we did not investigate glycan structures, mannose incorporation into glycoproteins, or the effects of these processes on ER stress *in vivo*."

• As raised previously, the incorporation of mannose-derived carbons into glycolytic intermediates is similar between tumour and normal tissues (see Figures 5E and 6D-E). The authors should rephrase the statement below (line 239) accordingly:

'Together, these data indicate greater mannose routing into glycolytic and glycosylation intermediates by the tumors compared to normal lungs.

Reply: Indeed, labeling of lactate and pyruvate in a tissue, as found in lungs alongside the tumors, may suggest a contribution of mannose to glycolysis, however, the complete lack of labeling of the upstream intermediates DHAP and GAP and the relatively high labeling of lactate and pyruvate in the blood strongly support an uptake of labeled lactate from the blood stream rather than mannose contribution to (lower) glycolysis in the lungs. In the revised version we described this labeling gap more clearly.

Manuscript changes:

*Results, lines 233-237: For a clearer separation of mannose use in tumors vs lungs, the sentences "While mannose contribution to mannose-phosphate or GDP-mannose was present in both, tumors and lungs, mannose conversion to GDP-fucose or triose-phosphates (GAP, DHAP) was absent in the lungs (Fig. 6B,C). Interestingly, labeled lactate and pyruvate were found in the lungs, despite the lack of GAP and DHAP labeling (Fig. 6D)." were changed to "While mannose contribution to mannose-phosphate and GDP-mannose was present in both tumors and lungs, mannose conversion to GDP-fucose was completely absent in the lungs (Fig. 5G). Interestingly, labeled lactate and pyruvate were detected in the lungs, but

no labeling in the upstream glycolytic intermediates GAP and DHAP was observed (Fig. 5H,I).”

Other changes:

*Former Figure 7 is now Figure 6.

2nd Revision - Editorial Decision 7th Jul 2026

Dr. Katharina Leithner
Medical University of Graz
Division of Pharmacology, Otto Loewi Research Center
Neue Stiftingtalstraße 6
Graz, Styria 8010
Austria

Dear Dr. Leithner,

Thank you for the submission of your final revised manuscript to EMBO reports. I have already forwarded to you the reports I have received from the two referees that were asked to re-evaluate your study, which can be also be found at the end of this email. As the referees now fully support publication of your study, and as you have already made the changes requested by referee #1, I am very pleased to accept your manuscript for publication in the next available issue of EMBO reports. Thank you for your contribution to our journal.

Please make sure that the data deposited externally (Zenodo) is public latest for the online publication of the study.

Your manuscript will be processed for publication by EMBO Press. It will be copy edited and you will receive page proofs prior to publication. Please note that you will be contacted by Springer Nature Author Services to complete licensing and payment information.

You may qualify for financial assistance for your publication charges - either via a Springer Nature fully open access agreement or an EMBO initiative. Check your eligibility: https://link.springer.com/journal/44319/how-to-publish-with-us

Should you be planning a Press Release on your article, please notify the production team via this form in order to coordinate publication and release dates: https://forms.cloud.microsoft/e/eH4A8JVP6g

If you have any questions, please do not hesitate to contact the Editorial Office. Thank you for your contribution to EMBO Reports.

Yours sincerely,

Achim Breiling
Senior Editor
EMBO Reports

Referee #1:

V3 revision improves the manuscript. The authors have narrowed the title and conclusions, clarified the novelty around in vivo ^{13}C-mannose tracing and reorganized the in vivo tumor-versus-lung data into a clearer figure. The remaining issue is wording-level overstatements.

"We show for the first time that mannose contributes to glycosylation and glycolysis in tumors in vivo." This statement should be narrowed. Prior study has already provided in vivo evidence for mannose effects in tumor models, including linking mannose supplementation to glucose-deprivation responses and glycosylation-related stress pathways.

Referee #3:

The authors have sufficiently addressed my concerns.

>>> Please note that it is EMBO Reports policy for the transcript of the editorial process (containing referee reports and your

response letter) to be published as an online supplement to each paper. If you do NOT want this, you will need to inform the Editorial Office via email immediately. More information is available here: https://link.springer.com/partners/embo-press/editorial-policies#Peer%20review

EMBO Press Author Checklist

Corresponding Author Name: Katharina Leithner	
Journal Submitted to: EMBO Reports	
Manuscript Number: EMBOR-2026-63867V3	

USEFUL LINKS FOR COMPLETING THIS FORM
The EMBO Journal - Author Guidelines
EMBO Reports - Author Guidelines
Molecular Systems Biology - Author Guidelines
EMBO Molecular Medicine - Author Guidelines

Reporting Checklist for Life Science Articles (updated January
This checklist is adapted from Materials Design Analysis Reporting (MDAR) Checklist for Authors. MDAR establishes a minimum set of requirements in transparent reporting in the life sciences (see Statement of Task: 10.31222/osf.io/9sm4x). Please follow the journal's guidelines in preparing your
Please note that a copy of this checklist will be published alongside your article.

Abridged guidelines for figures
1. Data
The data shown in figures should satisfy the following conditions:
- the data were obtained and processed according to the field's best practice and are presented to reflect the results of the experiments in an accurate and unbiased manner.
- ideally, figure panels should include only measurements that are directly comparable to each other and obtained with the same assay.
- plots include clearly labeled error bars for independent experiments and sample sizes. Unless justified, error bars should not be shown for technical
- if n<5, the individual data points from each experiment should be plotted. Any statistical test employed should be justified.
- Source Data should be included to report the data underlying figures according to the guidelines set out in the authorship guidelines on Data

2. Captions
Each figure caption should contain the following information, for each panel where they are relevant:
- a specification of the experimental system investigated (eg cell line, species name).
- the assay(s) and method(s) used to carry out the reported observations and measurements.
- an explicit mention of the biological and chemical entity(ies) that are being measured.
- an explicit mention of the biological and chemical entity(ies) that are altered/varied/perturbed in a controlled manner.
- the exact sample size (n) for each experimental group/condition, given as a number, not a range;
- a description of the sample collection allowing the reader to understand whether the samples represent technical or biological replicates (including how many animals, litters, cultures, etc.).
- a statement of how many times the experiment shown was independently replicated in the laboratory.
- definitions of statistical methods and measures:
  - common tests, such as t-test (please specify whether paired vs. unpaired), simple χ2 tests, Wilcoxon and Mann-Whitney tests, can be unambiguously identified by name only, but more complex techniques should be described in the methods section;
  - are tests one-sided or two-sided?
  - are there adjustments for multiple comparisons?
  - exact statistical test results, e.g., P values = x but not P values < x;
  - definition of 'center values' as median or average;
  - definition of error bars as s.d. or s.e.m.

Please complete ALL of the questions below.
Select "Not Applicable" only when the requested information is not relevant for your study.

Materials

Newly Created Materials	Information included in the manuscript?	In which section is the information available? (Reagents and Tools Table, Materials and Methods, Figures, Data Availability Section)
New materials and reagents need to be available; do any restrictions apply?	Not Applicable	

Antibodies	Information included in the manuscript?	In which section is the information available? (Reagents and Tools Table, Materials and Methods, Figures, Data Availability Section)
For **antibodies** provide the following information:<br>- Commercial antibodies: RRID (if possible) or supplier name, catalogue number and or/clone number<br>- Non-commercial: RRID or citation	Yes	Reagents and Tools Table

DNA and RNA sequences	Information included in the manuscript?	In which section is the information available? (Reagents and Tools Table, Materials and Methods, Figures, Data Availability Section)
**Short novel DNA or RNA including primers, probes**: provide the sequences.	Yes	Materials and Methods

Cell materials	Information included in the manuscript?	In which section is the information available? (Reagents and Tools Table, Materials and Methods, Figures, Data Availability Section)
**Cell lines:** Provide species information, strain. Provide accession number in repository **OR** supplier name, catalog number, clone number, and/**OR** RRID.	Yes	Reagents and Tools Table
**Primary cultures:** Provide species, strain, sex of origin, genetic modification status.	Not Applicable	
Report if the cell lines were recently **authenticated** (e.g., by STR profiling) and tested for mycoplasma contamination.	Yes	Materials and Methods

Experimental animals	Information included in the manuscript?	In which section is the information available? (Reagents and Tools Table, Materials and Methods, Figures, Data Availability Section)
**Laboratory animals or Model organisms:** Provide species, strain, sex, age, genetic modification status. Provide accession number in repository **OR** supplier name, catalog number, clone number, **OR** RRID.	Yes	Reagents and Tools Table, Materials and Methods
**Animal observed in or captured from the field:** Provide species, sex, and age where possible.	Not Applicable	
Please detail **housing and husbandry conditions**.	Yes	Materials and Methods

Plants and microbes	Information included in the manuscript?	In which section is the information available? (Reagents and Tools Table, Materials and Methods, Figures, Data Availability Section)
**Plants:** provide species and strain, ecotype and cultivar where relevant, unique accession number if available, and source (including location for collected wild specimens).	Not Applicable	
**Microbes:** provide species and strain, unique accession number if available, and source.	Not Applicable	

Human research participants	Information included in the manuscript?	In which section is the information available? (Reagents and Tools Table, Materials and Methods, Figures, Data Availability Section)
If collected and within the bounds of privacy constraints report on age, sex and gender or ethnicity for all study participants.	Not Applicable	

Core facilities	Information included in the manuscript?	In which section is the information available? (Reagents and Tools Table, Materials and Methods, Figures, Data Availability Section)
If your work benefited from core facilities, was their service mentioned in the acknowledgments section?	Yes	Acknowledgements

Design

Study protocol	Information included in the manuscript?	In which section is the information available? (Reagents and Tools Table, Materials and Methods, Figures, Data Availability Section)
If study protocol has been **pre-registered, provide DOI in the manuscript**. For clinical trials, provide the trial registration number **OR** cite DOI.	Not Applicable	
Report the **clinical trial registration number** (at ClinicalTrials.gov or equivalent), where applicable.	Not Applicable	

Laboratory protocol	Information included in the manuscript?	In which section is the information available? (Reagents and Tools Table, Materials and Methods, Figures, Data Availability Section)
Provide DOI OR other citation details if **external detailed step-by-step protocols** are available.	Not Applicable	

Experimental study design and statistics	Information included in the manuscript?	In which section is the information available? (Reagents and Tools Table, Materials and Methods, Figures, Data Availability Section)
Include a statement about **sample size** estimate even if no statistical methods were used.	Yes	Materials and Methods
Were any steps taken to minimize the effects of subjective bias when allocating animals/samples to treatment (e.g. **randomization procedure**)? If yes, have they been described?	Yes	Materials and Methods
Include a statement about **blinding** even if no blinding was done.	Yes	Materials and Methods
Describe **inclusion/exclusion criteria** if samples or animals were excluded from the analysis. Were the criteria pre-established?<br><br>If sample or data points were omitted from analysis, report if this was due to attrition or intentional exclusion and provide justification.	Yes	Materials and Methods
For every figure, are **statistical tests** justified as appropriate? Do the data meet the assumptions of the tests (e.g., normal distribution)? Describe any methods used to assess it. Is there an estimate of variation within each group of data? **Is** the variance similar between the groups that are being statistically compared?	Yes	Figure Legends

Sample definition and in-laboratory replication	Information included in the manuscript?	In which section is the information available? (Reagents and Tools Table, Materials and Methods, Figures, Data Availability Section)
In the figure legends: state number of times the experiment was **replicated** in laboratory.	Yes	Figure Legends
In the figure legends: define whether data describe **technical or biological replicates**.	Yes	Figure Legends

Ethics

Ethics	Information included in the manuscript?	In which section is the information available? (Reagents and Tools Table, Materials and Methods, Figures, Data Availability Section)
Studies involving **human participants**: State details of **authority granting ethics approval** (IRB or equivalent committee(s), provide reference number for approval.	Not Applicable	
Studies involving **human participants**: Include a statement confirming that **informed consent** was obtained from all subjects and that the experiments conformed to the principles set out in the WMA Declaration of Helsinki and the Department of Health and Human Services Belmont Report.	Not Applicable	
Studies involving **human participants:** For publication of **patient photos**, include a statement confirming that consent to publish was obtained.	Not Applicable	
Studies involving experimental **animals**: State details of **authority granting ethics approval** (IRB or equivalent committee(s), provide reference number for approval. Include a statement of compliance with ethical regulations.	Yes	Materials and Methods
Studies involving **specimen and field samples:** State if relevant **permits** obtained, provide details of authority approving study; if none were required, explain why.	Not Applicable	

Dual Use Research of Concern (DURC)	Information included in the manuscript?	In which section is the information available? (Reagents and Tools Table, Materials and Methods, Figures, Data Availability Section)
Could your study fall under dual use research restrictions? Please check biosecurity documents and list of **select agents and toxins** (CDC): https://www.selectagents.gov/sat/list.htm	Not Applicable	
If you used a select agent, is the security level of the lab appropriate and reported in the manuscript?	Not Applicable	
If a study is subject to dual use research of concern regulations, is the name of the **authority granting approval and reference number** for the regulatory approval provided in the manuscript?	Not Applicable	

Reporting

The MDAR framework recommends adoption of discipline-specific guidelines, established and endorsed through community initiatives. Journals have their own policy about requiring specific guidelines and recommendations to complement MDAR.

Adherence to community standards	Information included in the manuscript?	In which section is the information available? (Reagents and Tools Table, Materials and Methods, Figures, Data Availability Section)
State if relevant guidelines or checklists (e.g., **ICMJE, MIBBI, ARRIVE, PRISMA**) have been followed or provided.	Not Applicable	
For **tumor marker prognostic studies,** we recommend that you follow the **REMARK** reporting guidelines (see link list at top right). See author guidelines, under 'Reporting Guidelines'. Please confirm you have followed these guidelines.	Not Applicable	
For **phase II and III randomized controlled trials**, please refer to the **CONSORT** flow diagram (see link list at top right) and submit the CONSORT checklist (see link list at top right) with your submission. See author guidelines, under 'Reporting Guidelines'. Please confirm you have submitted this list.	Not Applicable	

Data Availability

Data availability	Information included in the manuscript?	In which section is the information available? (Reagents and Tools Table, Materials and Methods, Figures, Data Availability Section)
Have **primary datasets** been deposited according to the journal's guidelines (see 'Data Deposition' section) and the respective accession numbers provided in the Data Availability Section?	Yes	Data availability section
Were **human clinical and genomic datasets** deposited in a public access-controlled repository in accordance to ethical obligations to the patients and to the applicable consent agreement?	Not Applicable	
Are **computational models** that are central and integral to a study available without restrictions in a machine-readable form? Were the relevant accession numbers or links provided?	Not Applicable	
If publicly available data were reused, provide the respective **data citations in the reference list**.	Yes	Results, Figure Legends
