# Supplementary material for: Mannose supports lung cancer metabolism during glycolytic limitation
Source: EMBO Rep. 2026 Aug 7;27(18):5365–81. doi: 10.1038/s44319-026-00874-6 (PMC13601547; doi:10.1038/s44319-026-00874-6)
Supplement: Supplementary file 2 [file 44319_2026_874_MOESM2_ESM.pdf]

Appendix for:

Mannose supports lung cancer metabolism during glycolytic limitation

Table of content

Appendix Figure S1. Effect of 2-DG treatment on metabolism and proliferation of lung cancer cells. 2

Appendix Figure S2. Total abundance of metabolites in lung cancer cells treated with 2-DG or glucose starvation. 3-4

Appendix Figure S3. Mannose is used by glucose-deprived lung cancer cells. 5

Appendix Figure S4. Stable isotope tracing with ^{13}C-mannose in mouse xenografts. 6

Appendix Figure S5. Utilization of mannose for glycosylation and the glycolysis pathway by lung tumors *in vivo*. 7-8

Appendix Figure S6. ^{13}C-mannose tracing in lung, liver and blood of mice bearing xenografts. 9

Appendix Figure S7. ER stress rescue in lung cancer cells by exogenous mannose. 10

Appendix Figure S8. FACS gating strategy. 11

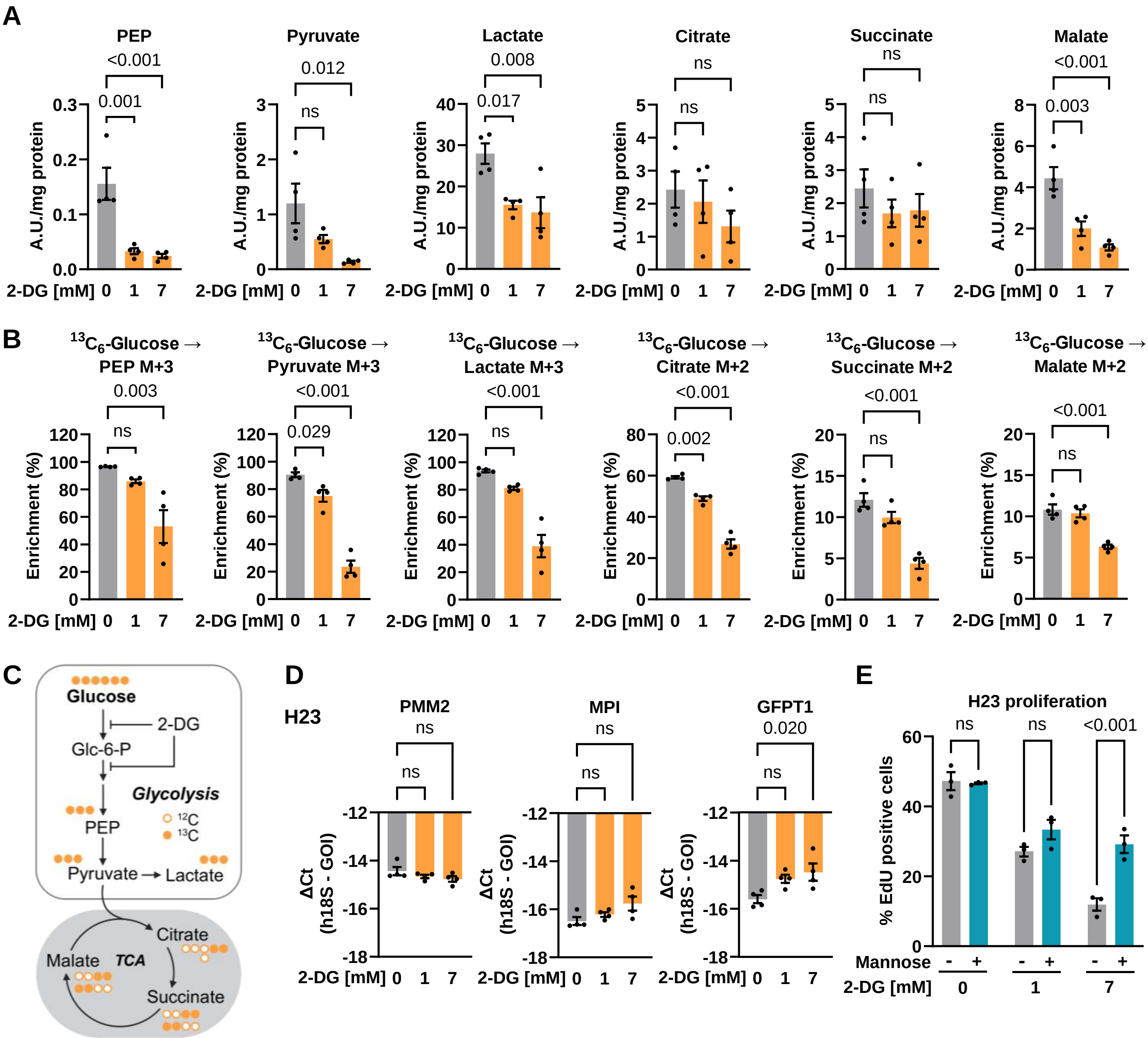

Appendix Figure S1. Effect of 2-DG treatment on metabolism and proliferation of lung cancer cells. (**A**) Total abundance and (**B**) enrichment (%) of the primary labeled isotopologue of glycolytic and TCA cycle intermediates in H23 cells treated with 2-deoxyglucose (2-DG). After 24 hours, media was supplemented with 7 mM $^{13}C_6$-glucose as a tracer for another 24 hours (*n=4*). (**C**) Metabolic downstream pathways of $^{13}C_6$-glucose. Glc-6-P, glucose-6-phosphate; PEP, phosphoenolpyruvate; TCA, tricarboxylic acid cycle. (**D**) Expression of genes involved in mannose metabolism in H23 cells treated with 2-DG for 24 hours. ΔCt-values were calculated as human 18S ribosomal RNA (h18S, reference gene) minus gene of interest (GOI) (*n=4*). PMM2, phosphomannomutase 2; MPI, mannose-6 phosphate isomerase; GFPT1, glutamine-fructose-6-phosphate transaminase 1. (**E**) Proliferation rate (% EdU positive cells) of H23 cells treated with 2-DG in combination +/- 100 µM mannose for 48 hours (*n=3*). Data are represented as mean +/- SEM from biological replicates (*n*). Statistical significance was determined by (**A**,**B**,**D**) one-way ANOVA with Dunnett's post-hoc analysis or (**E**) two-way ANOVA with Šidák's multiple comparison test; p-values are provided in the graphs; ns, not significant. Illustration in **C** was created with BioRender.

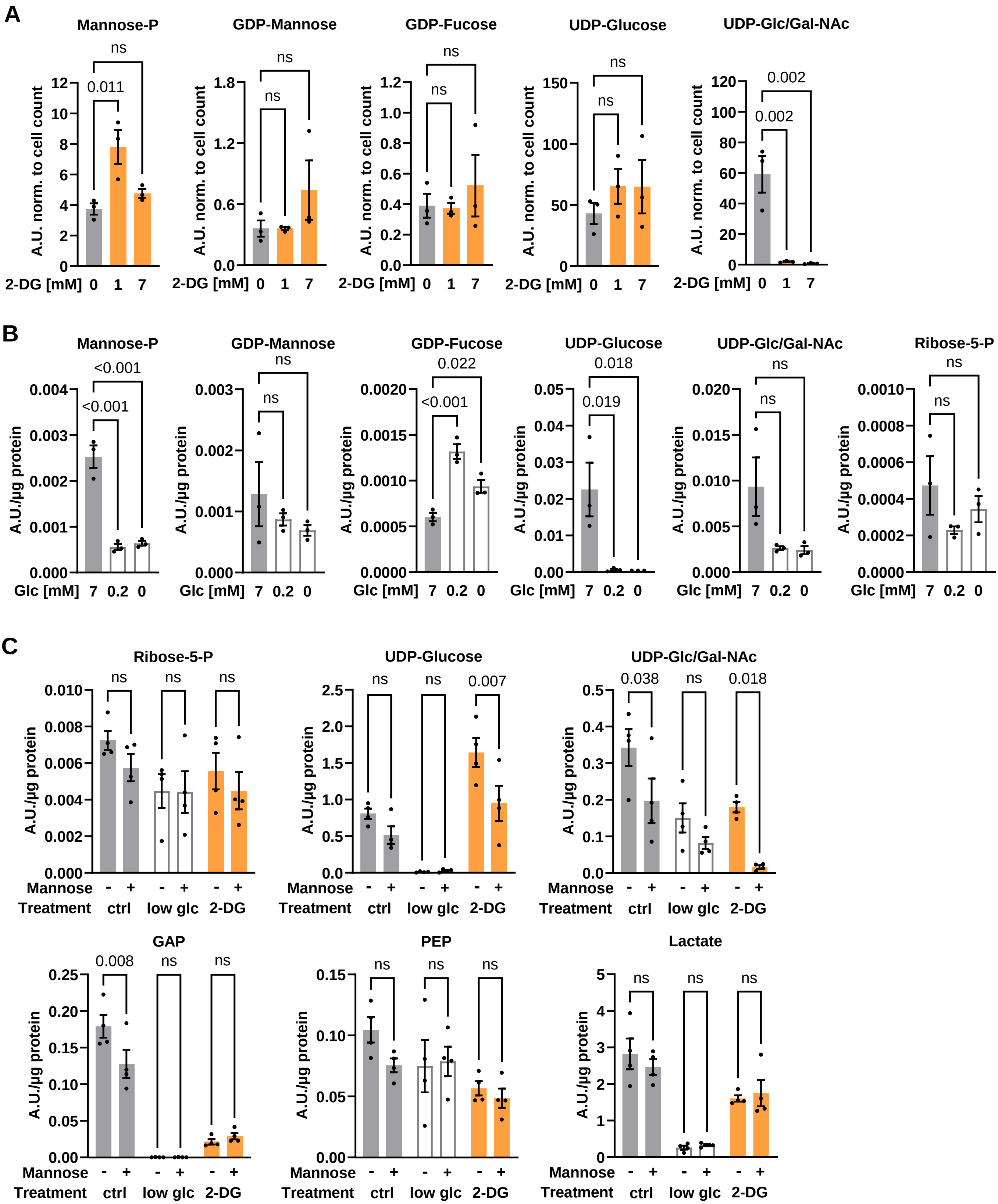

Appendix Figure S2. Figure caption on next page.

Appendix Figure S2. Total abundance of metabolites in lung cancer cells treated with 2-DG or glucose starvation. Total metabolite abundance in A549 cells treated with 100 µM $^{13}C_6$-mannose and (**A**) 2-DG or (**B**) glucose (glc) starvation media for 24 hours. Analysis was done by liquid chromatography-mass spectrometry (LC-MS) (*n=3*). Different LC-MS platforms were used for panel **A** and **B**. Mannose-6-phosphate/mannose-1-phosphate is depicted as mannose-P and UDP-GlcNAc/UDP-GalNAc as UDP-Glc/Gal-NAc (not distinguishable with our LC-MS methods). GDP, guanosine diphosphate; UDP, uridine diphosphate; UDP-GlcNAc, UDP-N-acetylglucosamine; UDP-GalNAc, UDP-N-acetylgalactosamine. (**C**) Total abundance of metabolites in A549 cells after treatment with media containing 1 mM 2-DG or low glucose (0.2 mM) in combination +/- 100 µM mannose. Cells were treated for 24 hours and analyzed by LC-MS (*n=4*). GAP, glyceraldehyde 3-phosphate; PEP, phosphoenolpyruvate. Data are shown as mean +/- SEM from biological replicates (*n*). Statistical significance was determined by (**A**,**B**) one-way ANOVA with Dunnett's post-hoc analysis or (**D**) two-way ANOVA with Šidák's multiple comparison test; p-values are provided in the graphs; ns, not significant.

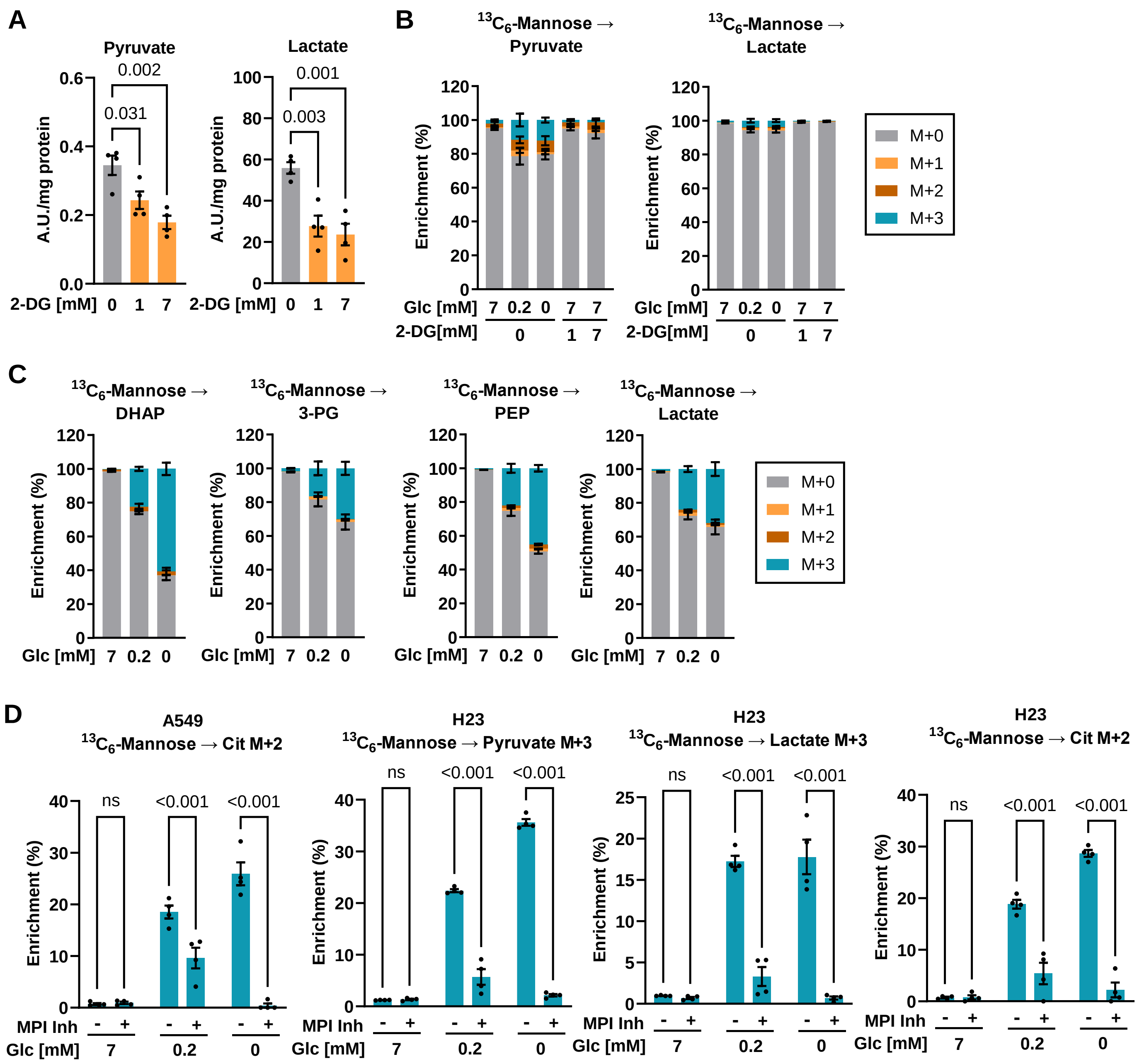

Appendix Figure S3. Mannose is used by glucose-deprived lung cancer cells. (**A**) Total abundance of pyruvate and lactate in A549 cells treated with 2-DG and 100 µM $^{13}C_6$-mannose for 24 hours (*n=4*), measured using gas chromatography-mass spectrometry (GC-MS) analysis. (**B**) Relative enrichment of all isotopologues per metabolite in A549 cells treated with 2-deoxyglucose (2-DG) or glucose starvation media and 100 µM $^{13}C_6$-mannose for 24 hours, analyzed by GC-MS (*n=4*). (**C**) Enrichment (%) of all metabolite isotopologues in glucose starved A549 cells measured by liquid chromatography-mass spectrometry (LC-MS) (*n=3*). DHAP, dihydroxyacetone phosphate; 3-PG, 3-phosphoglycerate; PEP, phosphoenolpyruvate. (**D**) Enrichment (%) of the primarily labeled form of glycolytic intermediates (M+3 for pyruvate and lactate, M+2 for citrate) in A549 and H23 cells treated with glucose starvation media +/- 50 µM of the MPI inhibitor MLS0315771 or DMSO (control) and 100 µM $^{13}C_6$-mannose for 24 hours (*n=4*). Data are represented as mean +/- SEM from biological replicates (*n*). Statistical significance was determined by (**A**) one-way ANOVA with Dunnett's post-hoc analysis or (**D**) two-way ANOVA with Šidák's multiple comparison test; p-values are provided in the graphs; ns, not significant.

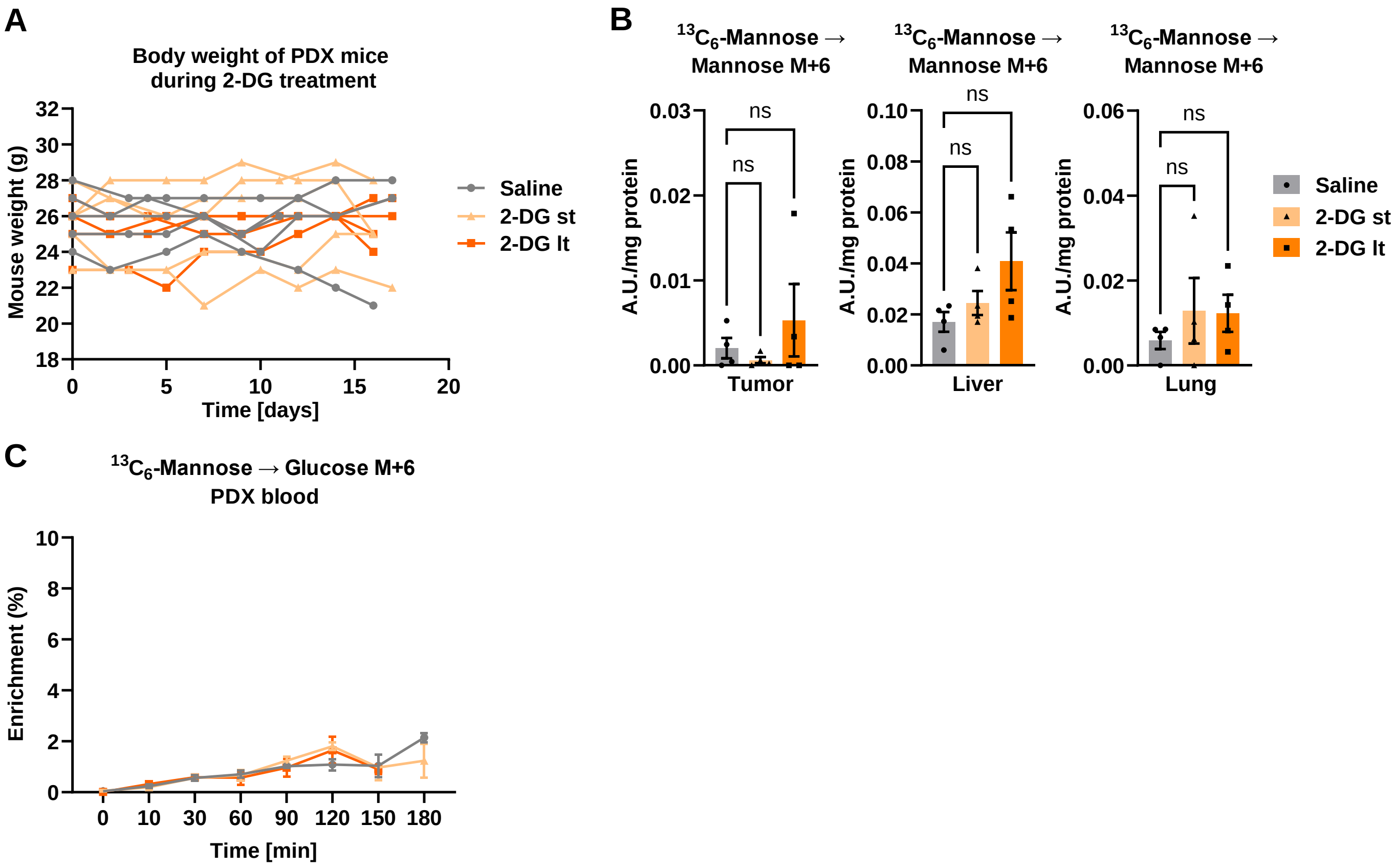

Appendix Figure S4. Stable isotope tracing with ^{13}C-mannose in mouse xenografts. (**A**) Body weight of patient-derived xenograft mice during treatment with saline (control), long-term (lt) 2-DG (500 mg/kg 2-DG, 3x/week for 2 weeks) or short-term (st) 2-DG (single dose of 500 mg/kg 2-DG right before infusion), intraperitoneally. Lines represent independent mice. (**B**) Total abundance of labeled mannose (M+6) in the subcutaneous tumor, liver and lung of 2-DG treated mouse xenografts infused with $^{13}C_6$-mannose (*n=4*). (**C**) Enrichment (%) of fully ^{13}C-labeled glucose (M+6) in the blood of 2-DG treated mouse xenografts over the time of infusion (*n=2-5;* lower sample size in later timepoints due to early death during infusion). Data represented as mean +/- SEM of biological replicates (*n*). Statistical significance was determined by one-way ANOVA with Dunnett's post-hoc analysis (**B**). ns, not significant.

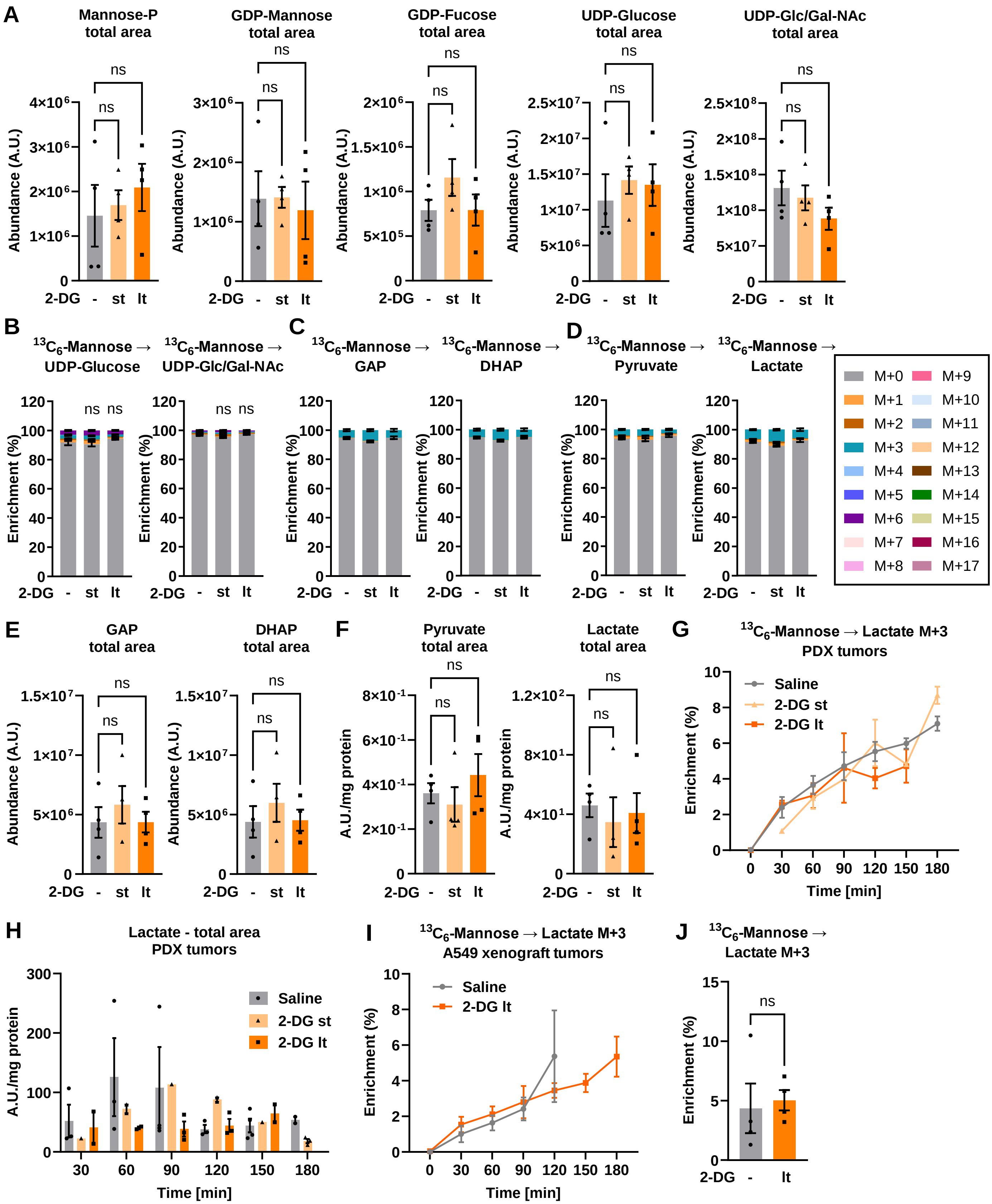

Appendix Figure S5. Figure caption on next page.

Appendix Figure S5. Utilization of mannose for glycosylation and the glycolysis pathway by lung tumors *in vivo*. (**A**-**H**) Mice carrying patient-derived or A549 xenografts were either treated with 2-DG (long term, lt; short term, st) or saline and infused with $^{13}C_6$-mannose. (**A**) Total metabolite abundance in patient-derived xenograft (PDX) tumors treated with 2-DG and infused with $^{13}C_6$-mannose, analyzed by liquid chromatography-mass spectrometry (LC-MS) (*n=4*). Mannose-6-phosphate/mannose-1-phosphate is depicted as mannose-P and UDP-GlcNAc/UDP-GalNAc as UDP-Glc/Gal-NAc (not distinguishable with our LC-MS methods). GDP, guanosine diphosphate; UDP, uridine diphosphate; UDP-GlcNAc, UDP-N-acetylglucosamine; UDP-GalNAc, UDP-N-acetylgalactosamine. (**B**) Isotopologue enrichment (%) of metabolites in 2-DG treated and $^{13}C_6$-mannose infused PDX tumors, analyzed by LC-MS (*n=4*). (**C**,**D**) Isotopologue enrichment (%) of glycolytic intermediates and (**E**,**F**) their total abundance in PDX tumors treated with 2-DG and infused with $^{13}C_6$-mannose (*n=4*). Analysis was performed by LC-MS (**C**,**E**) or gas chromatography-mass spectrometry (GC-MS, **D**,**F**). DHAP, dihydroxyacetone phosphate; GAP, glyceraldehyde 3-phosphate. (**G**) Enrichment (%) of fully ^{13}C-labeled lactate (M+3) and (**H**) total lactate abundance in PDX tumors over the time of infusion (*n=2-4*, *n=1* for timepoint 0 in saline and 2-DG lt as well as timepoints 30, 90 and 150 in 2-DG st treated mice; sample size was limited because not all tumors were suitable for serial sections). (**I**) Enrichment (%) of fully ^{13}C-labeled lactate (M+3) in A549 xenograft tumors over the time of infusion (*n=3-4*, *n=1* for timepoint 0; sample size was limited because not all tumors were suitable for serial sections) and (**J**) endpoint of infusion with $^{13}C_6$-mannose (*n=4*). Data shown as mean +/- SEM of biological replicates (*n*). Statistical significance was determined by (**A,E,F**) one-way ANOVA with Dunnett's post-hoc analysis, (**B**) one-way ANOVA with Dunnett's post-hoc analysis versus control condition of the M+6 labeled isotopologue, or (**J**) unpaired Student's t-test. ns, not significant.

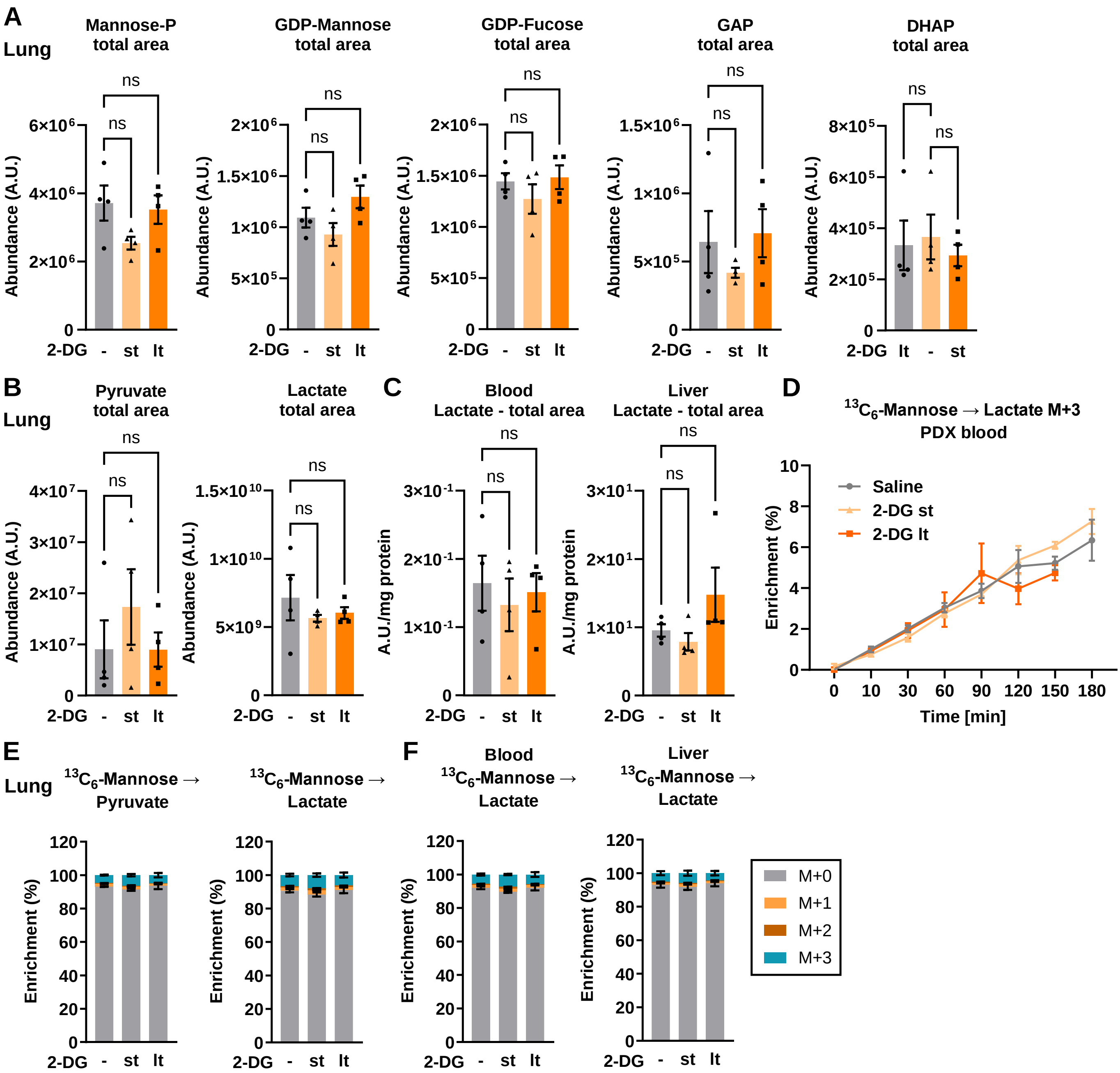

Appendix Figure S6. ^{13}C-mannose tracing in lung, liver and blood of mice bearing xenografts. (**A**-**F**) Mice carrying patient-derived xenografts (PDX) were either treated with 2-DG (long term, lt; short term, st) or saline and infused with $^{13}C_6$-mannose. (**A**,**B**) Total abundance of metabolites in lungs of $^{13}C_6$-mannose infused PDX mice analyzed by liquid chromatography-mass spectrometry (LC-MS) (*n=4*). Mannose-6-phosphate/mannose-1-phosphate is depicted as mannose-P (not distinguishable with our LC-MS method). GDP, guanosine diphosphate; DHAP, dihydroxyacetone phosphate; GAP, glyceraldehyde 3-phosphate. (**C**) Total abundance of lactate in blood and liver of 2-DG treated PDX mice at the end of infusion with $^{13}C_6$-mannose measured by gas chromatography-mass spectrometry (GC-MS) (*n=4*). (**D**) Enrichment (%) of fully ^{13}C-labeled lactate (M+3) in the blood of 2-DG treated PDX mice over the time of infusion analyzed by GC-MS (*n=2-5;* lower sample size in later timepoints due to early death during infusion). (**E**,**F**) Enrichment (%) of glycolytic metabolites in lung, blood, and liver of 2-DG treated and $^{13}C_6$-mannose infused PDX mice (*n=4*). Samples were measured using (**E**) LC-MS or (**F**) GC-MS analysis. Data are represented as mean +/- SEM of biological replicates (*n*). (**A**-**C**) Statistical significance was determined by one-way ANOVA with Dunnett's post-hoc analysis. ns, not significant.

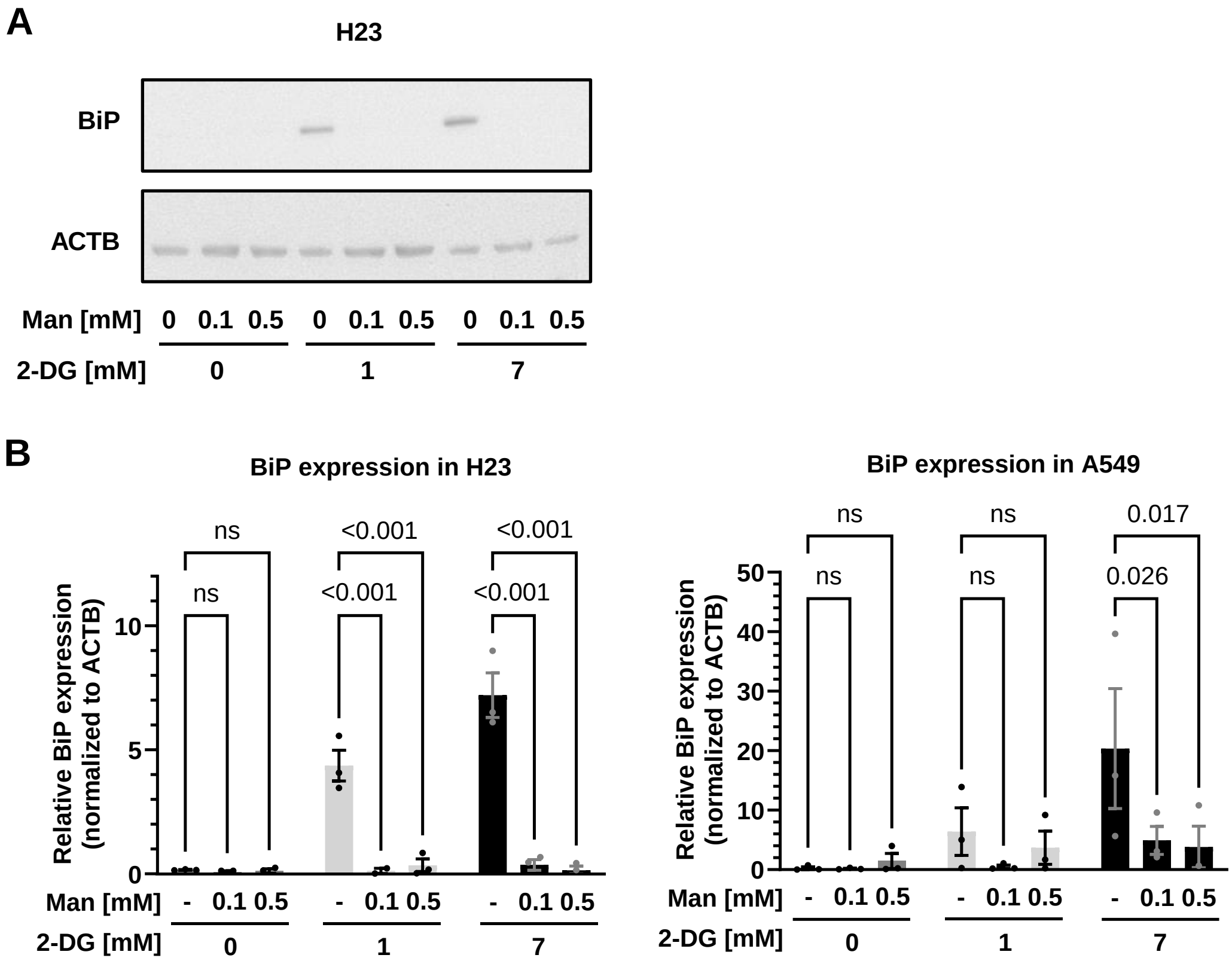

Appendix Figure S7. ER stress rescue in lung cancer cells by exogenous mannose. (**A**) Protein expression of binding immunoglobulin protein (BiP, marker for endoplasmic reticulum stress) and beta-actin (ACTB, loading control) in H23 cells treated with 2-DG and mannose for 48 hours. Representative Western blots are shown. (**B**) Quantification of BiP protein expression (normalized to beta-actin) in lung cancer cells treated with 2-DG and mannose for 48 hours (*n=3*). The Image Lab Software (Bio-Rad) was used to quantify the signal intensity of detected bands. Data are represented as mean +/- SEM from biological replicates (*n*). Statistical significance was determined by two-way ANOVA with Dunnett's post-hoc analysis; p-values are provided in the graphs; ns, not significant.

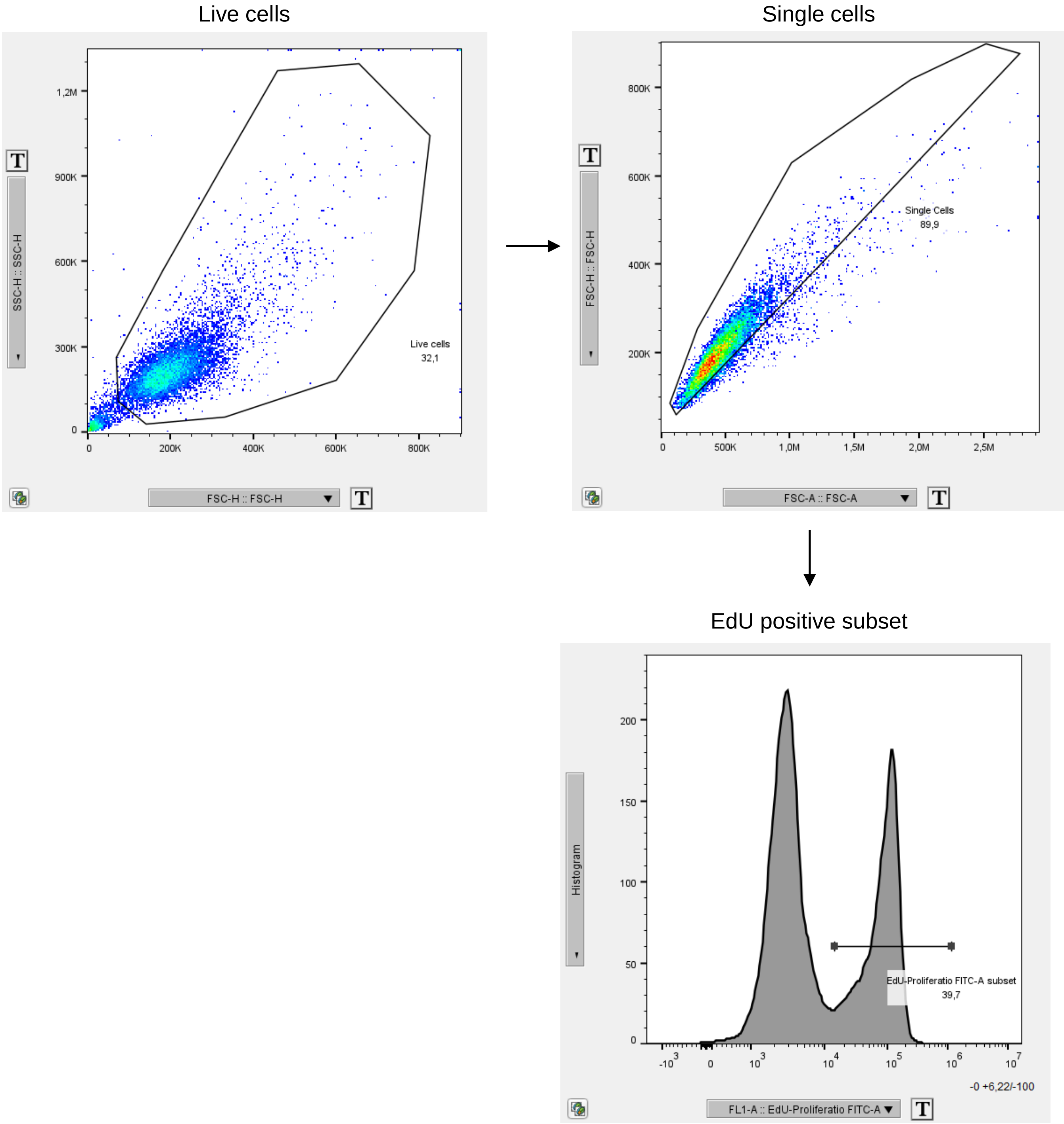

Appendix Figure S8. FACS gating strategy. Samples were first gated for live cells and single cells, then for Click-iT EdU Alexa Fluor 488 fluorescence (EdU positive cells). Representative gating of A549 cells treated with medium containing 7 mM glucose and 10% dialyzed fetal bovine serum for 48 hours is shown. SSC, side scatter; FSC, forward scatter; A, area; H, height.
